# Allocation of intensive care resources during an infectious disease outbreak: a rapid review to inform practice

**DOI:** 10.1186/s12916-020-01871-9

**Published:** 2020-12-18

**Authors:** Kirsten M. Fiest, Karla D. Krewulak, Kara M. Plotnikoff, Laryssa G. Kemp, Ken Kuljit S. Parhar, Daniel J. Niven, John B. Kortbeek, Henry T. Stelfox, Jeanna Parsons Leigh

**Affiliations:** 1 Department of Critical Care Medicine, Cumming School of Medicine, University of Calgary & Alberta Health Services, 3134 Hospital Drive NW, Calgary, T2N4Z6 Canada; 2 Department of Community Health Sciences and O’Brien Institute for Public Health, Cumming School of Medicine, University of Calgary, 3134 Hospital Drive NW, Calgary, T2N4Z6 Canada; 3 Department of Surgery, Cumming School of Medicine, University of Calgary & Alberta Health Services, 3134 Hospital Drive NW, Calgary, T2N4Z6 Canada; 4 Department of Anaesthesia, Cumming School of Medicine, University of Calgary & Alberta Health Services, 3134 Hospital Drive NW, Calgary, T2N4Z6 Canada; 5 Faculty of Health, School of Health Administration, Dalhousie University, 5850 College Street, Halifax, Nova Scotia B3H4R2 Canada; 6 Department of Critical Care Medicine, Faculty of Medicine, Dalhousie University, 6299 South St, Halifax, Nova Scotia B3H4R2 Canada

**Keywords:** Triage, Critical care, Intensive care, Resource allocation, Medical ethics, Practice guideline, COVID-19

## Abstract

**Background:**

The COVID-19 pandemic has placed sustained demand on health systems globally, and the capacity to provide critical care has been overwhelmed in some jurisdictions. It is unknown which triage criteria for allocation of resources perform best to inform health system decision-making. We sought to summarize and describe existing triage tools and ethical frameworks to aid healthcare decision-making during infectious disease outbreaks.

**Methods:**

We conducted a rapid review of triage criteria and ethical frameworks for the allocation of critical care resources during epidemics and pandemics. We searched Medline, EMBASE, and SCOPUS from inception to November 3, 2020. Full-text screening and data abstraction were conducted independently and in duplicate by three reviewers. Articles were included if they were primary research, an adult critical care setting, and the framework described was related to an infectious disease outbreak. We summarized each triage tool and ethical guidelines or framework including their elements and operating characteristics using descriptive statistics. We assessed the quality of each article with applicable checklists tailored to each study design.

**Results:**

From 11,539 unique citations, 697 full-text articles were reviewed and 83 articles were included. Fifty-nine described critical care triage protocols and 25 described ethical frameworks. Of these, four articles described both a protocol and ethical framework. Sixty articles described 52 unique triage criteria (29 algorithm-based, 23 point-based). Few algorithmic- or point-based triage protocols were good predictors of mortality with AUCs ranging from 0.51 (PMEWS) to 0.85 (admitting SOFA > 11). Most published triage protocols included the substantive values of duty to provide care, equity, stewardship and trust, and the procedural value of reason.

**Conclusions:**

This review summarizes available triage protocols and ethical guidelines to provide decision-makers with data to help select and tailor triage tools. Given the uncertainty about how the COVID-19 pandemic will progress and any future pandemics, jurisdictions should prepare by selecting and adapting a triage tool that works best for their circumstances.

**Supplementary Information:**

The online version contains supplementary material available at 10.1186/s12916-020-01871-9.

## Background

Five pandemics occurred in the last century: H1N1 (1918), H2N2 (1957–1958), H3N2 influenza (1968), the H1N1 virus (2009) [1–3], and the novel coronavirus SARS-CoV-2 (COVID-19) (2020) [4]. Viral infections caused by influenza or coronavirus may lead to organ failure, including respiratory illness, which can progress to hypoxemic respiratory failure and the acute respiratory distress syndrome, requiring admission to an intensive care unit (ICU) [5]. ICUs are specialized units wherein highly trained specialists work with a multidisciplinary team to deliver life sustaining therapies [6]. The availability of ICU beds varies across countries ranging from 0 (low-income countries such as South Sudan) to 59.5 (high-income countries such as Monaco) adult ICU beds per 100,000 population [7]. With the world’s population over 7.8 billion [8], rapid viral spread during a pandemic can overwhelm ICU resources [9, 10] (e.g., staff, beds, ventilators, extracorporeal life support).

It is essential that hospitals have a strategy to ensure equitable and ethical resource allocation if the demand for ICU resources exceeds supply [11]. One such strategy is a triage protocol, which is a set of criteria that are enacted during resource scarcity to determine which patients should be admitted to an ICU or continue to receive care in an ICU. Recent reviews discuss the principle of triage and implementation of triage plans [12], quality of criteria to triage and transfer critically ill patients [13], and ethical principles used in disaster response [14, 15]. However, it is unclear which triage criteria perform best. In light of the ongoing COVID-19 pandemic, we conducted a rapid review of the published triage literature to evaluate the validity of published triage protocols and the mortality prediction embedded within them to help inform health system-decision-making.

## Methods

We conducted a rapid review to ensure timeliness of data in response to the COVID-19 pandemic and adhered to the Preferred Reporting Items for Systematic Reviews and Meta-Analyses standards where possible (as no guideline for rapid reviews exists) [16] (Supplementary Appendix 2, Additional File 1). The search strategy included the following: (1) terms for critical care (e.g., ICU, intensive care, mechanical ventilation); (2) terms for pandemics, epidemics, and infectious disease outbreaks (e.g., avian influenza, Severe Acute Respiratory Syndrome [SARS], Middle East Respiratory Syndrome [MERS], COVID-19); and (3) terms for triage criteria, resource allocation, and ethical frameworks (e.g., triage, resource allocation) (see Supplementary Appendix 2, Additional File 1). The search was executed on November 3, 2020, in three databases: MEDLINE (Ovid), EMBASE (Ovid), and SCOPUS (Elsevier). References were exported and managed using EndNote X9 (Clarivate Analytics).

To ensure inter-rater agreement, a random sample of 10% of included articles were pilot tested independently by three reviewers (KK, KP, LK) with 92% agreement. The remaining titles and abstracts were screened independently by one of three reviewers (KK, KP, LK). Studies were included if they were published in the English language and were (1) primary research and other research, including consensus-based study designs; (2) targeted critically ill adults (i.e., patients with life-threatening respiratory, cardiovascular, or neurological illness or other illnesses necessitating life sustaining therapies only provided in an ICU and are ≥ 18 years old [or an adult as defined by the study]); and (3) described development or evaluation of triage criteria used on all patients (e.g., COVID and non-COVID) to allocate critical care resources during an infectious disease outbreak. Citations deemed eligible by any reviewer at the title and abstract stage were included for full-text review. Full-text articles were similarly screened independently and in duplicate by three reviewers (KK, KP, LK). Disagreements were resolved by discussion or consensus with a fourth reviewer (KF). Reference lists were examined for any additional relevant studies not identified in the original search.

The following data were recorded on a standardized form in Excel: study information (e.g., country of study conduct, year published, study design), participant characteristics (e.g., study population, sample size), and outcomes (e.g., triage guideline creation, evaluation of triage criteria, and triage protocols). Data are reported using descriptive statistics.

The quality of included quantitative studies was assessed using the Joanna Briggs Institute Critical Appraisal tools for case control, cohort, cross-sectional, diagnostic test accuracy, quasi-experimental, qualitative, or text and opinion studies [17–22]. The quality of Delphi techniques was assessed based on proposed recommendations by Jünger and colleagues. The quality of guidelines was assessed using the 23-item AGREE Reporting Checklist. In all cases, a higher number indicated better methodological quality.

## Results

**Fig. 1:**
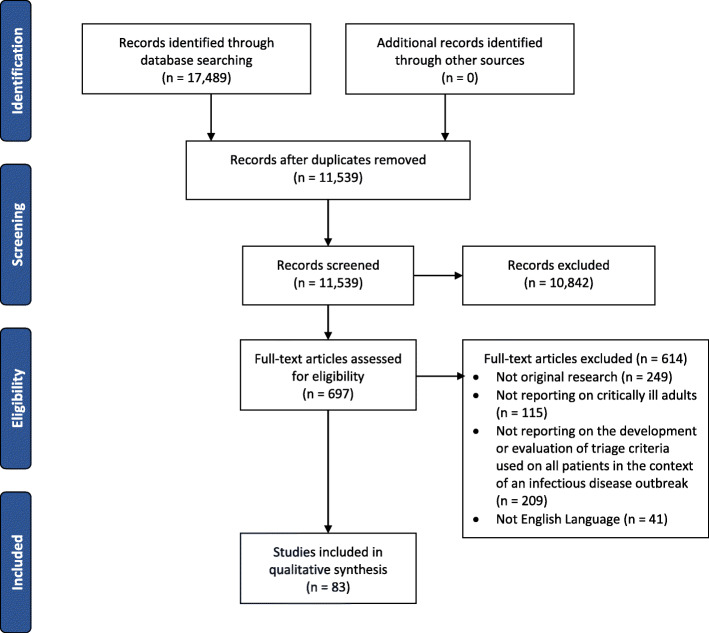
Preferred Reporting Items for Systematic Reviews and Meta-Analyses (PRISMA) diagram of study selection

The literature search identified 11,539 unique citations (Fig. 1). After assessing titles and abstracts for eligibility, 697 articles were included for full-text review. Eighty-three articles were included in the final rapid review. The most common reasons for exclusion included not original research (249/614, 40.6%) or not reporting on the development or evaluation of triage criteria used on all patients in the context of an infectious disease outbreak (209/614, 34.0%(Fig. 1).

Supplementary Table 1, Additional File 1 presents the characteristics of the 83 included articles, published from 2005 to 2020. Studies were conducted in North America (43/83, 51.8%), Europe (24/83, 28.9%), Oceania (6/83, 7.2%), across multiple continents (5/83, 6.0%), Asia (4/83, 4.8%), and one was unclear (1/83, 1.2%). Observational studies included retrospective cohort (17/83, 20.5%), prospective cohort (8/83, 9.6%), cross-sectional (4/83, 4.8%), mixed methods/computer modeling (each 2/83, 2.4%), and pre-post/qualitative (each 1/83, 1.2%). Several studies were categorized as text and opinion (48/83, 57.8%), which was further defined as studies that include research evidence such as working groups, expert opinions, consensus, or current discourse for the purposes of this review.

Among the 83 included articles, 52 unique critical care triage protocols from 59 articles and 25 ethical frameworks or recommendations from 25 articles were described. Four articles included both a triage protocol and an ethical framework [23–25]. Two articles were computer simulation models and were not assessed as a triage protocol or ethical framework [26, 27]. The 52 included critical care triage protocols have two main approaches: algorithmic (29/52, 55.8%) or point-based (23/52, 44.2%) (Supplementary Table 2, Additional File 1). In the algorithmic approach, each patient is assessed for each criterion, which determines if the patient is eligible to receive critical care. For example, the Ontario Health Plan for an Influenza Pandemic (OHPIP) has four main components: inclusion criteria (patients who require critical resources), exclusion criteria (patients who have poor prognosis), minimum qualifications for survival (amount of resources required), and a prioritization tool (color scheme to prioritize patients for ICU admission and mechanical ventilation) [28] (Supplementary Table 2, Additional File 1). Some algorithmic triage protocols are conceptually similar (7/29, 24.1%) (Supplementary Table 2, Additional File 1). Few articles (10/29, 34.5%) [29–37] describe supplemental criteria for situations where triage decisions cannot be reached after evaluating patients based on the criteria. These criteria included fair innings (5/10, 50%), random selection (3/10, 30%), multiplier effect/essential worker/caregiver/healthcare provider/first come, and first served/groups subjected to social deprivation and disadvantage (each 2/10, 20%) (Supplementary Table 1). The Hamilton Health Adult Critical Care Triage and Resource Allocation Protocol includes additional criteria for situations where triage decisions cannot be reached after evaluating patients based on the OHPIP triage protocol (e.g., clinical evidence of a significantly better outcome, multiplier effect which is those with skills and knowledge to save others during the pandemic) [36]. The article by Winsor et al. summarizes common “supplementary criteria” and included a list of key considerations when jurisdictions are considering supplementary triage criteria.

Most included algorithm triage protocols (18/29, 62.1%) included a Sequential Organ Failure Assessment (SOFA) score as exclusion criterion. Of these, 10 (10/18, 55.6%) proposed a SOFA score of > 11 to exclude patients from critical care resources [25, 28, 30, 34, 35, 38–42]. Seven did not state a cutoff [29, 31, 43–47], and one proposed a SOFA score ≥ 14 or ≤ 8 [48]. Only four (4/29, 13.8%) triage protocols included a criterion for patient age (> 85 years) as an exclusion criterion [28, 34, 49, 50]. Seven (7/29, 24.1%) include frailty as a criterion [29, 42, 44, 46, 51–53]. Most triage protocols (19/29, 65.5%) include a re-assessment and re-categorization of each patient (e.g., at 48 and 120 h) to determine if patients are improving and are still eligible for critical care [28–33, 35, 38, 39, 42–44, 46–49, 53, 54].

Twenty-three unique point-based triage protocols were described in the included studies (Supplementary Table 2, Additional File 1). In a point-based triage approach, scores are used to determine who in the case-mix of patients should receive critical care resources. Of the 23 point-based triage protocols, eight (8/23, 34.5%) included triage protocols developed for an infectious disease outbreaks [55–66] (AGILITIES, Clinical Dashboard, Community Assessment Tools [CAT], Critical Care Disaster Resource Allocation Framework, Pandemic Modified Warning Score [P-MEWS], Simple Triage Scoring System [STSS], Swine Flu Triage [SWiFT], and XGBoost Machine Learning Algorithm). Six (6/23, 26.1%) included validated predictors of mortality (CURB-65, Nursing Home-Acquired Pneumonia in the Elderly [NHAP], Predisposition Insult Response and Organ Dysfunction [PIRO-CAP], Pneumonia Severity Index [PSI] and Severity Score for the Elderly with Community-Acquired Pneumonia) [60, 67–69] or intensive respiratory or vasopressor support (SMART-COP) in patients with community acquired pneumonia. Five (5/23, 21.7%) are commonly used scoring systems for severity of illness in the ICU or to determine if a patient requires critical care intervention and include the Acute Physiology And Chronic Health Evaluation-II/III (APACHE-II/APACHE-III) [70, 71], Modified Early Warning System (MEWS) [66], SOFA [61, 70–76], quick SOFA (qSOFA) [66], or Simplified Acute Physiology Score (SAPS-II) [71]. Two included a scoring system for severe hypoxemic respiratory failure from acute respiratory distress (Murray score, PaO_2_/FiO_2_ ratio) [63, 77]. One included a predictor of mortality for sepsis (Mortality in Emergency Department Sepsis [MEDS]) [60, 63]. Most point-based triage protocols included the patient’s age (12/23, 52.2%) (Table 1) that is gender specific (PSI-men: points in years, women: points in years- 10), includes age categories (e.g., 0–49, 50–69, 70–84, ≥ 85), a multiplier (e.g., 0.5x), or an age cutoff (e.g., ≥ 65, ≥ 75, ≥ 85). In all cases, the older the patient is, the more points they are assigned (i.e., less favorable). Nearly all point-based triage protocols (20/23, 87%%) included vital signs, with the most commonly included being respiratory rate (18/23, 78.3%), blood pressure (14/23, 60.9%), and heart rate (12/23, 52.2%). Nearly all point-based triage protocols included laboratory or radiographic findings, with oxygenation (14/23, 60.9%%) the most commonly included criterion. One point-based triage protocol deducted points (i.e., were more favorable) for patients who were pregnant [58].

**Table 1:** Categories of criteria included in published point-based triage protocols

Point-based criteria	Age	Comorbidities	Vital signs	Laboratory findings	Neurological status
*Pandemic*					
AGILITIES Score	✓	✓	✓ ^b,e^	✓^1^	✓(GCS)
CAT	✗	✗	✓ ^b,^	✗	✓(ALOC)
Clinical dashboard	✓	✓	✓^a,b,c,e^	✓^6,8,11–16^	✗
Critical Care Disaster Resource Allocation	✓	✓	✓ ^a,b^	✓^1,9,10^	✗
P-MEWS	✗	✗	✓ ^a,b,c,d^	✗	✗
STSS	✓	✗	✓ ^b^	✓^1^	✓(mental status)
SWiFT	✗	✗	✓ ^a,b,c,d^	✓^1^	✓(mental status)
XGBoost Machine Learning Algorithm	✓	✗	✓ ^a,b,c,d^	✓^6,8,10^	✗
*Community-acquired pneumonia*					
CURB-65	✓	✗	✓ ^a,b^	✓^6^	✓(mental status)
NHAP	✗	✗	✓ ^b,c^	✗	✓(mental status)
PIRO-CAP	✓	✓	✗	✓^1,6^	✗
PSI	✓	✓	✓ ^a,b,c,d^	✓^1,2,3,5,6,7^	✓(mental status)
Severity Score for the Elderly with CAP	✓	✓	✓ ^a,b,c,d,e^	✓^6^	✗
SMART-COP	✗	✗	✓ ^a,b,c^	✓^1^	✓(mental status)
*ECMO allocation strategy*					
Minnesota Department of Health	✓	✗	✗	✓^1,9,10^	✓(GCS)
*ICU scoring systems*					
APACHE-II	✓	✗	✓ ^a,b,c,d^	✓^1,2,3,4,6,7,8^	✓(GCS)
MEWS	✗	✗	✓ ^a,b,c,d^	✗	✓(AVPU)
qSOFA	✗	✗	✓ ^a,b^	✗	✓(GCS)
SAPS-II	✓	✓	✓ ^a,c,d^	✓^1,2,3,4,6,8^	✓(GCS)
SOFA	✗	✗	✗	✓^1,9,10^	✓(GCS)
*Sepsis*					
MEDS	✓	✓	✓ ^b^	✓^8,9^	✓(mental status)
*ARDS*					
Murray score	✗	✗	✗	✓^1^	✗
PaO_2_/FiO_2_ ratio	✗	✗	✗	✓^1^	✗

*Abbreviations*: *AGILITIES* Age, Glasgow score, Infusions, Lungs, Interventions, Tests, Informal/incidental, Excessive weight, Subtract; *ALOC* altered level of consciousness; *APACHE-II* Acute Physiology and Chronic Health Evaluation; *CAP* community-acquired pneumonia; *CAT* Community Assessment Tools; *CURB-65* Confusion, Urea, Respiratory rate, Blood pressure, Age ≥ 65; *GCS* Glasgow Coma Scale; *MEDS* Mortality in Emergency Department Sepsis; *MEWS* Modified Early Warning Score; *NHAP* Nursing Home-Acquired Pneumonia in the Elderly; *P-MEWS* Pandemic Modified Warning Score; *PIRO-CAP* Predisposition Insult Response and Organ Dysfunction in Community Acquired Pneumonia; *PSI* Pneumonia Severity Index; *qSOFA* quick Sequential Organ Failure Assessment; *SAPS-II* Simplified Acute Physiology Score; *SOFA* Sequential Organ Failure Assessment; *SMART-COP* Systolic blood pressure, Multilobar involvement, Respiratory rate, Tachycardia, Confusion, Oxygenation; *STSS* Simple Triage Scoring System; *SWiFT* Swine Flu Triage

Vital signs included: a-blood pressure, b-respiratory rate, c-heart rate, d-temperature, e-height/weight/BMI

Laboratory findings included: 1-oxygenation, 2-arterial pH or HCO3, 3-sodium (serum), 4-potassium (serum), 5-glucose, 6-kidney (creatinine, BUN, urine output), 7-hematocrit, 8-WBC, 9-platelet, 10-bilirubin, 11-procalcitonin, 12-D-dimer, 13-C-reactive protein, 14-LDH, 15-troponin, 16-ferritin

Sixteen of the included studies described the accuracy of 18 critical care triage protocols (Supplementary Table 3, Additional File 1) to predict mortality (13/16, 81.2%)(Table 2) [57, 59, 60, 62, 66, 67, 69, 71, 72, 74, 75, 78], ICU admission (10/16, 62.5%), and need for mechanical ventilation (6/16, 37.5%) (Supplementary Table 3, Additional File 1) [61–63, 72, 74]. Four of these studies evaluated the performance of an algorithm-based triage protocol [76, 78, 79], with three evaluating the OHPIP [76, 78, 79]. Of the patients who met the criteria for OHPIP blue (i.e., palliative care only and would not be admitted to the ICU), 24.6–63.0% died and 27.3–75.4% survived [63, 76, 78, 79]. Of the patients who met the criteria for OHPIP red (i.e., high priority for ICU admission), 65–93.7% survived and 6.3–35.0% died (Table 2). One article used a machine-learning based algorithm, which predicted mortality in all ICU patients (AUROC 0.862) more accurately compared to the qSOFA (0.760), MEWS (0.833), and CURB-65 (0.652) [66]. The remaining articles evaluated the performance of point-based triage protocols. Five articles evaluated four triage protocols developed for an infectious disease outbreak: CAT, P-MEWS, STSS, and SWiFT. No score was a good predictor of mortality with AUCs ranging from 0.58 (95% CI 0.46–0.69; P-MEWS) to 0.71 (95% CI 0.66–0.77; STSS) (Supplementary Table 3, Additional File 1). Six included articles evaluated scoring systems for severity of illness in the ICU: APACHE-II/APACHE-III, MEWS, qSOFA/SOFA, and SAPS-II (Supplementary Table 3, Additional File 1). The SOFA score on admission was a good predictor of 30-day mortality in one study with an AUC of 0.83 (95% CI 0.81–0.85]) [72]. Two studies reported that a SOFA score > 11 was a fair predictor of mortality (AUC 0.74 and 0.65) [70, 78]. Six studies reported in-hospital mortality in patients with a SOFA score > 11, which ranged from 0% [73] to 59% (95% CI 56–62%) [75] (Table 2). Four included articles evaluated performance of predictors of mortality in community acquired pneumonia for triage of patients during a pandemic [57, 60, 67, 69]. No score was a good predictor of mortality with AUCs ranging from 0.64 (95% CI 0.58–0.71; PIRO-CAP) to 0.78 (95% CI 0.72–0.83; PSI).

**Table 2:** Diagnostic accuracy of triage tools to predict mortality

Triage tool (mortality risk)	Hospital mortality, % (per triage tool)	Patients meeting triage criteria, %	Key operating characteristics
**Algorithmic**			
Machine Learning Algorithm (12-h window) [66]	9.6	NR	AUC: 0.75–0.86 Se: 0.80 Sp: 0.75
OHPIP blue [63, 76, 78, 79]	10.9–29.1	18.8–27.8	AUC: 0.816 Se: 0.76 Sp: 0.91 TP (%): 24.6–63.0 FP (%): 27.3–75.4
OHPIP red	NR	19.8–43.9	AUC/Se/Sp: NR TN (%): 65.0–93.7 FN (%): 6.3–35.0
OHPIP yellow	NR	4.0–11.4	AUC/Se/Sp: NR TN (%): 41.4–75.0 FN (%): 25–58.6
OHPIP green	NR	0–65.3	AUC: NR Se: 0.66 Sp: 0.83 TP (%): 0–4.5 FP (%): 95.5–100
**Point-based (severity of illness)**			
MSOFA > 11 [72]	10.5	4.0	AUC: 0.84 Se/Sp: NR TP (%): 57.7 FP (%): 42.3
MSOFA 8–11	NR	14.6	AUC/Se/Sp: NR TN (%): 69.1 FN (%): 30.9
qSOFA [66]	9.6	NR	AUC: 0.76 Se: 0.95 Sp: 0.37
MEWS [66]	9.6	NR	AUC: 0.83 Se: 0.90 Sp: 0.56
SOFA > 11 [61, 70, 72, 75]	4.8–20.5	5.9–40	AUC: 0.64–0.85 Se/Sp: NR TP (%): 0–59.0 FP (%): 41–100
SOFA 8–11	NR	12.2–44.0	AUC/Se/Sp: NR TN (%): 72.2–83.6 FN (%): 16.4–27.8
**Point-based (epidemic or pandemic)**			
CAT ≥ 3 [57]	6.0	18.8	AUC: 0.65 Se: 0.47 Sp: 0.83 TN (%): 85.2 FN (%): 14.8
CAT < 3	NR	81.2	AUC/Se/Sp: NR TP (%): 3.9 FP (%): 96.0
PMEWS > 3 [57, 59, 60]	6.0–22.6	62.8–70.4	AUC: 0.60–0.66 Se: 0.87 Sp: 0.16 TN (%): 74.0–93.4 FN (%): 6.6–26
STSS [60–63]	4.7–12.3	NA	AUC: 0.71–0.80 Se/Sp: NR
STSS 0	NR	30.6–44.1	AUC: 0.71 Se/Sp: NR TN (%): 94.7–99.6 FN (%): 0.4–5.3
STSS 1	NR	31.7–42.7	AUC/Se/Sp: NR TN (%): 90.1–96.4 FN (%): 3.6–9.9
STSS 2	NR	14.8–23.8	AUC/Se/Sp: NR TN (%): 70.8–84.6 FN (%): 15.4–29.2
STSS ≥ 3	NR	5.9–14.5	AUC/Se/Sp: NR TN (%): 63.8–89.9 FN (%): 11.1–36.2
SwiFT [55]	NR	NR	AUC: 0.77 Se/Sp: NR
**Point-based (sepsis)**			
MEDS > 5 [60, 64]	10.9–12.0	NR	AUC: 0.77 Se: 0.85 Sp: 0.40
MEDS ≤ 5	NR	NR	AUC/Se/Sp: NR TP (%): 4.8 FP (%): 95.2
**Point-based (community acquired pneumonia)**			
CAP [57]	11.7	NR	AUC: 0.66 Se/Sp: NR
CURB-65 [57, 59, 60, 66–69]	6.0–31.7	NR	AUC: 0.65–0.79 Se: 0.97–0.98 Sp: 0.16–0.18
CURB-65 0–1 (low)	NR	36.9–37.6	AUC: 0.67 Se: 0.94 Sp: 0.17 TP (%): 0–22.1 FP (%): 77.9–100
CURB-65 2 (moderate)	NR	18.1	AUC: 0.64 Se: 0.48–0.73 Sp: 0.50–0.78 TN (%): 66.7–88.3 FN (%):11.7–33.3
CURB-65 ≥ 3 (severe)	NR	0.1–4.6	AUC: 0.54 Se: 0–0.36 Sp: 0.84–0.99 TN (%): 33.3–100 FN (%): 0–66.7
NHAP [60]	NR	NR	AUC: 0.68 Se/Sp: NR
PIRO-CAP [69]	31.7	NR	AUC: 0.64 Se/Sp: NR
PIRO-CAP 0–2 (low)	NR	27.5	AUC: NR Sen 0.94–0.96 Sp: 0.07–17.1 TN (%): 86.3 FN (%): 13.7
PIRO-CAP 3 (moderate)	NR	29.1	AUC: NR Se: 0.88 Sp: 0.35 TN (%): 67.5 FN (%): 32.5
PIRO-CAP 4 (high)	NR	25.3	AUC: NR Se: 0.58 Sp: 0.63 TN (%): 59.7 FN (%):40.3
PIRO-CAP ≥ 5 (very high)	NR	18.1	AUC: NR Se: 0.01–0.26 Sp: 0.86–0.99 TN (%): 54.2 FN (%): 45.8
PSI > 87 [60, 67–69] (low)	12.0–31.7	41.9–64.0	AUC: 0.73–0.78 Se: 46.4–92.9 Sp: 35.4–80.1 TP (%): 13.5–17.4 FP (%): 82.6–86.5 TN (%): 55.2–97.7 FN (%): 2.3–44.8
PSI ≤ 87 (high)	NR	NR	AUC: NR Se: 93 Sp: 39
SMRT-CO(P) [60, 67]	NR	NR	AUC: 0.69 Se/Sp: NR
SMRT-CO(P) 0–2 (low)	11.7	11–39	AUC/Se/Sp: NR
SMRT-CO(P) 3 (moderate)	NR	17	AUC/Se/Sp: NR
SMRT-CO(P) ≥ 4 (high)	NR	11	AUC/Se/Sp: NR

*Abbreviations*: *AUC* area under the curve; *Se* sensitivity; *Sp* specificity; *TP* true positive; *FP* false positive; *TN* true negative; *FN* false negative; *CAP* Severity Score for the Elderly with Community-Acquired Pneumonia; *CATS* Community Assessment Tools; *CI* confidence interval; *CURB-65* Confusion, Urea, Respiratory rate, Blood pressure, age ≥ 65; *MEDS* Mortality in Emergency Department Sepsis; *MSOFA* modified Sepsis-related Organ Failure Assessment; *NA* not applicable; *NHAP* Nursing Home-Acquired Pneumonia in the Elderly; *NR* not reported; *PMEWS* Pandemic Medical Early Warning Score; *OHPIP* Ontario Health Plan for an Influenza Epidemic; *PIRO-CAP* Predisposition, Insult, Response, and Organ Dysfunction in Community-Acquired Pneumonia; *PSI* Pneumonia Severity Index; *SMART-COP* or *SMRT-CO* Systolic blood pressure, Multilobar involvement, Respiratory rate, Tachycardia, Confusion, Oxygenation; *SOFA* Sepsis-related Organ Failure Assessment; *STSS* Simple Triage Scoring System; *SWiFT* Swine Flu Triage

Thirty of the included articles explicitly stated which ethical principles guided the development of the triage criteria. Of these, twelve articles described ethical frameworks (12/30, 40%) and 13 described guiding principles or recommendations (13/30, 43.3%) for triage decisions during a pandemic (Supplementary Table 4, Additional File 1). Four articles used an ethical framework or recommendation to inform the development of a triage protocol [23–25, 32]. Although terminology differed across the studies, they can be divided between substantive values (e.g., duty to provide care, equity, stewardship, etc.) and procedural values (e.g., reasonable, open and transparent, etc.) [80] (Table 3). For example, most included the principle of stewardship (20/30, 66.7%) in their criteria [10, 24, 35, 83, 85–88, 90, 92–94, 97–100]. This included the duty to steward scarce resources through the principle of utilitarianism (i.e., helping the greatest number of patients to survive the pandemic). Several ethical frameworks or guiding principles included the procedural value of transparency, with 14 (14/30, 46.7%) recommending that triage criteria be disclosed to the public or developed with input from the public [10, 11, 23, 29, 32, 35, 37, 46, 82, 84, 85, 88, 92, 93, 95, 98]. Four studies [88, 93, 95, 98] engaged the public to prioritize triage criteria. In all studies, the criterion “most likely to survive” was perceived as fair or was prioritized. The criterion “first come, first served” was perceived as unfair or prioritized in the bottom half of the triage criteria [32, 58, 87, 88, 98]. Only five studies (5/30, 16.7%) supported allocation of resources based on societal contributions or reciprocity [10, 88, 90, 93, 98].

**Table 3:** Substantive and procedural values in published ethical frameworks and guiding principles for critical care triage during a pandemic (*n* = 30)

	Example	***n*** (%)	Author (year published)
***Substantive values***			
Distributive justice or fairness	Fairness in how resources are allocated across members of a group.	8 (26.7)	Farrell (2020) [81]; Han (2020) [35]; Leclerc (2020) [42]; Seethala and Keller (2020) [82]; Rhodes (2020) [83]; Steinberg (2020) [84]; Vergano (2020) [53]; Vincent and Creteur (2020) [50]
Duty to plan	Planning for the management of ethical issues that may arise.	2 (6.7)	Han (2020) [35]; Committee (2020) [47]
Duty to provide care	“Palliative care protocol (when patient does not qualify for critical care allocation).”; “The concept of triage by a senior clinician(s) without direct clinical obligation and a support system to implement and manage the triage process.”	13 (43.3)	Christian (2010) [85]; Christian (2014) [11]; Cinti (2009) [86]; Daugherty Biddison (2014) [87, 88]; Devereaux (2008) [89]; Eastman (2010) [90]; Einav (2014) [91]; Han (2020) [35]; Herrerros (2020); Lin and Anderson-Shaw (2009) [23]; Powell (2008) [24]; Rubinson (2005) [92]; Silva (2012) [93]
Equality	“Triage decisions will not be based on race, ethnicity, gender, disability, insurance status, immigration status, social class, or other non-clinical factors.”	4 (13.3)	Marckmann (2020) [46]; Montgomery (2020) [52]; Rhodes (2020) [83]; Steinberg (2020) [84]
Equity	“Triage decisions during the epidemic should apply to all patients who may require intensive care, not just COVID-19 patients.”	17 (56.7)	Cheung (2017) [94, 95]; Christian (2010) [85]; Christian (2014) [11]; Cinti (2009) [86]; Devereaux (2008) [89]; Dries (2014) [96]; Daugherty Biddison (2014) [87, 88]; Eastman (2010) [90]; Emanuel [10]; Herrerros (2020); Leclerc (2020) [42]; Powel (2008) [24]; Tillyard (2010) [97]; Silva (2012) [93]; Real de Asua (2020) [32]; Rhodes (2020) [83]; Steinberg (2020) [84]
Reciprocity	Resources allocated based on societal contributions (e.g., caregivers, healthcare providers - feelings of reciprocity).	5 (16.7)	Daugherty Biddison (2014) [87, 88]; Daugherty Biddison (2018) [98]; Eastman (2010) [90]; Emanuel [10]; Silva (2012) [93]
Stewardship	“Triage decisions regarding the provision of critical care should be guided by the principle of seeking to help the greatest number of people survive the crisis.”	20 (66.7)	Cheung (2017) [94, 95]; Christian (2010) [85]; Christian (2014) [11]; Cinti (2009) [86]; Daugherty Biddison (2014) [87, 88]; Daugherty Biddison (2014) [87, 88]; Daugherty Biddison (2018) [98]; Eastman (2010) [90]; Emanual (2020); Han (2020) [35]; Janig (2020) [30]; Marckmann (2020) [46]; Powell (2008) [24]; Rubinson (2005) [92]; Silva (2012) [93]; Steinberg (2020) [84]; Tabery and Mackett (2008) [99]; Real de Asua (2020) [32]; Rhodes (2020) [83]; Tillyard (2010) [97]
Trust	“Review of triage decisions (daily retrospective review).”	10 (33.3)	Christian (2014) [11]; Chung (2017); Cinti (2009) [86]; Daugherty Biddison (2014) [87, 88]; Daugherty Biddison (2018) [98]; Lin and Anderson-Shaw (2009) [23]; Powell (2008) [24]; Rubinson (2005) [92]; Silva (2012) [93]; Tabery and Mackett (2008) [99]
***Procedural values***			
Reasonable	Critical care resources be allocated based on specific triage criteria, irrespective of whether the need for resources is related to the current disaster/pandemic or an unrelated critical illness or injury.	12 (40)	Cheung (2017) [94, 95]; Christian (2010) [85]; Christian (2014) [11]; Daugherty Biddison (2014) [87, 88]; Devereaux [89]; Dries (2014) [96]; Eastman (2010) [90]; Einav (2014) [91]; Lin and Anderson-Shaw (2009) [23]; Powell (2008) [24]; Rubinson (2005) [92]; Silva (2012) [93]
Open and transparent	“The criteria for triage should be transparent, public, and as shared as possible. The triage process is the responsibility of the entire society.”	14 (46.7)	Cheung (2017) [94, 95]; Christian (2010) [85]; Christian (2014) [11]; Daugherty Biddison (2014) [87, 88]; Daugherty Biddison (2018) [98]; Han (2020) [35]; Lin and Anderson-Shaw (2009) [23]; Marckmann (2020) [46]; Real de Asua (2020) [32]; Rubinson (2005) [92]; Silva (2012) [93]; Seethala and Keller (2020) [82]; Steinberg (2020) [84]; Valiani (2020) [29]
Inclusive	“Hospitals should establish procedure in advance of a crisis. These protocols should be developed regionally and with input from stakeholders (including the public).”	7 (23.3)	Cheung (2017) [94, 95]; Christian (2010) [85]; Christian (2014) [11]; Daugherty Biddison (2014) [87, 88]; Daugherty Biddison (2018) [98]; Rubinson (2005) [92]; Silva (2012) [93]
Responsive	“Prioritization guidelines should differ by intervention and should respond to changing scientific evidence.”	4 (13.3)	Devereaux (2008) [89]; Emmanuael (2020); Real de Asua (2020) [32]; Valuani (2020)
Accountable	“Triage Review Board-to oversee switch from traditional ethics of individual autonomy to an ethics of public health.”	5 (16.7)	Christian (2014) [11]; Lin and Anderson-Shaw (2009) [23]; Seethala and Keller (2020) [82]; Tabery and Mackett (2008) [99]; Valiani (2020) [29]

Several included papers described when triage criteria should be initiated, who should administer the triage criteria, an appeals process, if load leveling is possible to distribute patients throughout a region and consider vulnerable populations. Most included articles suggested that triage criteria be initiated when critical care resources have been overwhelmed despite all efforts to expand critical care capacity to meet demands [10, 11, 25, 29, 31, 32, 36, 40, 44, 46, 47, 50, 53, 58, 82–85, 89, 90, 92, 98, 101]. Some articles suggested that triage criteria be administered by a triage officer who is an intensivist or other physician with appropriate critical care experience and has no direct contact with the patient [10, 11, 33, 35, 37, 38, 79, 81, 85, 89]. Several articles suggested a triage team or committee to remove the burden of the decision from a single individual [23, 25, 29–32, 34–36, 47, 51, 52, 65, 83, 93, 102]. This triage team may be important when decisions using supplementary criteria are involved [34, 103] or if there are appeals from family or clinicians [23, 24, 92, 93]. This triage team is recommended to include a senior ICU physician, non-physician ICU healthcare professional, and a professional from outside the ICU. This triage process may be overseen by a triage review board [99] or include prospective (i.e., during an appeals process) or retrospective review to ensure accountability, consistent application of the triage criteria, and an adequate level of prioritization (i.e., not over or under triaging) [11, 33, 34, 42, 89]. Several articles suggested that an appeals process is not feasible given time and resource constraints [24, 36, 89]. Some articles recommend including an appeal process, but limiting appeals to deviations from the triage process or reevaluations due to updated clinical information [10, 11]. Articles discussed a regionalization scheme for highly sophisticated critical care (e.g., ECMO) [77] or transferring patients to less strained ICUs [38, 42]. When withdrawing critical care from one patient to reallocate it to another patient with a better prognosis [98], articles outline the ethical [24] and legal challenges associated [90]. Some articles described the importance of educating the public on triage criteria and early family involvement in decision-making as a way to potentially mitigate some of the issues raised by families when decisions to withhold care are made [23, 24, 92, 93]. Several articles advocated for the consideration of vulnerable populations [96] to ensure that medical treatment in the context of a pandemic is not restricted to those able to pay [10] or that allocation decisions do not replicating existing inequities (e.g., insured vs. uninsured, urban vs. rural) [98]. Moreover, secondary triage factors may be necessary for selecting among patients with similar prognosis. In this case, the fairest option in the included articles was randomization versus first-come first-served, which penalized people of lower socio-economic means or minority populations [10, 35]. Several articles recommend the use of advanced age as triage criterion [45], while others reject the use of age and instead encourage frailty, which takes into consideration functional and cognitive status and burden of comorbidities [35, 42, 44, 50, 81].

### Quality analysis

Of 83 included articles, 80 were evaluated for quality (Table 4). Three articles could not be assessed because we could not find quality criteria for evaluating studies that included a computer-generated study population or development of an intelligence dashboard [26, 27, 65]. Five included studies were published conference abstracts and, as such, we did not have enough methodological information to determine their risk of bias. The remaining 75 full-text articles were deemed of sufficient quality to include in analyses (Table 4).

**Table 4:** Quality analysis of included articles

Author (year)	Critical appraisal tool	Criteria met, ***n***/total (%)	Overall appraisal (reason)
Adalja (2013) [77]	JBI-text & opinion	5/6 (83.3)*	Include
Adeniji and Cusack (2011)	JBI-cohort	7/11 (63.6)	Include
Ardagh (2006) [101]	JBI-text & opinion	3/6 (50.0)	Include
Ashton-Cleary (2011) [40]	JBI-cross-sectional	6/8 (75.0)	Include
Azoulay (2020) [44]	JBI-text & opinion	6/6 (100)	Include
Barie (2020) [124]	JBI-text & opinion	5/6 (83.3)	Include
Brandao-Neto [125]	JBI-cohort	7/11 (63.6)*	Include
Challen (2007)	JBI-cohort	8/11 (72.7)	Include
Cheung (2012)-*MJA* [39]	JBI-cohort	9/11 (81.8)	Include
Cheung (2012)-*Crit Care Resusc* [48]	JBI-cohort	9/11 (81.8)	Include
Cheung (2017) [94, 95]	JBI-cross-sectional	6/8 (75.0)	Include
Christian (2006) [28]	JBI-text & opinion	5/6 (83.3)*	Include
Christian (2009) [79]	JBI-cohort	8/11 (72.7)	Include
Christian (2010) [85]	Delphi process	5/11 (45.5)	Include
Christian (2014) [11]	Delphi process	10/11 (90.9)	Include
Cinti (2009) [86]	JBI-text & opinion	6/6 (100)	Include
Commons and Denholm (2012) [67]	JBI-diagnostic test accuracy	5/10 (50.0)	Include
Daugherty Biddison (2014)-*CHEST* [88]	Delphi process	10/11 (90.9)	Include
Daugherty Biddison (2014)-*Annals ATS* [87]	JBI-qualitative JBI-quasi-experimental	6/10 (60.0) 5/9 (55.6)*	Include
Daugherty Biddison (2018) [98]	JBI-qualitative JBI-quasi-experimental	8/10 (80.0) 5/9 (55.6)*	Include
Daugherty Biddison (2019) [58]	JBI-text & opinion	5/6 (83.3)*	Include
Devereaux (2008) [89]	JBI-text & opinion	5/6 (83.3)*	Include
Dries (2014) [96]	Delphi process	10/11 (90.9)	Include
Eastman (2010) [90]	JBI-text & opinion	6/6 (100)	Include
Ehmann (2020) [31]	JBI-text & opinion	6/6 (100)	Include
Einav (2014) [91]	Delphi process	10/11 (90.9)	Include
Emanuel [10]	JBI-text & opinion	5/6 (83.3)*	Include
Enfield (2011) [70]	JBI-cohort	6/11 (54.5)	Seek further info (conference abstract)
Estella (2012) [68]	JBI-cohort	9/11 (81.8)	Include
Farrell (2020) [81]	JBI-text & opinion	6/6 (100)	Include
Frolic (2009) [36]	AGREE Reporting Checklist	14/23 (60.9)	Include
Grissom (2010) [72]	JBI-cohort	9/11 (81.8)	Include
Guest (2009) [78]	JBI-cohort	7/11 (63.6)	Include
Gupta (2020) [51]	JBI-text & opinion	5/6 (83.3)	Include
Han (2020) [35]	JBI-text & opinion	6/6 (100)	Include
Herreros (2020) [45]	JBI-text & opinion	6/6 (100)	Include
Hick (2006) [43]	JBI-text & opinion	5/6 (83.3)*	Include
Ibrahim (2020) [65]	*No critical appraisal for development of intelligence dashboard*	*Not applicable*	Include
Janig (2020) [30]	JBI-text & opinion	6/6 (100)	Include
Kanter (2015) [27]	*No critical appraisal for modeling data*	*Not applicable*	Include
Kaposy (2010) [34]	JBI-text & opinion	5/6 (83.3)	Include
Khan (2009) [73]	JBI-cohort	7/11 (63.6)	Include
Leclerc (2020) [42]	JBI-text & opinion	6/6 (100)	Include
Lin and Anderson-Shaw (2009) [23]	JBI-text & opinion	5/6 (83.3)*	Include
Marckmann (2020) [46]	JBI-text & opinion	6/6 (100)	Include
Marriott (2012) [41]	JBI-cohort	6/11 (54.5)	Seek further info (conference abstract)
Miller (2010) [74]	JBI-cohort	7/11 (63.6)	Seek further info (conference abstract)
Montgomery (2020) [52]	JBI-text & opinion	4/6 (66.7)*	Include
Morton (2014) [63]	JBI-cohort	8/11 (72.7)	Seek further info (conference abstract)
Morton (2015) [65]	JBI-cohort	8/11 (72.7)	Include
Muller (2010) [60]	JBI-cohort	9/11 (81.8)	Include
Murad (2009) [54]	JBI-cross-sectional	4/8 (50.0)	Include
Myles (2012) [57]	JBI-case control	9/10 (90.0)	Include
PCP Ethic Committee (2020) [47]	JBI-text & opinion	3/6 (50.0)	Include
Pereira (2012) [69]	JBI-cohort	9/11 (81.8)	Include
Powell (2008) [24]	AGREE Reporting Checklist	12/23 (52.2)	Include
Prekker (2020) [25]	JBI-text & opinion	5/6 (83.3)*	Include
Real de Asua (2020) [32]	JBI-text & opinion	5/6 (83.3)	Include
Rhodes (2020) [83]	JBI-text & opinion	5/6 (83.3)	Include
Rowan (2010) [55]	JBI-cohort	8/11 (72.7)	Include
Rubinson (2005) [92]	JBI-text & opinion	4/6 (66.7)*	Include
Ryan (2020) [66]	JBI-cohort	7/11 (63.6)	Include
Saleh (2016) [71]	JBI-cohort	6/11 (54.5)	Seek further info (conference abstract)
Seethala and Keller (2020) [82]	JBI-text & opinion	6/6 (100)	Include
Shahpori (2011) [75]	JBI-cohort	10/11 (90.9)	Include
Shekar (2020) [102]	JBI-text & opinion	6/6 (100)	Include
Silva (2012) [93]	JBI-qualitative	8/10 (80.0)	Include
Sprung (2010) [38]	Delphi process	6/11 (54.5)	Include
Sprung (2020) [33]	JBI-text & opinion	5/6 (83.3)	Include
Steinberg (2020) [84]	JBI-text & opinion	6/6 (100)	Include
Swiss Academy of Medical Sciences & Swiss Society of Intensive Care (2020) [49]	AGREE Reporting Checklist	12/23 (52.2)	Include
Tabery and Mackett (2008) [99]	JBI-text & opinion	6/6 (100)	Include
Talmor (2007) [62]	JBI-cohort	10/11 (90.9)	Include
Tambone (2020) [107]	JBI-text & opinion	4/6 (66.7)	Include
Tillyard (2010) [97]	JBI-text & opinion	5/6 (83.3)	Include
Utley (2011) [26]	*No critical appraisal for modeling data*	*Not applicable*	Include
Valiani (2020) [29]	JBI-text & opinion	6/6 (100)	Include
Vergano (2020) [53]	JBI-text & opinion	4/6 (66.7)	Include
Vincent and Creteur (2020) [50]	JBI-text & opinion	2/6 (33.3)	Include
Warrillow (2020) [37]	JBI-text & opinion	5/6 (83.3)	Include
Wilkens and Klein (2010) [56]	JBI-cross-sectional	4/8 (50.0)	Include
Williams and Gannon (2009) [76]	JBI-cohort	7/11 (63.6)	Include
Winsor (2014) [103]	JBI-text & opinion	6/6 (100)	Include

*Abbreviations*: *JBI* Joanna Briggs Institute

*One or more items are “not applicable”

## Discussion

The current study summarizes the literature from 83 studies that described triage criteria created before, during, and after infectious disease outbreaks (e.g., H1N1, SARS, H5N1, MERS, Ebola, COVID-19). Of the 52 triage criteria from 60 articles, 29 were algorithmic-based and 23 were point-based. There were 30 articles that describe ethical frameworks or guiding principles for triage decisions. Most of the algorithm-based triage criteria are based on the OHPIP triage protocol for critical care [28] or included a SOFA score when making triage decisions. Most point-based triage protocols consider the patient’s age or vital signs in the total score. Most triage criteria have not been validated and the operating characteristics for those that have (e.g., SOFA) to predict mortality are modest.

It is expected that triage protocols should perform better than first-come, first served distribution of resources as a means to allocate scarce resources and maximize patient survival [27]. However, the results of this rapid review suggest that most protocols are not validated or do not have sufficient criterion validity to predict mortality. Validated scoring systems such as a SOFA or APACHE-II score or pneumonia scoring systems were designed for a specific population and have been demonstrated to be less accurate when used in other patient populations [97, 104–108]. A recent systematic review on prediction models for diagnosis and prognosis of COVID-19 reported that proposed models were poorly described, had a high risk of bias, and may be unreliable when used in situ [109]. Most triage protocols were developed with experts (e.g., ethicists, lawyers, healthcare professionals, decision-makers and, in some cases, members of the public) in advance of a pandemic (e.g., *CHEST* consensus statements) [11, 88, 91, 96, 100, 110] or were validated using a non-representative population (e.g., patients with COVID-19, seasonal influenza or ARDS) [111–119]. As such, most triage criteria are not validated (and it may not be possible to validate them) prior to their use. Our study adds to the existing reviews that discuss implementation of triage plans [12], ethics of triage [14], ventilator triage policies [15], and quality of criteria to triage and transfer critically ill patients [13] by providing decision-makers with data to help them select and tailor tools best for their jurisdictions.

In general, triage is grounded by utilitarian theory or “the greatest good for the greatest number” or egalitarianism or “equal distribution of resources.” Both considerations are warranted during an infectious disease outbreak as triage criteria should benefit society (utilitarian) but ensure fairness (egalitarian). As such, health systems should weigh ethical principles and decide which core values should underpin the triage criteria. In addition to identifying patients at a high risk of mortality (e.g., 80–90%), triage protocols should also separate patients into cohorts of relative prognosis (e.g., 40–50-60%) with frequent revaluation in order to apply a utilitarian framework, as opposed to queuing or randomization. This rapid review suggests that most triage criteria were developed by a panel of experts and few sought public input. Triage criteria should be anchored by the procedural value of open and transparent and the substantive value of trust by engaging the public in triage development and educating the public on triage during a pandemic. In some cases, triage criteria may include the substantive value of reciprocity. For example, the developers of the Newfoundland and Labrador Critical Care Triage protocol [34] considered Indigenous populations where the death of an elder may have a devastating impact on this community. Low-income countries (e.g., South Sudan) have a shortage of skilled critical care healthcare professionals and, as such, these healthcare professionals should be given priority for receiving scarce resources because they could assist in the care of critically ill patients after they recover [120]. Health systems need to pick triage tools that consider cultural values and ideologies while adhering to utilitarian and egalitarian principles.

The large number of patients admitted to ICU during the COVID-19 pandemic provides an opportunity to validate (retrospectively and prospectively), improve, and standardize selected triage tools. This may include evaluating how well they work as prognostic models to predict mortality and length of ICU stay or length of mechanical ventilation (i.e., utilization of resources) or the ability for each criterion or a combination of criteria identified by this rapid review (e.g., age, sex, comorbidities, vital signs, SOFA score) to act as proxies for outcomes. This is important given the uncertainty about how the current pandemic will evolve and when future pandemics will occur. This should include engaging the public to ensure that triage criteria represent societal values as well as to inform members of the public how decisions will be made [87, 92, 93, 98, 100].

There are several strengths of our rapid review. The timely synthesis of published triage criteria is important for health systems to respond to the current pandemic and prepare for future pandemics and for evidence to be generated on the utility of the currently available triage criteria to allocate scarce resources and maximize survival. The rapid review followed rigorous methodology [121] that included a search strategy created with experts, a comprehensive literature review, and all steps (full-text screening, data extraction and quality assessments) completed independently and in duplicate. There are several limitations of our rapid review that should be considered. This includes the possibility that some studies were missed in the search, during selection of studies, or because of the rapidly evolving COVID-19 literature. This also includes the exclusion of unpublished triage criteria that is available on institutional, government or society websites though most of these triage criteria either are adapted from or cite triage criteria included in this review. The current study provides a catalog of possible criteria, but with only limited data on which criteria work best. Included studies were largely developed outside of the scope of pandemics or infectious disease outbreaks, as it is difficult to know how they will perform during an infectious disease outbreak. Additionally, our review is focused on an adult critical care setting as pediatric populations use different types of triage criteria. Resource allocation and triage in pediatric groups has been explored elsewhere [122, 123]. Regardless of the operating characteristics, healthcare systems need to decide which triage protocol to enact if the demand of critical care resources exceeds supply to ensure a standardized and ethically sound approach to allocating health resources.

## Conclusion

This rapid review summarizes the existing published triage criteria used for epidemics or pandemics when ICU resources are scarce. Despite the limited validation of criteria and the modest operating characteristics of those criteria that have been validated, our study provides healthcare decision-makers with a list of available criteria, their elements, and their operating characteristics. Given the uncertainty of how the current pandemic will evolve and when future pandemics occur, all healthcare systems need to pick the criteria that work best for their circumstances. The triage process needs to adhere to utilitarian and egalitarian principles. Moreover, public engagement is key to ensure that triage criteria represent societal values and to ensure that members of the public understand how decisions will be made. Health systems and jurisdictions should validate their chosen triage criteria during the current COVID-19 pandemic to develop a new model of triage in preparation for the current and future pandemics.

## Supplementary Information

**Additional file 1: Supplementary Appendix 1.** (PRISMA checklist for systematic reviews), **Supplementary Appendix 2.** (MEDLINE search strategy), **Supplementary Table 1.** (Study characteristics), **Supplementary Table 2.** (Triage protocols based on an algorithm or point-based method), **Supplementary Table 3.** (Diagnostic accuracies of triage criteria), and **Supplementary Table 4.** (Ethical frameworks or guiding principles for triage decisions during a pandemic).

## Data Availability

Data sharing is not applicable to this article as no datasets were generated or analyzed during the current study.

## Abbreviations

APACHE-II or APACHE-III: Acute Physiology and Chronic Health Evaluation II
CAT: Community Assessment Tools
COVID-19: Novel coronavirus SARS-CoV-2
ICU: Intensive care unit
MEDS: Mortality in Emergency Department Sepsis
NHAP: Nursing Home-Acquired Pneumonia in the Elderly
OHPIP: Ontario Health Plan for an Influenza Pandemic
P-MEWS: Pandemic Modified Warning Score
PIRO-CAP: Predisposition Insult Response and Organ Dysfunction
PSI: Pneumonia Severity Index
SOFA: Sequential Organ Failure Assessment
STSS: Simple Triage Scoring System
SAPS-II: Simplified Acute Physiology Score
SWiFT: Swine Flu Triage
