# Supplementary material for: Allocation of intensive care resources during an infectious disease outbreak: a rapid review to inform practice
Source: BMC Med. 2020 Dec 18;18:404. doi: 10.1186/s12916-020-01871-9 (PMC7746486; doi:10.1186/s12916-020-01871-9)
Supplement: Supplementary file 1 — Additional file 1: Supplementary Appendix 1. (PRISMA checklist for systematic reviews), Supplementary Appendix 2. (MEDLINE search strategy), Supplementary Table 1. (Study characteristics), Supplementary Table 2. (Triage protocols based on an algorithm or point-based method), Supplementary Table 3. (Diagnostic accuracies of triage criteria), and Supplementary Table 4. (Ethical frameworks or guiding principles for triage decisions during a pandemic). [file 12916_2020_1871_MOESM1_ESM.docx]

**Allocation of Intensive Care Resources During an Infectious Disease Outbreak: A Rapid Review to Inform Practice**

Kirsten M. Fiest, PhD, Karla D. Krewulak, PhD, Kara M. Plotnikoff, MPH, Laryssa G. Kemp, MSc, Ken Kuljit S. Parhar, MD MSc, Daniel J. Niven, MD PhD, John B. Kortbeek, MD, Henry T. Stelfox, MD PhD^*^, Jeanna Parsons Leigh, PhD^*^

**Additional File**

**Supplementary Tables and Figures**

**Supplementary Appendix 1.** Preferred Reporting Items for Systematic Reviews and Meta-Analyses

| **Section/topic** | **#** | **Checklist item** | **Reported on page #** |
| --- | --- | --- | --- |
| **TITLE** | | |  |
| Title | 1 | Identify the report as a systematic review, meta-analysis, or both. | 1 |
| **ABSTRACT** | | |  |
| Structured summary | 2 | Provide a structured summary including, as applicable: background; objectives; data sources; study eligibility criteria, participants, and interventions; study appraisal and synthesis methods; results; limitations; conclusions and implications of key findings; systematic review registration number. | 2-3 |
| **INTRODUCTION** | | |  |
| Rationale | 3 | Describe the rationale for the review in the context of what is already known. | 3-4 |
| Objectives | 4 | Provide an explicit statement of questions being addressed with reference to participants, interventions, comparisons, outcomes, and study design (PICOS). | 3-4 |
| **METHODS** | | |  |
| Protocol and registration | 5 | Indicate if a review protocol exists, if and where it can be accessed (e.g., Web address), and, if available, provide registration information including registration number. | N/A |
| Eligibility criteria | 6 | Specify study characteristics (e.g., PICOS, length of follow-up) and report characteristics (e.g., years considered, language, publication status) used as criteria for eligibility, giving rationale. | 4 |
| Information sources | 7 | Describe all information sources (e.g., databases with dates of coverage, contact with study authors to identify additional studies) in the search and date last searched. | 4 |
| Search | 8 | Present full electronic search strategy for at least one database, including any limits used, such that it could be repeated. | Supplementary Appendix 2 |
| Study selection | 9 | State the process for selecting studies (i.e., screening, eligibility, included in systematic review, and, if applicable, included in the meta-analysis). | 4-5 |
| Data collection process | 10 | Describe method of data extraction from reports (e.g., piloted forms, independently, in duplicate) and any processes for obtaining and confirming data from investigators. | 5 |
| Data items | 11 | List and define all variables for which data were sought (e.g., PICOS, funding sources) and any assumptions and simplifications made. | 5 |
| Risk of bias in individual studies | 12 | Describe methods used for assessing risk of bias of individual studies (including specification of whether this was done at the study or outcome level), and how this information is to be used in any data synthesis. | 5 |
| Summary measures | 13 | State the principal summary measures (e.g., risk ratio, difference in means). | 5 |
| Synthesis of results | 14 | Describe the methods of handling data and combining results of studies, if done, including measures of consistency (e.g., I^2^) for each meta-analysis. | N/A |

| **Section/topic** | **#** | **Checklist item** | **Reported on page #** |
| --- | --- | --- | --- |
| Risk of bias across studies | 15 | Specify any assessment of risk of bias that may affect the cumulative evidence (e.g., publication bias, selective reporting within studies). | 5 |
| Additional analyses | 16 | Describe methods of additional analyses (e.g., sensitivity or subgroup analyses, meta-regression), if done, indicating which were pre-specified. | N/A |
| **RESULTS** | | |  |
| Study selection | 17 | Give numbers of studies screened, assessed for eligibility, and included in the review, with reasons for exclusions at each stage, ideally with a flow diagram. | 6; Figure 1 |
| Study characteristics | 18 | For each study, present characteristics for which data were extracted (e.g., study size, PICOS, follow-up period) and provide the citations. | 6, Suppl. Table 1 |
| Risk of bias within studies | 19 | Present data on risk of bias of each study and, if available, any outcome level assessment (see item 12). | 12; Table 4 |
| Results of individual studies | 20 | For all outcomes considered (benefits or harms), present, for each study: (a) simple summary data for each intervention group (b) effect estimates and confidence intervals, ideally with a forest plot. | 6-12, Tables 1-3 |
| Synthesis of results | 21 | Present results of each meta-analysis done, including confidence intervals and measures of consistency. | N/A |
| Risk of bias across studies | 22 | Present results of any assessment of risk of bias across studies (see Item 15). | 12; Table 4 |
| Additional analysis | 23 | Give results of additional analyses, if done (e.g., sensitivity or subgroup analyses, meta-regression [see Item 16]). | N/A |
| **DISCUSSION** | | |  |
| Summary of evidence | 24 | Summarize the main findings including the strength of evidence for each main outcome; consider their relevance to key groups (e.g., healthcare providers, users, and policy makers). | 12-15 |
| Limitations | 25 | Discuss limitations at study and outcome level (e.g., risk of bias), and at review-level (e.g., incomplete retrieval of identified research, reporting bias). | 14-15 |
| Conclusions | 26 | Provide a general interpretation of the results in the context of other evidence, and implications for future research. | 15-16 |
| **FUNDING** | | |  |
| Funding | 27 | Describe sources of funding for the systematic review and other support (e.g., supply of data); role of funders for the systematic review. | 17 |

**Supplementary Appendix 2.** Medline Search Strategy

**Medline(OVID)-November 3, 2020**

1. Critical care/ or Critical Illness/ or Intensive care units/
2. ((intensive or critical) adj2 (care* or therap*)).mp.
3. (ICU or ITU or CCU).mp.
4. ((critical* or severe or catastrophic* or acute*) adj2 (ill* or sick* or ail*)).mp
5. Respiration, Artificial/ or Ventilators, Mechanical/ or Extracorporeal Membrane Oxygenation/ or Organ Dysfunction Scores/ or Multiple Organ Failure/ or APACHE/
6. (ventilat* or extracorporeal life support or ECLS or Extracorporeal Membrane Oxygenation or ECMO or sequential organ failure assessment or SOFA or “Acute Physiology And Chronic Health Evaluation” or APACHE).mp
7. Or/1-6
8. exp Disease Outbreaks/
9. Hemorrhagic Fever, Ebola/
10. influenza a virus, h1n1 subtype/ or influenza a virus, h3n2 subtype/ or Influenza A Virus, H5N1 Subtype/
11. Influenza, Human/
12. Influenza A virus/
13. Orthomyxoviridae/ or Orthomyxoviridae Infections/
14. SARS Virus/
15. Severe Acute Respiratory Syndrome/
16. exp Coronavirus/
17. Coronavirus Infections/
18. Middle East Respiratory Syndrome Coronavirus/
19. Coronaviridae/
20. (pandemic* or epidemic* or outbreak* or out break* or ebola* or orthomyxoviridae or h1n1 or h3n2 or h5n1 or avian influenza or avian flu or swine flu or swine influenza or influenza A or influenza virus or SARS or severe acute respiratory syndrome or MERS or middle east respiratory syndrome or coronavirus* or Wuhan or 2019-nCoV or nCov or COVID-19 or SARS-CoV-2).mp.
21. or/8-20
22. Triage/
23. exp Resource allocation/
24. Materials Management, Hospital/
25. Eligibility Determination/
26. Guideline/ or Practice Guideline/
27. Decision Making/ or Decision Making, Organizational/
28. Disaster Planning/
29. Health Planning/ or Health Plan Implementation/ or Health Priorities/ or Health Resources/ or Health Planning Guidelines/
30. Clinical Protocols/
31. Medical Ethics/ or Clinical Ethics/
32. Patient Admission/
33. ((health adj2 ration*) or (patient* adj2 (manage* or admi*)) or ((disaster or health) adj2 plan) or decide* or decision* or allocat* or prioritiz* or prioritis* or triag* or ethic* or protocol* or guideline* or criteria).mp
34. or/22-33
35. 7 and 21 and 34

**Supplementary Table 1.** Study characteristics

| **Author**  **(Year published)** | **Continent (Country)** | **Study Design** | **Outbreak** | **Study Population** | **Triage System or Ethical Framework Used or Developed** | **Main Outcome** |
| --- | --- | --- | --- | --- | --- | --- |
| Adalja (2013) | North America (USA) | Conceptual | Influenza pandemic | Patients with influenza and severe ARDS | Murray Score: P/F ratio (FiO_2_ of 1) in mmHg, CXR quadrants infiltrated, PEEP in cm H_2_0, compliance in ml/cm H_2_0 | Using the 2009 H1N1 pandemic experience, a method of cohorting influenza patients with severe ARDS at specific centers has the potential to save lives during a pandemic. However, consensus and refinement with an expert panel and the support of relevant professional societies is needed. |
| Adeniji and Cusack (2011) | Europe (United Kingdom [UK]) | Retrospective cohort | H1N1 | Critically ill adults | Simple Triage Scoring System (STSS): Respiratory rate, shock index, age of at least 75 years, low oxygen saturation; altered mental status, age of 65 to 74 years; SOFA score > 11 | Compared to the SOFA score, STSS accurately risk stratified patients with H1N1 according to their need for ICU admission or mechanical ventilation. The five patients who met the SOFA score >11, all survived. |
| Ardagh (2006) | Oceania (New Zealand) | Text & opinion | Influenza pandemic | NR | Pragmatic guidance for clinicians to prioritize critical care resources: (1) Normal threshold question, (2) Competition question, (3) Alternative options question, (4) Deferability question, (5) Expansion question, (6) Mitigation question, (7) Ranking question, (8) Final question | During an influenza pandemic, clinicians can consider eight questions to make decision about prioritization of access to resources during overwhelming demand |
| Ashton-Cleary (2011) | Europe (UK) | Cross-sectional | H1N1 | Sampled critical care physicians | UK Department of Health (UK DH 10)- adapted Ontario Health Plan for an Influenza Pandemic (OHPIP) criteria: (1) Inclusion criteria, (2) Exclusion criteria (palliative care instead of severe trauma), (3) Minimum qualifications for survival, (4) Prioritization tool. | Nearly 50% of ICU physicians agreed that the original UK Department of Health DH 10 criteria was acceptable for use as a triage tool, with the two most strongly agreed-with criteria: exclude patients with advanced malignant disease or recent major neurological insult. The two least agree-with were: severe trauma and SOFA score > 11. Of the additional criteria, the two most strongly agreed-with: admission follow palliative surgery and patients > 85 years. Two least agreed-with: > 75 years, injured through participation in extreme sports. Replacing "severe trauma" with "palliative surgery" (modified DH 10) improves agreement with the tool from 49 to 68%. |
| Azoulay (2020) | Europe (France) | Expert consultation | COVID-19 | Paris-area healthcare authorities in France | Algorithm for the decision to admit to the ICU | Algorithm to assist physicians in the decision-making process for ICU admission based in ethics and clinical outcomes. |
| Barie (2020) | North America (USA) | Text and opinion | COVID-19 | Executive Order of the Governor of New York State | Tiered system of patient classification:  Tier 1: The most critically ill patients, characterized by any criterion  Tier 2: Any patient with critical illness not classified as Tier 1 or Tier 3 (May be a candidate for transfer to a temporary ICU)  Tier 3: Non-critically ill and ready for transfer to a lower level of care including a temporary ICU (must meet all criteria) | Triage of critically ill patients in New York to authentic ICUs or temporary ICU's (in non-critical care areas) |
| Brandao-Neto (2012) | South America (Brazil) | Prospective cohort | H1N1 | Critically ill patients | SMART-COP; CURB-65; PSI | SMART-COP rule identified most patients within the H1N1 group who required ICU admission. Two other pneumonia severity scores PSI and CURB-65 underestimate severity in pneumonia patients with H1N1 infection. |
| Challen (2007) | North America (USA) | Retrospective cohort | Influenza pandemic | Adult patients (> 15 years old) with pneumonia | Pandemic Modified Warning Score (P-MEWS): Systolic blood pressure, heart rate, respiratory rate, temperature | P-MEWS was a better predictor of need for admission (AUC 0.944) and need of higher level of care (AUC 0.83) compared with CURB-65 (AUCs 0.881 and 0.640, respectively) but was not as good a predictor of subsequent inpatient mortality (AUC 0.663). |
| PCP Ethics Committee (2020) | Unclear | Text and opinion (Ethical guideline) | COVID-19 | PCP Ethics Committee | Guidelines for allocating critical care & allocating ventilators during a situation of extreme shortage | Provider frontline responders a guide in making ethical decisions in management of the emergent COVID-19 crisis. Diminish distress for clinicians when decisions involve conflicts between patient centered care and public health duties |
| Cheung (2012) | Oceania (Australia) | Multicenter retrospective cohort | Influenza pandemic | Critically ill adults | Influenza pandemic ICU triage (iPIT-1) Protocol: (1) Inclusion criteria, (2) Exclusion criteria 1, (3) Exclusion criteria 2, (4) SOFA score, (5) Exclusion criteria 3, (6) Number of organ systems failing, (7) Exclusion criteria 4, (8) D/C criteria 1, (9) D/C criteria 2 | The iPIT-1 protocol resulted in a greater amount of bed availability when applied to patients in post-pandemic influenza, compared to the NSW and OHPIP tools. (*the iPIT protocol has additional limitations on ICU length of stay). |
| Cheung (2012) | Oceania (Australia) | Multicenter retrospective cohort | Influenza pandemic | Critically ill adults | New South Wales (NSW) and OHPIP Pandemic Influenza Triage Protocol: (1) Inclusion criteria, (2) Exclusion criteria (palliative care instead of severe trauma), (3) Minimum qualifications for survival, (4) Prioritization tool | Application of these two triage protocols would increase bed availability in the ICU, with slightly different patterns (OHPIP protocol would have led to a greater increase in bed availability than NSW protocol). |
| Cheung (2017) | Oceania (Australia/New Zealand) | Cross-sectional | Influenza pandemic | Sampled registered voters | Possible triage criteria: (1) Use a first come first served approach to decide, (2) Let a senior doctor decide, (3) Use a set of criteria or rules that have been determined by the Health Department to decide, (4) Random selection, (5) Patient's ability to pay, (6) Use the importance of the patient to decide | The two most preferred triage methods that were also perceived as fair: allowing a senior doctor to decide (43.2%, 95% CI: 37.5-48.8%), triage criteria predetermined by the Health Department (38.7%, 95% CI: 33.1-44.3%). The triage criteria perceived as unfair: "First come, first served," random selection, ability to pay, or person's importance. |
| Christian (2006) | North America (Canada) | Best evidence, expert panels, stakeholder consultation, ethical principles | H5N1 | Steering committee of the OHPIP & critical care leaders | OHPIP criteria: (1) Inclusion criteria, (2) Exclusion criteria (palliative care instead of severe trauma), (3) Minimum qualifications for survival, (4) Prioritization tool | Collaborative process using best evidence, expert panels and key stakeholders includes four main components that provide guidance for making triage decisions during an influenza pandemic. |
| Christian (2009) | North America (Canada) | Retrospective cohort | Influenza pandemic | Critically ill adults | OHPIP criteria: (1) Inclusion criteria, (2) Exclusion criteria (palliative care instead of severe trauma), (3) Minimum qualifications for survival, (4) Prioritization tool | OHPIP triage protocol can help predict patient outcomes (compared to ICU physician's ability without a decision support tool) and make more critical care resources available. However, modification to the triage protocol is needed to: minimize the exclusion of patients who may benefit from critical care and train triage officers (to improve confidence in their decisions and agreement between triage officers and improve equity of the process). |
| Christian (2010) | North America (Canada) | Literature review and expert opinion and Delphi process | Influenza pandemic | European Society of Intensive Care Medicine's Task Force for Intensive Care Unit Triage during an Influenza Epidemic or Mass Disaster | Eight key triage recommendations: (1) Establish and incident management system at all levels of government, (2) Develop fair and equitable policies for ICU services, (3) Acknowledge delivering standard of care may not be possible, (4) ICU treatment and care may need to be withheld, (5) Triage criteria should be objective, ethical, transparent, applied equitably, and publicly available, (6) Triage protocols should be implemented when all critical care resources in an area are strained, (7) Triage of patients for ICU should be based on those who are likely to benefit most or a ‘first come, first served’ basis, (8) Triage officer should apply inclusion and exclusion criteria. | Judicious planning and adoption of critical care triage protocols are necessary during a pandemic. |
| *Christian (2014) | North America (Canada) | Literature review and modified Delphi process | Influenza pandemic | Multidisciplinary Task Force including a total of 100 participants from nine countries | Eleven principles and sub-recommendations on which critical care triage should be based. | A multidisciplinary task force summarized 11 suggestions upon which critical care triage should be based. Critical care triage protocols and the infrastructure, processes, legal protections and training are lacking in most jurisdictions and, as such, should be a last resource. |
| Cinti (2009) | North America (USA) | Literature search, public health information, committee meeting | H1N1 | University of Michigan Health System Scarce Resource Allocation Committee | Guiding Principles for Allocating Antibiotics During a Pandemic | Guiding principles for allocating antibiotics during a pandemic include allocating antibiotics to the sickest patients who have the greatest likelihood for survival. For ICU patients, patients with a SOFA score > 11 should not receive antibiotics (if they are in short supply) and patients with a SOFA score ≤ 11 only when pneumonia is high suspected or proven. |
| Commons and Denholm (2012) | Oceania (Australia) | Prospective Cohort | H1N1 | Patients with pandemic (H1N1) 2009 influenza | Pneumonia severity scores SMRT-CO (systolic blood pressure, multilobar involvement, respiratory rate, tachycardia, confusion, oxygenation), CURB-65 (confusion, urea, respiratory rate, blood pressure, age ≥ 65 years) and PSI (pneumonia severity index)(age, nursing home, coexisting illness, physical examination findings, laboratory and radiographic findings) | The SMRT-CO was the most accurate score (AUC 0.826) for predicting ICU admission. However, available pneumonia severity scores (PSI, CURB-65 and SMRT-CO) has insufficient predictive ability to safely identify low-risk patients with H1N1. |
| Daugherty Biddison (2014) | North America (USA) | Mixed methods | Influenza pandemic | General Public | Ethical framework: (1) First come, first served, (2) Lottery, (3) Prioritize those most likely to survive, (4) Prioritize those with those most years left, (5) Life cycle or "fair innings" principle, (6) Value to others in a pandemic reached on its application. | Public felt prioritizing those likely to survive and value to others in the pandemic were the most acceptable triage criteria. Public discussion about allocation of healthcare resources during an influenza pandemic is important and should include diverse participants to uncover the range of salient issues. The engagement of the public may be politically charged and ethically divisive, but deliberative democracy methods can be used to generate coherent feedback. |
| *Daugherty Biddison (2014) | North America (USA) | Literature search and modified Delphi process | Influenza pandemic | Task Force for Mass Critical Care (clinicians, hospital administrators, public health/government) | Twelve triage/Allocation Suggestions | Consensus of the Task Force includes suggestions for triage and allocation of critical care resources during a mass critical care protocol. However, these suggestions may be controversial and additional research and dialogue is needed. |
| Daugherty Biddison (2018) | North America (USA) | Mixed methods | Influenza pandemic | General public and health-related professionals | Ethical framework: (1) First come, first served, (2) Lottery, (3) Prioritize those most likely to survive, (4) Prioritize those with those most years left, (5) Life cycle or "fair innings" principle, (6) Value to others in a pandemic reached on its application. | Participants indicated prioritizing those most likely to survive and those with the most years left should be considered when making triage decisions. It is important to consult and engage with members of the public when establishing triage criteria and responses to public health disasters. |
| Daugherty Biddison (2019) | North America (USA) | Community Engagement Forums | Influenza pandemic | Forums: Public, healthcare workers, disaster works Expert working group: practitioners, ethicists, public health lawyers, communication specialists Advisory group: intensivist, emergency medicine physician, nurse, two ethicists, two legal experts | Point-based framework for allocation of mechanical ventilation during disasters: (1) Exclusion criteria, (2) Likelihood of short-term survival (SOFA score), (3) Likelihood of long-term survival (comorbidities), (4) Consideration of life stage, (5) Reassess at 24, 48 or 120 hours | Allocation of mechanical ventilation during an influenza pandemic was informed by the values of people of Maryland and is consistent with expert consensus. This must be a living document that is adapted with new research or evolving medical technologies. |
| Devereaux (2008) | North America (USA) | Working group recommendations | Influenza pandemic | Task Force for Mass Critical Care | Task Force suggestions: (1) An equitable triage process utilizing the SOFA scoring system, (2) The concept of triage by a senior clinician(s) without direct clinical obligation and a support system to implement and manage the triage process, (3) Legal and ethical constructs underpinning the allocation of scarce resources, (4) A mechanism for rapid revision of the triage process as further disaster experiences, research, planning and modeling come to light | Triage algorithm should be applied by a triage officer and revised by an expert group at the state or federal level, integrating emerging data once the conditions of the pandemic are better understood. |
| *Dries (2014) | North America (USA) | Literature search and modified Delphi process | Influenza pandemic | Multidisciplinary Task force for Mass Critical Care Special Population | Triage and resource allocation of special populations (e.g., chronically ill, technologically dependent or complex) adhere to the same resource allocation strategy and process as the general population. | The triage and resource allocation of medically fragile and chronically ill patients should adhere to the same resource allocation strategy and process as the general population. Engaging stakeholders and representatives from these special population may lessen the impact of a disaster on these special populations. |
| Eastman (2010) | Europe (UK) | Informed discourse and discussion | H1N1 | St. George's Clinical Ethics Committee, intensivists and specialists in infectious diseases | Triage model: Utilitarian principles within an aggregate public health model (aimed at limiting national influenza mortality and morbidity) with a principle of fairness to maximize consistency and to lessen the psychological impact of decision making upon individuals. | Triage models are advantageous because they suspend usual clinical judgement in favor of a universal triage algorithm, minimizing the psychological stress upon staff making triage decisions. However, due to a possible mismatch between a clinically and acceptable model of triaging based upon a public health approach and the law, health systems should develop a triage model while there is not an imminent health services crisis. |
| Ehmann (2020) | North America (USA) | Text and opinion (Expert Consensus) | COVID-19 | Statewide collaboration amongst five Maryland Health Systems: John Hopkins Medicine, Lifebridge Health, Luminis Health, MedStar Health and University of Maryland Medical System | Operational recommendation for allocation of scarce resources (i.e., mechanical ventilation, ICU resources [beds, equipment, staff], blood components, novel therapies, ECMO and renal replacement therapy) | Each scarce resource varies in nature and underlying data on benefit prevents a single algorithm from being universally applicable. |
| *Einav (2014) | North America (USA) | Literature review and expert opinion using a modified Delphi process | Influenza pandemic | Multidisciplinary Surge Capacity topic panel | Reverse triage**:** Daily evaluation of patients to identify which patients can be discharged safe and ethically (physician discretion) | Triage by an experienced provider, reverse triage, and service de-escalation may all be used to minimize ICU resource consumption. In extreme crises, de-escalation may mean prioritization of less advanced care to a greater number of individuals over delivery of critical care to a few. |
| Emanuel (2020) | North America (USA) | Review and proposed recommendations | COVID-19 | Sounding board, including authors from the Department of Medical Ethics and Health Policy, Perelman School of Medicine, University of Pennsylvania, Philadelphia; the University of Denver Sturm College of Law; the Division of Clinical Public Health, Dalla Lana School of Public Health, University of Toronto, Toronto; and the School of Health Studies, Western University, London, ON; the Preventive Medi-cine Department, Federal University of São Paulo, São Paulo;  the Wellcome Centre of Ethics and Humanities, the Ethox Centre, University of Oxford, Oxford, United Kingdom; and the Department of Emergency Medicine, George Washington University Hospital, Washington, DC. | Ethical values for rationing health resources; Recommendation for who gets health resources during the COVID-19 pandemic | If resources are scarce, recommendations should be applied fairly and consistently across all patients. Guidelines should be tasked to a higher level of authority to alleviate physician burden and ensure ethical treatment. |
| *Enfield (2011)* | North America (USA) | Retrospective cohort | H1N1 | Critically ill adults | APACHE-II; SOFA | Neither the baseline, day three SOFA or APACHE II scores perform sufficiently well to discriminate survivors from non-survivors in pandemic influenza and, as such, should not be used for triage decisions in patients with H1N1. |
| Estella (2012) | Europe (Spain) | Retrospective cohort | H1N1 | Critically ill adults positive for H1N1 | Pneumonia Severity Index (PSI); CURB-65 | PSI and CURB-65 were not good enough predictors of ICU admissions for H1N1 or estimating prognosis and would not helpful in making triage decisions during a pandemic. |
| Farrell (2020) | North America (USA) | Expert consultation | COVID-19 | Older adults | Statement representing the official policy position of the American Geriatrics society | Age criterions included in existing resource allocation strategies disproportionately disfavor older adults (e.g., categorical exclusions based on advanced age). |
| Frolic (2009) | North America (Canada) | Triage protocol development & discussion with stakeholders | Influenza pandemic | Planning meetings included teams around the hospital | Hamilton Health Adult Critical Care Triage and Resource Allocation Protocol for Pandemic Influenza: (1) Build surge capacity, (2) Adjust care provided, (3) Clinical critical care triage using OHPIP triage criteria and triage officers, (4) Supplementary triage criteria, (5) Quality review | The triage protocol addresses gaps in the pandemic literature: human dimensions of triage and the need for specific, ethical, feasible and clinically rigorous guidelines. |
| Grissom (2010) | North America (USA) | Retrospective & prospective cohort | Influenza pandemic | Critically ill adults | SOFA and modified SOFA (MSOFA) scores | The SOFA and MSOFA predict mortality equally well (AUC: 0.83, 95% CI: 0.81-0.95 and 0.84, 95% CI: 0.82-0.85). However, implementation of either the MSOFA or SOFA score as a triage tool during a mass influx of critically ill patients would exclude a significant number of patients who would survive with usual critical care resources. |
| Guest (2009) | Europe (UK) | Prospective | Influenza pandemic | Critically Ill Adults | OHPIP criteria: (1) Inclusion criteria, (2) Exclusion criteria (palliative care instead of severe trauma), (3) Minimum qualifications for survival, (4) Prioritization tool | 69% of patients categorized as too ill to be admitted to ICU survived. This triage tool did not adequately prioritize patients who would benefit from intensive care. |
| Gupta (2020) | North America (Canada) | Text and opinion [Expert opinion (disease-based expert groups)] | COVID-19 | Canadian Thoracic Society (CTS) | Government of Ontario Clinical Triage Protocol with supplemental criteria for patients with underlying chronic respiratory diseases (i.e., cystic fibrosis, pulmonary fibrosis, COPD, PAH) | The current framework assists clinicians with estimating probability of surviving critical illness in patients with underlying chronic respiratory diseases such as cystic fibrosis, pulmonary fibrosis, COPD and pulmonary arterial hypertension |
| Han (2020) | North America (USA) | Expert consultation | COVID-19 | New York State Department of Health Task Force | New York State Ventilatory Allocation Guidelines | Guidelines rely on ethical and clinical evidence-based frameworks, with the aim to save the most lives and can be adapted for other epidemics and pandemics. |
| Herreros (2020) | Europe (Spain) | Expert consultation | COVID-19 | Spanish public institutions and national scientific societies | Triage protocol by clinical criteria for decisions regarding the use of advanced life support | Objective, clinical criteria should be used to guarantee that the ethical principles of equity and greatest food for the greatest number of patients are upheld. |
| Hick (2006) | North America (USA) | Guideline development and review | Pandemic influenza | Multidisciplinary Minnesota Department of Health guideline development group | Tiered, scalable framework for restricting mechanical ventilation | Triage criteria that depend on clinical indicators of survivability and resource utilization to allocate scarce health care resources to those who are most likely to benefit Triage of mechanical ventilation in pandemic situations must be tiered, flexible, and implemented regionally. |
| Ibrahim, et al. 2020 | Asia (United Arab Emirates (UAE)) | Clinical intelligence dashboard design | COVID-19 | Hospital Patients  Dashboard design: United Arab Emirates (UAE) | Clinical intelligence dashboard that uses patient clinical data (e.g., age, comorbidities, laboratory results and clinical parameters) to assign a number that correlates with potential risk of deterioration. | The dashboard allows physicians to efficiently assess the risk of deterioration in a large volume of patients and prioritize clinical care and allocate scarce resources |
| Janig, et al. 2020 | Asia (Afghanistan) | Text and opinion (Consensus) | COVID-19 | Interdisciplinary team of consultants and nurses within the Resolute Support Mission, located at the multinational role 2E, Camp Marmal, Mazar-e Sharif, Afghanistan | Structured Approach for Intensive Care Unit Triage (SAINT) protocol | The triage protocol may be a tool for medical personnel to facilitate the difficult task of triaging. It provides guidance along patient-centered criteria like individual medical, ethical, and legal issues while considering the available resources. Future studies are needed to investigate the effectiveness of the SAINT protocol. |
| Kanter (2015) | North America (USA) | Quantitative computer modelling simulation | Pandemic | Computer model patients; general computer model ICU (not specified) | Pandemic ventilator triage predictor | Performance of unvalidated pandemic ventilator triage predictors may be inferior to first-come, first-served allocation and, as such, fall short of sound ethical foundations. |
| Kaposy (2010) | North America (Canada) | Ethical model adoption | Influenza pandemic | Population of Newfoundland and Labrador - ICU types not specified | The Newfoundland and Labrador Critical Care Triage Protocol (Adapted Hamilton Health Sciences Critical Care Pandemic Triage Protocol) Adjustments: (1) Composition of Triage teams: Eliminating prognosis as a supplementary criterion, (2) Include community impact as a supplementary criterion: Arranging supplementary criteria into two tiers. | The Hamilton Health Sciences Critical Care Pandemic Triage Protocol was amended to include ethics personnel on the triage team or in the decision-making including supplementary criteria (which are social based, not medically based criteria). In addition, supplementary criteria to consider if a death will have a devastating impact on a distinct community or subgroup in the province. |
| Khan (2009) | Europe (UK) | Retrospective cohort | H1N1 | Critically ill adults with H1N1 | SOFA score | SOFA score-based triage would have resulted in withdrawal of treatment in patients who could have survived their ICU stay. |
| Leclerc (2020) | Europe (France) | Expert consultation | COVID-19 | French Society of Anaesthesia and Critical Care, ethics committee, French military medical service-Val-de-Grace | Prioritization for initiation of critical care treatments | Prioritization schemes should consider the level of resource strain and the patient’s wishes, clinical frailty, pre-existing chronic condition and severity and evolution of acute condition. |
| Lin and Anderson-Shaw (2009) | North America (USA) | Literature review, ethical model development and discussion | Influenza pandemic | University of Illinois Medical Center at Chicago Pandemic Influenza Disaster Plan | Ethically based decision-making model for allocation of scarce resources during a pandemic: (1) Formation of a multidisciplinary pandemic triage committee, (2) Phased allocation of resources, (3) Clinical evaluation: Pandemic triage, also must include non-influenza patients, (4) Checklist of clinical progress, (5) Palliative care protocol, (6) Appeals process, (7) Early family involvement | Proposed clinical decision model for clinicians is based on the bioethical principles of beneficence and justice. This model is intended to be used as a template to start discussions and begin planning and preparing for the next pandemic. |
| Marckmann (2020) | Europe (Germany) | Text and opinion (stakeholder consultation) | COVID-19 | Experts from clinical emergency medicine, intensive care medicine, medical ethics, law, and further disciplines were involved in drafting the recommendations | Prioritization based on the patient’s wishes, current illness (SOFA, APACHE-II, CURB-65), general health status (CFS or ECOG) and comorbidities (severe organ dysfunction [heart, lung, liver, kidney], advanced neurological disease, advanced oncological disease, severe irreversible immune deficiency, multimorbidity) | To develop recommendations for the allocation of intensive care resources in the context of the COVID-19 pandemic. The guidance is intended to support responsible decision makers with medically and ethically justified criteria and procedures. These recommendations will be further developed on the basis of new scientific evidence, practical experience and other relevant developments. |
| *Marriott (2012)* | Europe (UK) | Retrospective cohort | H1N1 | Critically ill adults | Critical Care Admission Criteria Admission criteria: SpO_2_<90% on FiO_2_>85%, respiratory acidosis pH ≤7.2, respiratory failure or airway compromise, systolic pressure < 90 mmHg, SOFA score ≥7;  Refusal criteria: SOFA score ≥12, severe trauma, unwitnessed or non-VF arrest, severe life-limiting condition. | Patients who met refusal criteria had prolonged hospital stays or short survival times and, as such, may not represent optimal utilization of resources during increased demand.  A standardized set of admission criteria may supplement decision making during times of increased demand but cannot substitute clinical judgement. |
| *Miller (2010)* | North America (USA) | Retrospective cohort study | H1N1 | Patients who tested positive for H1N1 and spent at least 6 hours in ICU. | SOFA score | Observed mortality was lower than expected when using the SOFA-based triage tool. Eleven patients on Day 1 (who survived) would have been denied care under the current tool's guidelines, if the highest level of triage need was met. |
| *Morton (2014)* | Europe (UK) | Retrospective cohort | H1N1 | Critically ill adults | OHPIP criteria: (1) Inclusion criteria, (2) Exclusion criteria (palliative care instead of severe trauma), (3) Minimum qualifications for survival, (4) Prioritization tool; STSS; Mortality in Emergency Department Sepsis (MEDS) | The STSS score was a better predictor of critical care admission than the OHPIP and MEDS tools. None of the tools were an excellent predictor of admission (i.e., AUC > 0.9). |
| Morton (2015) | Europe (UK) | Retrospective cohort | H1N1 | Patients admitted to hospital within hospital catchment areas and referred to ICU from general wards or EDs | STSS; OHPIP criteria: (1) Inclusion criteria, (2) Exclusion criteria (palliative care instead of severe trauma), (3) Minimum qualifications for survival, (4) Prioritization tool;  Oxygen exchange measured using the PaO_2_/FiO_2_ ratio < 300 | The oxygen exchange ratio (PaO_2_/FiO_2_) was a better predictor for the need for mechanical ventilation (AUC 0.885, 95% CI: 0.817-0.952) and critical care admission (AUC 0.885, 95% CI: 0.804-0.964) compared to the STSS. The reverse triage tool of the OHPIP was a poor predictor of patient outcome and should not be used in patients with H1N1. |
| Montgomery (2020) | Europe (UK) | Text and opinion | COVID-19 | Intensive care society with input from patient and public groups | Intensive Care Society CRITCON-PANDEMIC Ethical Framework & Clinical Decision Aid | The CRITCON-PANDEMIC criteria identifies different phases of a pandemic and the implications for capacity and mutual aid within a national healthcare system. Usual legal and ethical frameworks should continue to apply while capacity and mutual aid are available (CRITCON-PANDEMIC Levels 0-3); clinicians should focus on current clinical needs and should not treat patients differently because of anticipated future pressures. In conditions of resource limitation (CRITCON-PANDEMIC Level 4), a structured and equitable approach is necessary, and an objective Decision Support Aid is proposed. All patients must be treated with respect and without discrimination, because everyone is of equal value. |
| Muller (2010) | North America (Canada) | Prospective cohort | Influenza pandemic (but within context of seasonal influenza) | >15 years old | CURB-65; Morality in Emergency Department Sepsis (MEDS); Nursing Home Acquired Pneumonia in the Elderly (NHAP); Pandemic Medical Early Warning Score (P-MEWS); Pneumonia Severity Index (PSI); Severity Score for the Elderly with Community Acquired Pneumonia (CAP); SMART-COP; Simple Triage Scoring System (STSS) | None of the scores were good predictors of in-hospital mortality or ICU admission. PSI (AUC 0.78, 95% CI: 0.72-0.83) and MEDS (AUC 0.77, 95% CI: 0.71-0.83) scores were fair predictors of death. |
| Murad (2009) | North America (USA) | Cross-sectional | Influenza pandemic | Pediatric and Adult | New York State Department of Health (NYS DOH) guidelines (based on OHPIP and SOFA scores) | NYS DOH Guidelines unsuccessful in triaging ventilators in over half (51.5%) of the patients on mechanical ventilation on the day of the study. |
| Myles (2012) | Europe (UK) | Case control using retrospective data | H1N1 | Pediatric and Adult | Community Assessment Tools (CAT): (1) Severe respiratory distress, (2) Increased respiratory rate, (3) Oxygen saturation ≤92% on pulse oximetry breathing air, or on oxygen, (4) Respiratory exhaustion, (5) Severe dehydration or shock, (6) Altered consciousness level, (7) Causing other clinical concern | CATs best predicted level 2/3 admissions in adults (AUC 0.77, 95% CI: 0.73-  0.80), when compared to CURB-65 (AUC 0.68, 95% CI: 0.64-0.72) and P-MEWS (AUC 0.68, 95% CI: 0.64, 0.73), p<0.001). CURB-65 and CATs were similar in predicting death in adults with both performing better than P-MEWS. CATS potentially useful tool for predicting need for higher levels of care or morality. |
| PCP Ethics Committee (2020) | Unclear | Text and opinion (Ethical guideline) | COVID-19 | PCP Ethics Committee | Allocating critical care:  Criteria based type and severity of the disease, presence of comorbidities, impairment of other organ/systems  and their reversibility  1. Inclusion criteria:  a. Requirement for mechanical ventilator support  b. Hypotension with evidence of shock  2. Exclusion criteria:  a. Low probability of survival  b. Short Expected Life Expectancies  3. Clinical suitability  4. Must not be “first-come, first-served.” basis  trial assessments. | Provider frontline responders a guide in making ethical decisions in management of the emergent COVID-19 crisis. Diminish distress for clinicians when decisions involve conflicts between patient centered care and public health duties |
| Pereira (2012) | 33 different countries from four continents (Africa, Asia, Europe, South America) | Multicenter prospective cohort | H1N1 | Critically Ill adults | Pneumonia Severity Index (PSI); CURB-65; PIRO-CAP | PSI was best predictor of mortality, but still only fair (AUC 0.73, 95% CI, 0.65-0.81). Pneumonia-specific scores undervalued severity and should not be used as instruments to triage-based decisions in the ICU. |
| Powell (2008) | North America (USA) | New York State Workgroup | Public health disaster | New York State Department of Health and New York State Task Force on Life and Law | Ethical and clinical guidelines for allocation of ventilators during an influenza pandemic | A set of draft guidelines was created by the New York State workgroup and will be released for public comment, so final guidelines reflect community values and medical innovation. |
| Prekker (2020) | North America (USA) | Text and opinion | COVID-19 | An existing ECMO workgroup that includes two designees from each of the five regional ECMO centers in Minnesota – generally the ECMO medical director and program coordinator | Framework for prioritizing ECMO resources using a combination of anticipated survival (>60%, 30-60%, <30%), duration of ECMO (<5days, ≥5 days), SOFA score and Age to generate a raw multidimensional strategy score | The framework details different ECMO allocation levels depending on capacity and need during a pandemic. The consortium details what should not be considered when deciding who receives ECMO treatment (e.g., race, gender). A triage team of specialist clinicians and administrators should conjointly apply the provided framework. |
| Real de Asua (2020) | Europe (Spain) | Text and opinion [Ethical guidelines and triage framework development] | COVID-19 | Working Group on triage during the COVID-19 epidemic of the University Hospital of La Princes | Ethical guidelines and triage protocol | The Working Group proposes an algorithmic triage protocol that is supported by ethical principles. |
| Rhodes (2020) | North America (USA) | Text and opinion [Ethical principles] | COVID-19 | 30 emergency care providers from New York City | Ethical resuscitation allocation principles | A framework of nine principles to be considered when deciding who receives resuscitation resources during a pandemic. |
| Rowan (2010) | Europe (UK) | Retrospective cohort | H1N1 | General critical care | Swine Flu Triage (SWIFT): Routine physiological variables recorded during the first 24 hours following ICU admission (lowest systolic blood pressure; highest temperature; highest heart rate; highest respiratory rate; neurological status) and fraction of inspired oxygen (FiO_2_) to produce a score from 0 to 12 points. | Model based on physiological variables and FiO_2_ had only satisfactory concordance, but outperformed CURB-65. Utility of this score during a pandemic is minimal because they are not sufficiently discriminatory to be reliable. |
| Rubinson (2005) | North America (USA) | Literature search, Working Group | Emergency Mass Critical Care | 33 experts from bioethics, biosecurity, critical care, disaster preparedness, government response, hospital administration, hospital engineering and infection control | Working group on emergency mass critical care planning assumptions and recommendations. | The Working Group on Emergency Mass Critical Care offers recommendations on developing triage criteria prior to emergency mass critical care, but these are the beginning of what should be a long-term, strategic effort toward preparations to provide critical care to victims of large-scale disease epidemics. This will require commitments of leaders, practitioners in hospitals, government and the public. |
| Ryan (2020) | North America (USA) | Retrospective cohort | COVID-19 | Critically ill adults | Machine Learning Algorithm (XGBoost ML); qSOFA; P-MEWS; CURB-65 | The XGBoost ML algorithm predicted mortality in all ICU patients as well as mechanically ventilated and pneumonia patients more accurately than qSOFA, MEWS and CURB-65 at all prediction windows. |
| *Saleh (2016)* | Asia (United Arab Emirates) | Prospective cohort | ARDS (in the context of H1N1 and MERS-CoV) | Critically ill adults | APACHE II; APACHE III; SAPS II; SOFA | ARDS is the leading cause of death in critically ill patients during H1N1 and MERS-CoV pandemics. APACHE II/III scoring systems were better at predicting severity and mortality and when compared to SAPS II and SOFA scores. There is a need for an ARDS-specific scoring system for better predictions. |
| Seethala and Keller (2020) | North America (USA) | Text and opinion | COVID-19 | Working group of emergency critical care medicine and pulmonary and critical care medicine specialists | Proposed indications and contraindications for ECMO support during the COVID-19 pandemic | ECMO is resource-intensive therapy that, in the setting of a severe pandemic, requires additional ECMO-specific guidelines for centers to consider during a pandemic |
| Shahpori (2011) | North America (Canada) | Retrospective cohort | H1N1 | General systems ICU | SOFA score > 11 | Hospital mortality in H1N1 patients with an initial SOFA score > 11 was 31% (95%: 5-56%). Applying SOFA threshold of >11 not useful triage criteria. |
| Shekar (2020) | Oceania, Europe, North America | Text and opinion | COVID-19 | Extracorporeal Life Support Organization (ELSO) COVID-19 working group | Ethical guidance for patient selection and discontinuation of ECMO during COVID-19 pandemic | Evidence-based recommendations and best practice guidelines for the provision of ECMO during the ongoing COVID-19 pandemic |
| Silva (2012) | North America (Canada) | Qualitative | Influenza pandemic | Public (defined as resident of Canada who are primarily healthcare system users) | Public-identified criteria that should be considered when making priority setting decisions during a pandemic influenza | Important to involve public in pandemic planning to sustain the public's trust during a public health emergency. |
| Sprung (2010) | Europe (NR) | Literature review, expert opinion, Delphi process | Influenza pandemic | European Society of Intensive Care Medicine Task Force for Intensive Care Unit Triage during an Influenza Epidemic or Mass Disaster | European Society of Intensive Care Medicine Intensive Care Unit Triage during an Influenza Epidemic or Mass Disaster | ICU triage should be based on the "greatest good for the greatest number" of patients and restricted to patients most likely to benefit from ICU care or on a first come, first served basis. Triage criteria should be objective, ethical, transparent, applied equitable and publicly disclosed. All critically ill patients will be assessed by a triage officer will apply inclusion and exclusion criteria and prioritization tool. |
| Sprung (2020) | North America (USA) & Europe | Literature search, guidance documents from professional bodies, internet conference, e-mail | COVID-19 | Experts from Israel, China, UK, USA, Spain, and France | Crisis Level Surge-Critical Care Triage Tool: (1) Inclusion/exclusion criteria, (2) Prioritization for critical care (considering performance score, ASA score, organ failure and predicted survival), (3) Tie-break | A triage algorithm based on clinical estimations of the incremental survival benefit (i.e., saving the most life years) used to allocate strained critical care resources. |
| Steinberg (2020) | Asia (Israel) | Text and opinion | COVID-19 | A joint commission of the Israel National Bioethics Council, the Ethics Bureau of the Israel Medical Association, and the Israeli Ministry of Health appointed by the Director General of the Ministry of Health | Prioritization criteria:  Functional ability (Eastern Cooperative Oncology Group Performance Score), Comorbidities (American Society of Anesthesiologist score, number of organ systems failing (but NOT SOFA), estimation of short-term survival  Tie-breaking: Lives saved>First come first served | Objective, functional clinical criteria and decision-making mechanisms that prohibit discrimination based on race, religion, sex, nationality or citizenship, sexual orientation, socioeconomic status, age, or disability |
| Swiss Society of Intensive Care Medicine (2020) | Europe (Switzerland) | Expert consultation | COVID-19 | The Executive Board of the Swiss Academy of Medical Sciences and the Board of the Swiss Intensive Care Medicine Society | COVID-19 pandemic triage criteria for intensive care treatment under resource scarcity: (1) Inclusion criteria, (2) Exclusion criteria, triage during ICU stay. | If insufficient beds are available, the triage criteria for ICU admission must be uniform, transparently applied by experienced professionals (preferably multidisciplinary) and documented. |
| Tabery and Mackett (2008) | North America (USA) | Ethical framework | Influenza pandemic | University of Pittsburgh Medical Center community and Triage Review Board | Ethical framework for triage during an influenza pandemic | A conceptual framework for anticipating and responding to ethical issues raised by triage in the event of a severe influenza pandemic should be include an institutional triage review board prior to a pandemic. |
| Talmor (2007) | North America (USA) | Retrospective cohort | Influenza pandemic | Emergency departments of two urban tertiary care hospitals | Independent predictors of death: (1) 1. Age of >65 years, (2) Altered mental status, (3) Respiratory rate of >30 breaths/min, (4) Low oxygen saturation, (5) Shock index of >1 (heart rate > blood pressure) | The model had a under the receiver operating characteristic curve when used as predictor for death was 0.73 (ICU admission: 0.70; need for mechanical ventilation: 0.68). The triage rule can serve as an initial guide for fair and just system to provide critical care services in an epidemic. |
| Tambone (2020) | North America, Europe, Africa | Expert opinion | COVID-19 | Specialists from different disciplines, with many in the main epicenters of the COVID-19 pandemic | Ethical triage criteria | Bioethical, clinical, social and legal aspects should be considered when triaging patients in conditions of limited resources. |
| Tillyard (2010) | Europe (UK) | Ethical framework | Influenza pandemic | NR | Ethical framework to ethically maximize an individuals’ best interests | Using an underlying ethical basis and two types of triage (organization and treatment) could maximize the number of patients receiving intensive care based on the patient’s interests. |
| Utley (2011) | Europe (UK) | Mathematical model based on queuing theory | Influenza pandemic | Critically ill patients | Generic mathematical model based on queuing theory | Triage may not result in fewer deaths. It is dependent on the level of demand and the anticipated length of stay and ICU survival between included and excluded groups. |
| Valiani (2020) | North America (Canada) | Text and opinion | COVID-19 | Health authorities throughout Canada developed frameworks for resource allocation to address potentially overwhelming demand for critical care resources because of COVID-19 | Critical Care Triage Stage 4 Protocol that includes:  1. Inclusion criteria (refractory hypoxemia, hypotension)  2. Exclusion criteria (patient preference, past medical history, severity of presenting illness, age >80 ye and Clinical Frailty Scale 5 or greater)  3. Prioritization (SOFA ≤ 7 is highest priority for care)  Tiebreakers: physiologic criteria, life=cycle and instrumental value criteria  4. Reassessment every 72 hours | A staged resource allocation framework to maximize survival to hospital discharge that includes criteria that will not unfairly disadvantage older adults, people who are disabled, and those with chronic health conditions |
| Vergano (2020) | Europe (Italy) | Expert consultation | COVID-19 | SIAARTI Ethic Committee and Executive Council of the scientific society | Criteria for ICU admission and discharge under resource-limited circumstances | The criteria for ICU admission should include principles of distributive justice, clinical appropriateness and proportionality of care (i.e., “the greatest life expectancy”). Modifications to these criteria should be shared widely with other clinicians, patients and their families. |
| Vincent (2020) | Europe (Belgium) | Expert consultation | COVID-19 | Authors from the Department of Intensive Care | Increasingly restrictive triage strategy for ICU admission | Rapid development of triage criteria are important to allocate critical care resources to those who are most likely benefit and survive with a good quality of life. |
| Warrillow (2020) | Oceania (Australia & New Zealand) | Expert opinion | COVID-19 | Senior intensivists from various regions, health care consumers, experts in medical ethics | ANZICS recommended principles for decision making | Guidance to support the consistent, transparent, objective and ethical decision making to support the practice of intensive care specialists during the COVID-19 pandemic. |
| Wilkens and Klein (2010) | North America (USA) | Review of each of the 50 states' public health pandemic and emergency response plans; survey | Influenza pandemic | Critically ill adults | AGILITIES Score and Ventilator Triage Guidelines | AGILITIES Score Ventilator Triage System is based on relative health, duration of time one mechanical ventilation and patients' use of resources during a disaster. It does not require excessive time, lab tests, or additional resources and can assist with allocating limited resources during an influenza pandemic. |
| Williams and Gannon (2009) | Europe (UK) | Prospective cohort | Influenza pandemic | Critically ill patients | SOFA score; OHPIP criteria: (1) Inclusion criteria, (2) Exclusion criteria (palliative care instead of severe trauma), (3) Minimum qualifications for survival, (4) Prioritization tool | Application of SOFA critical could free up nearly one-third more bed days. Patients who met exclusion criteria had longer average length of stays and higher mortality (compared to patients who met inclusion criteria). SOFA score was valid in predicting patient outcomes. SOFA scoring system combined with inclusion and exclusion criteria may be an important adjunct to clinical decision making during an influenza pandemic. |
| Winsor (2014) | North America (Canada) | Task force & cross-sectional | Influenza pandemic | Supplementary Criteria Task Force for Critical Care Triage (critical care physicians, nurses, hospital-based ethicists and researchers affiliated with the Canadian Program of Research on Ethics in a Pandemic) | OHPIP criteria: (1) Inclusion criteria, (2) Exclusion criteria (palliative care instead of severe trauma), (3) Minimum qualifications for survival, (4) Prioritization tool | In addition to the SOFA scoring tool, first come, first served and random selection are the most defensible criterion (out of 13 considered by a wide range of stakeholders). This addresses the gap if two patients are prioritized as high (according to the OHPIP prioritization tool) |

*Part of the CHEST consensus statement

Table sorted by continent (country), then outbreak

*Published abstracts are italicized*

Abbreviations: AGILITIES, Age, Glasgow score, Infusions, Lungs, Interventions, Tests, Informal/incidental, Excessive weight, Subtract; ANZICS, Australian and New Zealand Intensive Care Society; APACHE-II & APACHE III, Acute Physiology and Chronic Health Evaluation II & III; ARDS, Acute Respiratory Distress Syndrome; AUC, Area Under the Curve; CAP, Severity score for the elderly with community acquired pneumonia; CAT, Community Assessment Tools; CI, Confidence Interval; CFS, Chronic Fatigue Syndrome; COPD, Chronic Obstructive Pulmonary Disease; CURB-65, confusion, urea, respiratory rate, blood pressure, age >= 65; DH, Department of Health; ED, Emergency Department; ECMO, Extracorporeal Membrane Oxygenation; ICU, Intensive Care Unit; iPIT-1, Influenza pandemic ICU triage; MEDS, Mortality in Emergency Department Sepsis; MERS-CoV, Middle Eastern Respiratory Syndrome, coronavirus; MEWS, Modified Early Warning System; mSOFA, Modified Sequential Organ Failure Assessment; NHAP, Nursing Home Acquired Pneumonia in the Elderly; NR, Not Reported; NSW, New South Wales; NYS DOH, New York State Department of Health; OHPIP, Ontario Health Plan for an Influenza Pandemic; PAH, Pulmonary Arterial Hypertension; P/F ratio, arterial pO_2_ divided by the FIO_2;_ P-MEWS, Pandemic Modified Warning Score; PIRO-CAP, Predisposition, Insult, Response and Organ dysfunction; PSI, Pneumonia Severity Index; qSOFA, Quick Sequential Organ Failure Assessment; REMS, Rapid Eye Movement; SAPS-II, Simplified Acute Physiology Score; SIAARTI, Italian Society of Anesthesia. Analgesia, Resuscitation and Intensive Care; SMART-COP or SMRT-CO, systolic blood pressure, multilobar involvement, respiratory rate, tachycardia, confusion, oxygenation; SOFA, Sequential Organ Failure Assessment; qSOFA, Quick Sequential Organ Failure Assessment; STSS, Simple Triage Scoring System; SWiFT, Swine Flu Triage; UK, United Kingdom; USA, United States of America

**Supplementary Table 2**. Triage protocols based on an algorithm or point-based method

| **Name (Abbreviation)** | **Description** | **Author (Year published)** |
| --- | --- | --- |
| **Algorithmic** | | |
| Allocation of critical care and ventilators during the COVID-19 pandemic | **ALLOCATING CRITICAL CARE**   1. In a situation off extreme shortage of facilities such as availability of ICU beds, allocation must aim at prioritizing beds to patients with greater chances of therapeutic success. Those who have the “greatest life expectancy” must be given Priority 2. Criteria based type and severity of the disease, presence of comorbidities, impairment of other organ/systems and their reversibility 3. **Inclusion criteria:** 4. Requirement for mechanical ventilator support 5. Hypotension with evidence of shock (altered LOC, decreased urine output, refractory to volume resuscitation and requiring inotropic support). 6. **Exclusion criteria:** 7. Low probability of survival   i. cardiac arrest not responsive to resuscitation  ii. severe irreversible neurologic injury   1. Short Expected Life Expectancies   i. Malignancies with poor prognosis  ii. End stage organ failure with expected survival very short expected survival e.g. severe heart failure, severe COPD and advanced hepatic cirrhosis.   1. **Clinical suitability** 2. **Must not be “first-come, first-served.” Basis**   iii. Allocation of patients to be admitted must be discussed by the medical team in dialogue with the patient/family as applicable  iv. the decision must be made in a timely fashion  v. adapted to the available resources  **ALLOCATING VENTILATORS**   1. The goal is to save the most lives as defined by the patient’s short-term likelihood of surviving the acute medical episode. 2. Triage clinicians should proactively engage in discussions with patients and families regarding do-not-intubate orders for high-risk subgroups of patients before their health deteriorates. Once patients have already been placed on mechanical ventilation, decisions to withdraw it are especially fraught 3. Three Steps in the Allocation Protocol: 4. Application of exclusion criteria:    1. Irreversible shock    2. Unwitnessed arrest, recurrent arrest    3. Trauma related arrest    4. Severe, irreversible neurologic condition at high risk for mortality    5. Patient or patient’s surrogate declines mechanical ventilation    6. Other conditions that may be considered but are highly debatable depending on the status (advanced cirrhosis, dialysis dependent patients, cancer patients with advanced metastasis) 5. Assessment of mortality risk:    1. based on clinical assessment using SOFA Score*    2. Patients who have a moderate risk of mortality and for whom ventilator therapy would most likely be lifesaving are prioritized for treatment    3. Age, social worth and job function shall not affect allocation (high officials, healthcare worker are not specifically prioritized over other patients 6. Periodic clinical assessments (“time trials”)    1. official clinical assessments at 48 and 120 hours after ventilator therapy has begun are conducted to determine whether a patient continues with this treatment.    2. Triage decisions are made based on ongoing clinical measures and data trends of a patient’s health condition, consisting of:       1. the overall prognosis estimated by the patient’s clinical indicators, which is indicative of mortality risk by severity, and number of acute organ failure(s)       2. the magnitude of improvement or deterioration of overall health, which provides additional information about the likelihood of survival with ventilator therapy       3. Guiding principle for the triage decision: The likelihood of a patient’s continuation of ventilator therapy depends on the severity of the patient’s health condition and the extent of the patient’s medical deterioration.    3. In order for a patient to continue with ventilator therapy, s/he must demonstrate an improvement in overall health status at each official clinical assessment.    4. After the 120 hour assessment, patients are evaluated every 48 hours with the same clinical framework used in previous time trial assessments.    5. In addition to removing the responsibility for triage decisions from the bedside clinicians, committee members should also take on the task of communicating the decision to the family. | Committee (2020) |
| Guidelines for ECMO support | **Indications for Venovenous ECMO:**  PaO2:FIO2 ratio < 80 mm Hg for more than 6 hours, despite optimal management listed below:   - Optimized PEEP (Best PEEP trial, esophageal balloon, PV assessment) - Neuromuscular blockade - Prone positioning (spinal cord instability and open chest/abdomen are only contraindications; high BMI is not a contraindication) - Ppl>30 cm H2O on lung protective ventilation - pH<7.15 - No trend towards improvement or other rapidly intervenable pathology (such as pulmonary edema)   **Absolute contraindications:**   - Active solid or liquid malignancy - Age > 65 - High grade shock (norepinephrine dose > 0.2 mcg/kg/min) - Multiorgan failure - Inability to tolerate anticoagulation for initiation of therapy (active hemorrhage) - Invasive mechanical ventilation for longer than 7 days - Irreversible neurologic injury - Expected life expectancy < 6 months   **Relative contraindications:**   - Thrombocytopenia (Platelets < 50,000) - Neutropenia (ANC < 500) - BMI > 40 - Total body weight >180 kg - Long-term chronic respiratory insufficiency treated with oxygen therapy - Unable to perform ADLs at baseline | Seethala and Keller (2020) |
| ECMO provision based on system capacity | **Conventional Capacity (system is running within capacity, judicious ECMO case selection)**   1. *Capacity exists*  - Judicious patient selection - Offer venovenous (V-V), venoarterial (V-A) ECMO in selected COVID-19 patients based on usual criteria - Offer ECMO for non-COVID-19 indications - EPCR only in expert centres   **Contingency Capacity Tier 1 (System is running withing expanded capacity: triage to maximize ECMO capacity to outcome)**  *ii. Expanded capacity*   - Triage to maximize resource: benefit ratio - V-V, V-A ECMO in younger COVID-19 patients with single organ failure - Judicious ECMO use for non-COVID-19 indications - ECPR not offered   **Contingency Capacity Tier 2 (Expanded capacity close to saturation, restrictive ECMO selection criteria)**  *iii. Capacity Saturated*   - Restrictive ECMO criteria for all indications - Prioritize non COVID-19 indications with better chance of survival - V-V ECMO in younger, single organ failure COVID-19 patients - V-A ECMO and ECPR not offered   **Crisis Capacity (System is overwhelmed, ECMO may no longer be appropriate, concentrate resources to usual care)**  *iv. Capacity overwhelmed*   - ECMO not feasible in both COVID-19 and non-COVID-19 patients - Triage ICU admissions - Consider ceasing all futile care to create capacity in the system | Shekar (2020) |
| Saskatchewan Framework for Critical Care Resource Allocation | Critical Care Triage Stage 4 Protocol that includes:   1. **Inclusion criteria:** 2. Refractory hypoxemia  - SpO2, < 90% with FiO2 > 0.85 - Respiratory acidosis (pH < 7.2) - Inability to protect or maintain airway - Clinical evidence of impending respiratory failure  1. Hypotension refractory to volume resuscitation with clinical evidence of shock requiring vasopressor or inotropic therapy  - SBP < 90 mm Hg or relative hypotension PLUS one of: - Altered level of consciousness, decreased urine output, elevated lactate, or other evidence of end-stage organ failure  1. **Exclusion criteria:** 2. Patient preference (ACP or GOC designation) 3. Past medical history  - Metastatic or hematologic malignant disease with an estimated survival < 6 mo - Advanced and irreversible immunocompromise - Severe, irreversible and terminal neurologic event or condition - Advanced untreatable neurodegenerative disease - End stage organ failure not on the transplant list Severity of presenting illness - Cardiac arrest with poor prognostic factors - Severe trauma or burn - Severe neurologic injury  1. Age > 80 yr and Clinical Frailty Scale ≥5 2. **Prioritization (SOFA ≤ 7 is highest priority for care)** 3. Tiebreakers (in order of acceptability): physiologic criteria, lifecycle and instrumental value criteria 4. **Reassessment every 72 hours**  - Consider palliative care if the patient develops 2 additional organ failures - Discharge from ICU: No substantial organ failure - Reassessment every 72 hours | Valiani (2020) |
| Government of Ontario Clinical Triage Protocol with supplemental criteria for patients with cystic fibrosis, pulmonary fibrosis, COPD and pulmonary arterial hypertension (PAH). | Surge level supplemental exclusion criteria for patients with underlying chronic respiratory diseases:  **Level 1-Patients with >80% expected mortality during or in the 6 to 12 months after critical illness**   - *Cystic Fibrosis*: FEV1 <20% predicted when measured at the time of clinical stability - *Pulmonary Fibrosis*: FVC <50%-60%; OR Diffusing capacity of lung for carbon monoxide (DLCO) <30%-40% predicted; OR Chronic supplemental oxygen use at home for >12 hours/day; OR Echocardiographic evidence of pulmonary hypertension (estimated right ventricular systolic pressure >50 mm Hg); OR Rapidly progressive disease; OR History of acute exacerbation of ILD in the last 12 months - *COPD*: FEV1 <50% predicted; AND Chronic hypoxemia (PaO2 ≤ 55 mm Hg) or chronic hypercapnia (PaCO2 > 55 mm Hg); AND Clinical frailty score (CFS) of ≥ 7 - *PAH*: those with a high-risk profile (Registry to Evaluate Early and Long-Term Pulmonary Arterial Hypertension [REVEAL] 2.0 score ≥ 9 or high-risk European Respiratory Society/European Society of Cardiology [ESC/ ERS] score) while on optimal therapy (at least two oral medications and a parenteral prostacyclin, if eligible)   **Level 2-Patients with >50% expected mortality during or in the 6 to 12 months after critical illness**   - Cystic Fibrosis: FEV1 <20% predicted when measured at the time of clinical stability also fall into this category FVC <50%-60%; OR DLCO <30%-40% predicted; OR Chronic supplemental oxygen use at home for >12 hours/day; OR Echocardiographic evidence of pulmonary hypertension (estimated right ventricular systolic pressure >50 mm Hg); OR Rapidly progressive disease (>10% decline in FVC over the last 6 months associated with pronounced radiographic and clinical deterioration); OR History of AE-ILD in the last 12 months - COPD: FEV1 <50% predicted; AND CFS of ≥ 6 - PAH: An intermediate risk profile (REVEAL 2.0 score 7-8 or intermediate-risk ESC/ERS score) while on optimal therapy; AND Age ≥ 75 years; AND Either a recent hospitalization for worsening PAH/right heart failure in the past 3 months or the presence of other significant comorbidities (especially chronic renal failure)   **Level 3-Patients with >30% expected mortality during or in the 6 to 12 months after critical illness**   - Cystic Fibrosis: FEV1 <30% predicted when measured at the time of clinical stability - Pulmonary Fibrosis: FVC <75%; OR DLCO <55% predicted - COPD: FEV1 <50% predicted; AND ≥2 hospitalizations within the last 12 months for an acute exacerbation of COPD; AND CFS of ≥ 5 - PAH: An intermediate-risk profile (REVEAL 2.0 score 7-8 or intermediate-risk ESC/ERS score) while on optimal therapy; AND Age < 75 years AND Either a recent hospitalization for worsening PAH/right heart failure in the past 3 months or the presence of other significant comorbidities (especially chronic renal failure) | Gupta (2020) |
| Structured Approach for Intensive Care Unit Triage (SAINT)-ALGORITHM | If the capabilities are exhausted and there is a realistic chance for successful ICU treatment, patients are prioritized for ICU care (team approach: doctors and nurses) using the following indicators for fatal outcome during initial or re-evaluation:   1. **Indicators for fatal outcome during initial or re-evaluation (current disease)**    1. Current disease    - higher severity (e.g. ARDS)    - Present organ failture/dysfunction (e.g. SOFA Score > 11)    - Signs of acute immune deficiency    - COVID19 risk factors for poor outcome (coagulopathy, cardiac compromise)    - physical fitness prior current disease    1. Comorbidities    - Chronic organ impairment    - COVID19-related comorbidities: hypertension, diabetes mellitus, cardiovascular diseases, lung diseases, active smoker, obesity, other individual factors 2. **Re-evaluate ICU treatment in appropriate periods and in any case if:**  - Relevant clinical changes occur - Capabilities/resources are changing  1. **Requirements for terminating ICU-therapy due to re-evaluation findings:**  - Therapeutic aim cannot be reached realistically - Therapy attempt failed after an adequate period of time and predefined target criteria - Deteriorating organ dysfunction/failure (e.g. increasing SOFA score >2 within 24 hours)  1. **Tiebreaker: operationalized random allocation** | Janig (2020) |
| Allocation of intensive care resources in the context of the COVID-19 pandemic (Clinical and ethical recommendations of  DIVI, DGINA, DGAI, DGIIN, DGNI, DGP, DGP and AEM) | **Intensive care treatment not indicated if:**   - The dying process has started inexorably - The treatment is considered medically inappropriate because no medical improvement or stabilization is expected or - survival can only be achieved by permanent intensive care treatment. - Patient refuses intensive care (e.g., in advance directive, afore orally expressed or substituted judgement)   **When resources scarce, prioritization of patients based on the following criteria using the multiple-eyes principle (two physicians with intensive care experience, one member of nursing staff and, if necessary, representatives from other disciplines [e.g. clinical ethics]:**   1. Patients' current illness (SOFA, APACHE-II, CRB-65) & general health status (prior to current illness) as determined by Clinical Frailty Scale (CFS) or Eastern Cooperative Oncology Group (ECOG). If appropriate, prognostic markers for COVID-19 patients 2. Patient's wishes 3. Comorbidities: severe organ dysfunction (heart, lung, liver, kidney); advanced neurological disease; advanced oncological disease; severe and irreversible immune deficiency; multimorbidity,   **Re-evaluation of intensive care at appropriate intervals, and definitely in the case of:**   - Clinically relevant changes in the clinical prospect of success, as well as changes in the ratio between demand and available resources   **Requirements for withdrawal of intensive care after re-evaluation:**   - Continuation of intensive therapy contradicts (stated or presumed) patient's wishes - Therapeutic goals can no longer be achieved on realistic assumptions - Treatment attempt is unsuccessful after an observation period with preciously defined criteria - Progressive multi-organ failure | Marckmann (2020) |
| Three Tier Classification System for Triage, Communication, and Resource Utilization | **Tier 1:** Most critically ill - requires neuromuscular blockade, P:F <100 on FiO2 greater than equal to > 0.70, PEEP >12 cm H2O, hemodynamic instability (greater than or equal to 2 vasopressors or escalating doses of a single agent), requirement of 1:1 nursing care.  **Tier 2:** Any patient with critical illness not classified as Tier 1 or Tier 3 (May be a candidate for transfer to a temporary ICU) - Mechanical ventilation but no requirement for neuro muscular blockade, P:F> 100 on Fi O2 <0.70, PEEP 10-12cm H20, single vasopressor at a stable or decreasing dose, standard nurse staffing requirement, CRRT permissible provided unit has capacity.  **Tier 3:** Non-critically ill and ready for transfer to a lower level care including a temporary ICU (must meet all criteria) - weaning or liberated from mechanical ventilation with diminished or absent need for sedation - may require non-invasive ventilation, P:F > or equal to 150 on FiO2 <equal to 0.50, PEEP <10 cm H20, normal hemodynamics without need for a vasopressor, intermittent hemodialysis permissible provided unit has capacity | Barie (2020) |
| Maryland operational scarce resource allocation processes | 1. Mechanical Ventilation (Invoked when the supply of ventilators falls to 10% at an individual hospital)    1. *First calculation:*  - Short-term survival (prognosis scores such as SOFA) - Long-term survival (>1 year survival)   1. *Second calculation (for patients on equal footing):* - Pregnancy - Clinical trajectory (priority to improving patients)   1. *Tie-break:* - Random selection (i.e., lottery)  1. ICU Resources (invoked when ICU resource utilization [i.e., beds, equipment, staff] reaches a threshold of 95% capacity)    1. Consensus-based scoring system to allocate ICU resources to patients most needing ICU care including the following weighted factors:  - Urgency of ICU treatment and ICU monitoring - Likelihood of short-term and long-term survival   1. For patients already in the ICU, length of time spent in the ICU and illness severity score trends   2. Low-scoring patients would either not be allocated an ICU bed or, if currently in the ICU, be downgraded to create capacity for a higher scoring patient.   3. No appeal processes   4. At least daily reassessments of eligible patients  1. Blood Components (invoked if "Critical Blood Supply Alert” is triggered)  - Predicted survivability (both short- and long-term) - Ongoing blood needs in relation to current supply  1. ECMO: During ECMO scarcity, barring catastrophic clinical events, a patient placed on ECMO is given a minimum therapeutic trial of seven days before reallocation is considered. A secondary review can be requested for reallocation decisions that would remove a patient from ECMO support. 2. Renal Replacement Therapy: Unique to renal replacement is the ability to conserve resources or to provide less or different dialysis to enhance survival of more patients | Ehmann (2020) |
| Intensive Care Society CRITCON-PANDEMIC Decision Support Aide (Code Red) | **CRITCON-PANDEMIC Levels:**   1. **Level 0 - Normal:** Ability to meet all critical care needs without impact on other services. Normal levels of non-clinical transfer and other "overflow" activity. 2. **Level 1 - Preparatory:** Significant expansion / multiplication of bed capacity, supported by extensive redeployment of staff and equipment from other areas. 3. **Level 2 - Sustained Surge:** System at full stretch, both in ventilator capacity and/or staffing levels, with staff working outside usual role but adherence to usual clinical practice goals wherever possible. Other resources may be becoming limited (e.g., oxygen, renal replacement therapy). 4. **Level 3 - Super Surge**: Some resources starting to be overwhelmed. Full use of stretched staffing ratios and cross-skilling. Delivery of best available care but not usual care, for the majority of patients. 5. **Level 4 - Code Red Triage Risk**: Services overwhelmed, and delivery of critical care is resource limited. This stage should never be reached at any site unless regionally and nationally recognized and declared. Use COVID-19 Decision Support Aid at this stage.    1. **COVID-19 Decision Support Aid:**  - *Age*: Decreasing likelihood of benefit as patient's age increases. - *Clinical Frailty Scale:* Decreasing likelihood of benefit as frailty increases. From most likely to least likely:   - Very Fit - people who are robust, active, energetic and motivated. These people commonly exercise regularly. They are amongst the fittest for their age.   - Well - People who have no active disease symptoms but are less fit than category 1. Often, the exercise of are very active occasionally (e.g., seasonally).   - Managing Well - People whose medical problems are well controlled but are not regularly active beyond walking.   - Vulnerable - While not dependent on others for daily help, often symptoms limit activities. A common complaint is being "slowed up" and being tired in the day.   - Mildly Frail - These people often have more evident slowing, and need help in high order IADLs (finances, transportation, heavy housework, medications). Typically, mild frailty progressively impairs shopping and walking outside alone, meal preparation, and housework.   - Moderately Frail - People who need help with all outside activities and with keeping house. Inside, they often have problems with stairs and need help with bathing and might need minimal assistance (cuing, standby) with dressing.   - Severely Frail - Completely dependent for personal care, from whatever cause (physical or cognitive). Even so, they seem stable and not at high risk of dying (within ~ 6 months).   - Very Severely Frail - Completely dependent, approaching end of life. Typically, they could not recover even from a minor illness.   - Terminally Ill – Approaching the end of life. This category applies to people with a life expectancy of <6 months who are not otherwise evidently frail.   **Co-Morbidity in six months prior: One or more = less likelihood of benefit (list is not ranked and does not imply equal weighting).**   - Cardiac Arrest - from any cause in last 3 years. - Chronic Condition causing:   - ≥ 3 hospital admissions in the last year   - ≥ 4 weeks continuous admission for current inpatients. - Congestive Heart Failure - with symptoms at rest or on minimal exertion. - Chronic Lung Disease - with symptoms at rest or on minimal exertion. - Severe and irreversible neurological condition including moderate to severe dementia. - Chronic Liver Disease with Child-Pugh score ≥ 7. - End Stage Chronic Renal Failure requiring renal replacement therapy. - Immunocompromise - congenital, acquired, or secondary to prescription.   **Using decision support aid and clinical judgement to assess likely outcome:**  Expected to survive, likely to survive, outcome uncertain, not likely to survive, not expected to survive. | Montgomery (2020) |
| University Hospital of La Princes Working Group Triage Guidelines | *Short-term prognosis estimation:*   1. APACHE-II as a decision support tool (initial score and changes over time)   *Long-term prognosis estimation:*   1. Baseline situation: 2. Functional assessment (level of independence in baseline activities of daily living [Barthel index]) and in instrumental activities) 3. Cognitive assessment (e.g., Pfeiffer's test)  - Note: To avoid discrimination against groups with disabilities, consider the presence of functional or cognitive-intellectual dependency, but also its potential progression over time (stable disability processes, such as congenital intellectual disability, compared to processes that involve increasing disability in the future in the short-medium term, such as neurodegenerative processes with progressive cognitive decline)  1. Comorbidities categorized as one of the following: 2. Absence of comorbidities. 3. Comorbidities with little impact on the long-term prognosis and that are not expected to condition the short-term progression of a possible ICU admission (for example, benign prostatic hypertrophy, dyslipidemia). 4. Comorbidities that affect long-term survival or that could complicate the progression of a possible ICU admission (obesity, stable and revascularized ischemic heart disease, chronic kidney disease, COPD) 5. Comorbidities that substantially affect long-term survival (estimated survival < 2 years) 6. Age: Tentative and revisable cut-off point of 80 years of age to unilaterally limit access to advanced life support measures  - Note: this is ethically controversial and was criticized  1. Tie breaker (when two or more patients are reasonably equal: joint assessment between the Intensive Care Medicine teams that will take into consideration the following: 2. Clinical data on the progression from admission 3. Prediction of the expected time of use for the specific resource 4. Consideration of possible transfers and/or the possible use of bridging therapies 5. First come, first served ONLY used as a last resort | Real de Asua (2020) |
| ANZICS (Australian and New Zealand Intensive  Care Society) | 1. Patients who do not require critical care interventions on clinical grounds (due to low illness severity) at the time of assessment should not be admitted to the intensive care unit (ICU). Systems to monitor for clinical deterioration are essential and re-assessment for critical care support should occur if required. 2. ANZICS recommends that all patients (including adults and children with SARS-CoV-2 infection and without it, and whether they are currently being treated in an ICU or not) receive equitable consideration of their needs for intensive care services and equitable access to them. 3. Clinical prioritization should be the initial approach to determine access to intensive care when resources are limited. The approach should incorporate the best information available at the time and be based on a clinical review by senior intensive care clinicians, considering:    1. The likelihood of the patient response to treatment and survival to hospital discharge based on acute illness severity;    2. The severity of patient comorbidities, their independent prognoses and their effect on intensive care outcomes; and    3. The likelihood of long-term patient survival, with an attempt to assess both the quality and the potential quantity of that life. It is acknowledged that the estimation of quality of life following an episode of critical illness may be challenging. 4. The comprehensive patient assessment should include discussions about goals of care, patient and family preferences, and the acceptability to the patient of critical care interventions if offered. The carers and family members of adult patients unable to effectively communicate their preferences may provide valuable insights into their wishes. A competent adult patient may make an informed choice to refuse treatment, and advanced care directives may apply if supported by local legislation and practice. 5. The involvement of two or more senior intensive care clinicians in decision making is recommended where possible and one of these should be the intensivist completing the clinical assessment. Clinicians involved in the previous care of the patient may contribute to the shared decision making where this is possible. Decision makers should have full knowledge of available ICU resources across their own hospital and should engage 6. With relevant jurisdictional coordinators at the time of their assessment to ensure all options have been adequately explored (including the possibility of transfer). In the situation where patients are otherwise similarly ranked in terms of clinical priority, access to intensive care must not be based on irrelevant and discriminatory considerations such as sex, sexual orientation, religion, disability, social status, personal connections, wealth, citizenship, insurance status, ethnicity or race. 7. If a situation arises where patients are similarly ranked in terms of clinical priority, some legitimate instances where it may be ethically justifiable to consider other determinants for prioritization include, but are not exclusive to:    1. supporting patients belonging to groups subjected to social deprivation and disadvantage as a means of redressing their vulnerability;    2. considering that adults with caring responsibilities be prioritized;    3. advocating that younger patients who have lived through fewer life stages are prioritized over older patients; and    4. supporting individuals who undertake front-line patient care and are exposed directly to the risk of infection due to activities inherent to their role (noting that their families also bear some additional risk as a consequence). This recognition reflects their value in maintaining the welfare of patients and is based on the principle of reciprocity. 8. Where it is not possible to achieve consensus on clinical decision making, advice from other relevant experts should be sought (e.g., clinicians from relevant disciplines such as palliative care, aged care, general medicine and respiratory medicine, pediatric care, medical ethicists and chief medical officers). 9. The comprehensive patient assessment, discussions with the patient and family, the process and the clinical prioritization decision, and the clinicians involved in the decision-making process must be clearly documented in the patient’s medical record. 10. Decisions relating to interventions such as extracorporeal membrane oxygenation and other critical care supports should be based on the same process as that used when considering admission to the ICU. It is important to also consider the impact that using resource-intensive interventions has on the ability for an ICU to deliver care to other patients. 11. In some cases, intensive care treatment may not provide the benefit that was hoped for, or a patient may develop complications and it becomes apparent that survival is unlikely. In these difficult circumstances it is then justifiable to consider discontinuation of intensive care therapy in order to provide support to patients who are reasonably expected to benefit. Discontinuation of non-beneficial treatment is accepted as part of normal intensive care practice (especially when therapy may be burdensome to the dying patient) and it is recognized that there is no ethical difference between withholding and withdrawing treatment. 12. It is appropriate to consider earlier discharge of improving patients to general wards when resources are scarce if this creates capacity to admit additional patients in greater need. | Warrillow (2020) |
| Crisis Level Surge-Critical Care Triage Tool | 1. **Assess exclusion & inclusion criteria**   *Exclusion Criteria (patient meets any of the following):*   1. Advanced Directive: not to ventilate, not to admit to ICU, other 2. Patient refuses ICU admission 3. Devastating cerebral injury (e.g., massive intracranial hemorrhage, sever subarachnoid hemorrhage) 4. Metastatic cancer or hematological cancer with poor prognosis   *Inclusion criteria (patient meets any of the following):*   1. Requirement for invasive ventilatory support: [refractory hypoxemia (saturation ≤ 90% on ≥ 60% FiO2), OR respiratory acidosis (pH < 7.2), OR clinical evidence of respiratory failure, OR inability to protect or maintain airway] 2. Requirement for vasopressors/inotropes that cannot be managed on the ward [hypotension (systolic blood pressure <90 mm Hg), with clinical evidence of shock (altered level of consciousness, decreased urine output, or other end organ failure)] 3. **Prioritize for critical care**   *Priority 1:* IF Performance Score (0-1) **AND** ASA score I or II, healthy patient or mild disease **AND** 1 organ failure **AND** predicted survival > 80%  *Priority 2:* IF Performance Score (2) **AND** ASA score II, mild disease **AND** 2-3 organ failures **AND/OR** predicted survival > 50%  *Priority 3:* IF Performance Score (3-4) **AND** ASA score III, severe disease **AND** ≥4 organ failures **AND/OR** predicted survival < 80%  *Priority 4:* S/P cardiac arrest, end stage organ failure (brain, heart, lung, liver, neuro-muscular), trauma/severe burns (est. mortality > 90%), severe dementia, life expectancy (<6 months) **OR** ASA score IV-V, incapacitating disease or moribund **OR** predicted survival <20  **THEN:**   1. Tie-breaking: 2. Allocation by incremental ICU benefit-saving the most life-year 3. If still tie-first come, first served 4. Re-assess priority every 24-hours for patients waiting for ICU admission 5. Re-assess patient at day 10-14 or earlier if significant deterioration | Sprung (2020) |
| Critical Care Admission Criteria | **Admission criteria:** SpO_2_<90% on FiO_2_>85%, respiratory acidosis pH ≤7.2, respiratory failure or airway compromise, systolic pressure < 90 mmHg, SOFA score ≥7  **Refusal criteria:** SOFA score ≥12, severe trauma, unwitnessed or non-VF arrest, severe life-limiting condition | Marriott (2012) |
| European Society of Intensive Care Medicine Task Force for Intensive Care Unit Triage during an Influenza Epidemic or Mass Disaster | **Inclusion criteria for admission to the ICU during a pandemic:** *The patient must have one of the following from either category A or B:* A. Requirement for invasive ventilatory support:   - Refractory hypoxemia (SpO2<90% on non-rebreather mask/FiO_2_>0.85) - Respiratory acidosis with pH<7.2. - Clinical evidence of impending respiratory failure - Inability to protect or maintain airway (altered level of consciousness, significant secretions or another airway issue)   B. Hypotension:   - Hypotension (SBP\90 mmHg or relative hypotension) with clinical evidence of shock (altered level of consciousness, decreased urine output or other end organ failure) refractory to volume resuscitation requiring vasopressor/inotrope support   **Exclusion criteria:**  A. Severe trauma: A Trauma Injury Severity Score (TRISS) with predicted mortality of [80% (see calculator at http://www.sfar.org/scores2/triss2.html) B. Severe burns of patient with any two of the following:   - Age>60 years - >40% of total body surface area affected - Inhalation injury   C. Cardiac arrest   - Unwitnessed cardiac arrest - Witnessed cardiac arrest, not responsive to electrical therapy (defibrillation or pacing) - Recurrent cardiac arrest - A second cardiac arrest less than 72 h following return of spontaneous circulation and stabilization following successful electrical therapy for initial malignant arrhythmia   D. Severe baseline cognitive impairment   - A patient who is unable to perform activities of daily living (AODLs) independently due to cognitive impairment OR is institutionalized due to cognitive impairment   E. Advanced untreatable neuromuscular disease  F. Metastatic malignant disease  G. Advanced and irreversible immunocompromised patient. Most commonly this will be due to AIDS where there are NO antiviral treatment options available or rarely one of the congenital immunocompromised conditions  H. Severe and irreversible neurologic event or condition  I. End-stage organ failure meeting the following criteria:  1. Heart NYHA class III or IV heart failure   - Class I: patients with no limitation of activities; they suffer no symptoms from ordinary activities - Class II: patients with slight, mild limitation of activity; they are comfortable with rest or with mild exertion - Class III: patients with marked limitation of activity; they are comfortable only at rest - Class IV: patients who should be at complete rest, confined to bed or chair; any physical activity brings on discomfort and symptoms occur at rest   2. Lungs   - COPD with FEV1<25% predicted, baseline PaO2<55 mmHg or secondary pulmonary hypertension - Cystic fibrosis with post bronchodilator FEV1<30% or baseline PaO2<55 mmHg - Pulmonary fibrosis with VC or TLC<60% predicted, baseline PaO2<55 mm Hg or secondary pulmonary hypertension - Primary pulmonary hypertension with NYHA class III or IV heart failure, right atrial pressure >10 mmHg or mean pulmonary arterial pressure >50 mmHg - Requirement for home oxygen   3. Liver   - Child-Pugh score≥7 - *Total serum bilirubin*   - 1. Bilirubin<2 mg/dl: 1 point   - 2. Bilirubin 2–3 mg/dl: 2 points   - 3. Bilirubin>3 mg/dl: 3 points - *Serum albumin*   - 1. Albumin>3.5 g/dl: 1 point   - 2. Albumin 2.8–3.5 g/dl: 2 points   - 3. Albumin<2.8 g/dl: 3 points - *INR*   - 1. INR<1.70: 1 point   - 2. INR 1.71 to 2.20: 2 points   - 3. INR>2.20: 3 points - *Ascites*   - 1. No ascites: 1 point   - 2. Ascites controlled medically: 2 points   - 3. Ascites poorly controlled: 3 points - *Encephalopathy*   - 1. No encephalopathy: 1 point   - 2. Encephalopathy controlled medically: 2 points   - 3. Encephalopathy poorly controlled: 3 points   J. Elective palliative surgery - Surgery that is intended for symptomatic relief in a patient with an otherwise terminal condition (i.e., cancer) for which the average 2-year survival is less than 50% K. Patients who are too well  **Prioritization tool:** Blue (Exclusion Criteria of SOFA > 11; medical management +/- palliate and d/c from critical care); Red (SOFA 7 or single organ failure; highest priority); Yellow (SOFA 8-11; Intermediate priority); Green (No significant organ failure; defer or d/c, reassess as needed)   - All patient admitted to ICU should be reassessed at dates 2 and 5 and re-categorized. - Color coded system is also referenced (Christian et al. 2006) | Sprung (2010) |
| Increasingly restrictive triage strategy for ICU admission | **Phase 1:**   - Opening of all ICU beds (when some are usually closed, for example for lack of nurses) - Early discharge of suitable patients to other ward areas (with upgrade in nursing support for these areas if needed/possible) - Transfer of suitable patients to other units, such as the CCU, the recovery room or the stroke unit - Help of additional medical staff (e.g. anaesthetists, pneumologists . . .) if necessary - Maintenance of existing nurse/patient staffing ratios - Non-admission of patients with very poor prognosis (e.g. extensive intracranial bleeding, profound post anoxic coma)   **Phase 2:**  *Add:*   - Expand the numbers of ICU beds – transform the CCU, recovery room or stroke unit into an ICU - Cancel elective surgery - Increase logistic support (help from other floors, reserve-trained ICU nursing/medical staff . . .) - Help from additional nursing staff (coordinated by ICU nursing staff) - Cancel holidays (annual/scientific) for medical and nursing staff - Age limitation (e.g. >85 years), unless very good quality of life - No admission of patients with poor prognosis (e.g. extensive cancer, terminal cardiac or respiratory failure)   **Phase 3:**  *Add:*   - Further recruitment of reserve-trained ICU nursing/medical staff - Additional help from nursing/medical staff from other sectors - Addition of ICU beds in the corridors or other places - Further age limitation (e.g. >80 years), unless very good quality of life - No admission of other patients with a poor prognosis (decompensated cirrhosis, advanced cardiac or respiratory failure) | Vincent (2020) |
| Influenza pandemic intensive care unit triage (iPIT) | **Step 1 - Inclusion:** Pt's admitted to ICU only if they required mechanical ventilation or cardiovascular support with vasopressors or inotropes **Step 2 - Exclusion criteria 1:** If they have elective palliative surgery or severe trauma.  **Step 3 - Exclusion criteria 2:** Acute renal failure; severe burns with 2 of: > 60, covering > 40% of body, inhalation injury; cardiac arrest (unwitnessed, not responding to de-fib, recurring); advanced untreatable neuromuscular disease. **Step 4 - Calculate SOFA score Step 5 - Exclusion Criteria 3:** exclude if SOFA is >=14 OR <=8 ***Step 6 - Calculate number of organ systems failing*** (pulmonary, cardiovascular, renal, liver [bilirubin/ammonia AND/OR cirrhotic], neurological) ***Step 7 - exclusion criteria 4:*** exclude if they have 3 or more from step 6 ***Step 8 - D/C criteria 1:*** Between day 2-6 after admission, d/c if they are no longer receiving mech vent. ***Step 9 - D/C criteria 2:*** On day 7 after admission, d/c patient from ICU.  Continue non-ICU level of care and/or palliative care if indicated. | Cheung (2012) |
| Minnesota Department of Health Guidelines | **Tier 1: Do not offer AND withdraw ventilatory support for patients with any one of the following:**  1. Respiratory failure requiring intubation with persistent hypotension (systolic blood pressure <90 mm Hg for adults) unresponsive to adequate fluid resuscitation after 6–12 hours of therapy and signs of additional end-organ dysfunction (e.g., oliguria, mental status changes, cardiac ischemia) 2. Failure to respond to mechanical ventilation (no improvement in oxygenation or lung compliance) and antibiotics after 72 hours of treatment for a bacterial pathogen (timeline may be modified based on organism-specific data) 3. Laboratory or clinical evidence of R4 organ systems failing   1. Pulmonary (adult respiratory distress syndrome, ventilatory failure, refractory hypoxemia) 2. Cardiovascular (left ventricular dysfunction, hypotension, new ischemia) 3. Renal (hyperkalemia, diminished urine output despite adequate fluid resuscitation, increasing creatinine level) 4. Hepatic (transaminase greater than two times normal upper limit, increasing bilirubin or ammonia levels) 5. Neurologic (altered mental status not related to volume status, metabolic, or hypoxic source, stroke) 6. Hematologic (clinical or laboratory evidence of disseminated intravascular coagulation)   **Tier 2: Do not offer AND withdraw ventilatory support from patients with respiratory failure requiring intubation with the following conditions (in addition to those in tier 1):** Patients with pre-existing system compromise or failure including: 1. Known congestive heart failure with ejection fraction <25% (or persistent ischemia unresponsive to therapy and pulmonary edema) 2. Acute renal failure requiring hemodialysis (related to illness) 3. Severe chronic lung disease including pulmonary fibrosis, cystic fibrosis, obstructive or restrictive diseases requiring continuous home oxygen use before onset of acute illness 4. Acquired immunodeficiency syndrome (AIDS), other immunodeficiency syndromes at stage of disease susceptible to opportunistic pathogens (e.g., CD4 <200 for AIDS) with respiratory failure requiring intubation 5. Active malignancy with poor potential for survival (e.g., metastatic malignancy, pancreatic cancer) 6. Cirrhosis with ascites, history of variceal bleeding, fixed coagulopathy, or encephalopathy 7. Acute hepatic failure with hyperammonemia 8. Irreversible neurologic impairment that makes patient dependent for personal cares (e.g., severe stroke, congenital syndrome, persistent vegetative state)  **Tier 3: Specific protocols to be agreed upon by guideline development committee. Possibilities include:** 1. Restriction of treatment based on disease-specific epidemiology and survival data for patient subgroups (may include age-based criteria) 2. Expansion of pre-existing disease classes that will not be offered ventilatory support 3. Applying Sequential Organ Failure Assessment scoring to the triage process and establishing a cut-off score above which mechanical ventilation will not be offered | Hick (2006) |
| Newfoundland and Labrador Critical Care Triage Protocol | Adapted Hamilton Health Sciences Critical Care Pandemic Triage Protocol  Adjustments made:  **Triage teams:**  Due to the social nature of supplementary criteria, representatives of the ethics service are eligible to be the non-ICU professional member on the triage team (typically an ICU physician, non-physician ICU healthcare provider and professional from outside the ICU). If a member of ethics services is not on the team, they must always be consulted when decision-making includes supplementary criteria. Triage teams should be provided clergy and psychological support.  **Eliminate prognosis as a criterion:**  Prognosis is already built into the SOFA scores included in the triage criteria. In addition, prognosis is subjective criteria and including criteria that requires clinical judgement would give the ICU physician more authority in the decision-making.  **Include community impact as a supplementary criterion:**  Consider if a death will have a devastating impact on a distinct community or subgroup in the province.  **Arranging supplementary criteria into two tiers:**  Tier 1: Multiplier effect, healthcare provider, essential worker and caregiver criteria  Tier 2: Fair innings principle, community impact | Kaposy (2010) |
| New South Wales Influenza pandemic triage protocol (NSW) | **New South Wales Pandemic Influenza Triage Protocol.  Tier 1** Do not offer AND withdraw life-sustaining therapy from patients with any of the following: 1. Resp. failure requiring intubation w. persistent hypotension.  2. Failure to respond to mechanical ventilation and antibiotics after 72 hrs  3. Evidence of >= 4 organ systems failing **Tier 2** Do not offer AND withdraw life-sustaining therapy from patients in respiratory failure requiring intubation with the following, in addition to those in tier 1.  Patients with pre-existing conditions including: 1. Known congestive heart failure  2. Acute renal failure  3. Severe chronic lung disease  4. Immunodeficiency syndromes at stages where patient is susceptible to opportunistic pathogens  5. Acute malignancy w. poor potential for survival  6. Acute hepatic failure.  **Tier 3** Specific triage protocols developed centrally and advised by specialist clinical groups: 1. Restriction of treatment based on disease specific epidemiology and survival data  2. Expansion of pre-existing disease classes that will not be offered ventilation  3. Applying SOFA scoring triage process  **OHPIP Triage Tool:**   - Does patient meet inclusion criteria? - Does patient meet exclusion criteria? - Do initial assessment, 48 hrs assessment, 120 hrs assessment. - Patient is categorized based on SOFA score. Categorized into colours which correspond with an action. - Generally, low SOFA scores will be discharged from ICU   - SOFA 8-11 get immediate priority   - SOFA <=7 get high priority   - SOFA>11 are excluded (too critical; prognosis is poor) - Discharge patients after 12 or 72 hours if they fail to respond to treatment   *(See additional details under OHPIP below)* | Cheung (2012) |
| New York State Department of Health (NYS DOH) guidelines | 1. Exclusion criteria (associated with immediate or near-immediate mortality even with aggressive therapy):  - Cardiac arrest: unwitnessed arrest, recurrent arrest without hemodynamic stability, arrest unresponsive to standard interventions and measures; trauma-related arrest - Irreversible age-specific hypotension unresponsive to fluid resuscitation and vasopressor therapy - Traumatic brain injury with no motor response to painful stimulus (i.e., best motor response = 1) - Severe burns: where predicted survival ≤ 10% even with unlimited aggressive therapy - Any other conditions resulting in immediate or near-immediate mortality even with aggressive therapy*  1. Mortality Risk Assessment Using SOFA:  - *Blue*: Exclusion criteria met or SOFA score > 11-Too ill for ICU support (Action or priority: manage medically, provide palliative care as needed, discharge from critical care) - *Red*: SOFA score ≤ 7 or single-organ failure-Highest priority (Action or priority: admit if bed available) - *Yellow*: SOFA score 8-11-Intermediate priority (Action or priority: admit if no patient in red category requires bed) - *Green*: No significant organ failure (Action or priority: defer or discharge, reassess as needed)  1. Periodic Assessments for Continued Ventilator Use (48-,120-hours) 2. Secondary triage factor: randomization process (i.e., lottery) | Han (2020)  Murad (2009) |
| Ontario Health Plan for an Influenza Pandemic (OHPIP) criteria | **1. Assess whether the patient meets the following inclusion criteria:** The patient must have 1 of the following: A. Requirement for invasive ventilatory support   - Refractory hypoxemia (SpO2 < 90% on non-rebreather mask or FIO_2_ > 0.85) - Respiratory acidosis (pH < 7.2) - Clinical evidence of impending respiratory failure - Inability to protect or maintain airway   B. Hypotension (systolic blood pressure < 90 mm Hg or relative hypotension) with clinical evidence of shock (altered level of consciousness, decreased urine output or other evidence of end-organ failure) refractory to volume resuscitation requiring vasopressor or inotrope support that cannot be managed in ward setting   - *If yes, proceed to step 2* - *If no reassess patient later to determine whether clinical status has deteriorated*   **2. Assess whether the patient meets the following exclusion criteria:**  A. Severe trauma (predicted mortality is > 80% as defined by the Trauma Injury Severity Score or equivalent UK scoring system [the Trauma Audit and Research Network]) B. Severe burns of patient with any two of the following: Age > 60 yr, > 40% of total body surface area affected or inhalation injury C. Cardiac arrest: Unwitnessed cardiac arrest; Witnessed cardiac arrest, not responsive to electrical therapy (defibrillation or pacing); Recurrent cardiac arrest D . Severe baseline cognitive impairment E. Advanced untreatable neuromuscular disease F. Metastatic malignant disease G. Advanced and irreversible immunocompromise H . Severe and irreversible neurologic event or condition I. End-stage organ failure meeting the following criteria: *Heart* (NYHA class III or IV heart failure); *Lungs* (COPD with FEV_1_ < 25% predicted, baseline PaO_2_ < 55 mm Hg, or secondary pulmonary hypertension; Cystic fibrosis with postbronchodilator FEV_1_ < 30% or baseline PaO_2_ < 55 mm Hg; Pulmonary fibrosis with VC or TLC < 60% predicted, baseline PaO_2_ < 55 mm Hg, or secondary pulmonary hypertension; Primary pulmonary hypertension with NYHA class III or IV heart failure, right atrial pressure > 10 mm Hg, or mean pulmonary arterial pressure > 50 mm Hg); *Liver* (Child–Pugh score ≥ 7) J. Age > 85 yr K. Elective palliative surgery   - *If no, proceed to step 3* - *If yes, assign a “blue” triage code; do not transfer the patient to critical care; continue current level of care or provide palliative care as needed*   3. Proceed to triage tool, initial assessment:   - *Blue*: Exclusion criteria met or SOFA score > 11-Too ill for ICU support (Action or priority: manage medically, provide palliative care as needed, discharge from critical care) - *Red:* SOFA score ≤ 7 or single-organ failure-Highest priority (Action or priority: admit if bed available) - *Yellow:* SOFA score 8-11-Intermediate priority (Action or priority: admit if no patient in red category requires bed) - *Green:* No significant organ failure (Action or priority: defer or discharge, reassess as needed) | Christian (2006) Christian (2009) Guest (2009)  Lin and Anderson-Shaw (2009) Morton (2015) Powell (2008) Williams and Gannon (2009) Winsor (2014) |
| OHPIP supplementary criteria (Hamilton Health Adult Critical Care Triage and Resource Allocation Protocol for Pandemic Influenza) | 1. OHPIP criteria completed by triage officers  2. Supplementary triage criteria (when triage decisions cannot be reached after using OHPIP):   1. Clinical evidence of significantly better outcome (prognosis) 2. Multiplier effect (i.e., those with skills and knowledge to save others during the pandemic) 3. Healthcare provide/essential services workplace exposure 4. Caregivers 5. Fair-innings or life-cycle principle   *Deliberation process:*   - Ideally, team has time to reach decisions by consensus - If no consensus can be reached, a fair and transparent lottery process is used | Frolic (2009) |
| Paris-region | Collegiality: the decision remains the responsibility of a single physician, who takes it only after consultation with the healthcare team (the continuity of this collegiality must be organized with at least one other physician and a representative of the non-physician healthcare team)  Respect for the patient’s wishes and values, expressed directly by the patient via advance directives, indirectly by the patient, or reported by the support person, healthcare surrogate, or next of kin   - Attention to the patient’s previous condition, including at least the following: - Frailty assessed using the Clinical Frailty Scale - Age (of particular importance for COVID patients) - Comorbidities (major vs. stabilized, single vs. multiple) - Neurocognitive status (normal, mildly impaired, or severely impaired cognitive functions) - Worsening of the patient’s general condition over the last few months - Attention to current clinical severity, with an assessment of the number of organ failures at the time of decision-making, one of the doctors involved in the latter having examined and spoken with the patient or relatives/support person - Respiratory: hypoxemia (> 6 l/min O2) or respiratory distress - Hemodynamics: systolic blood pressure < 90 mmHg - Neurological: Glasgow Coma Scale score < 12 - Worsening of organ dysfunction - Possible use of the SOFA score - Assessment of the patient’s comfort: pain, anxiety, agitation, dyspnea, congestion, asphyxiation, and isolation - A full commitment to providing support and care for all, in a way that respects the patient’s dignity | Azoulay (2020) |
| Prioritization strategy | **Priority levels:**  P1: Patient will likely not survive without critical care treatments and has a high probability of benefiting from them  *Management decisions:* Initiate critical care treatments or continue them without restriction  P2: Patient will likely not survive without critical care treatments but an intermediate probability of benefiting from them.  *Management decisions:* Initiate critical care treatments or continue them. In similar situations, low availability treatments (such as nitrogen monoxide, NO) should rather be allocated to P1 patients.  P3: Patient does not currently require critical care treatments (or not yet or no longer).  *Management decisions:* Those treatments should be allocated to patients who need them more, and thus have a higher probability to benefit from them. Do not initiate critical care treatment, unless worsening condition prompts reassessment. Discharge from ICU to appropriate downstream unit (respiratory weaning & rehabilitation, medical ward).  P4: Despite critical illness, which might lead to critical care treatments out of resource scarcity, probability of the patient benefiting from them is low. It would be unwise to allocate scarce resources which might be missed by patients with a higher probability of benefiting from critical care treatments.  *Management decisions:* Do not initiate, withhold or withdraw critical care treatments as appropriate, in compliance with good practice and current regulations. Provide at least optimal palliative care in any situation.  **Level of resource scarcity:**   1. Tension:   Criteria to withhold critical care:   - Wish/testimony of kin - Clinical frailty score CFS ≥ 7 (including dementia) - Pre-existing condition: Life expectancy ≤12 months (including cancer); Advanced neuro-degenerative disease; Severe irreversibly neurological condition; Heart failure, NYHA IC; COPD, GOLD 4(D); Liver cirrhosis, Child-Pugh B ≥ 7; Chronic life support (home ventilation, hemodialysis) - Acute condition: Cardiac arrest if unwitnessed, iterative, or not responsive to defibrillation  1. Saturation:   Criteria to withhold critical care (same as level A, plus):   - Clinical frailty score CFS ≥ 5 (including dementia) - Pre-existing condition: Sever stroke sequelae; Heart failure, NYHA III or IV; COPD with pulmonary hypertension or O_2_ at home; Liver cirrhosis Child-Pugh ≥ 6 if ascites or stage ≥ 1 encephalopathy; Chronic disease, KDIGO G5; Age ≥ 85 years; Age ≥ 75 years & 1 sub-criterion or more: Liver cirrhosis (any stage); CKD, KDIGO G3 or above; Heart failure, NYHA II or above; Life expectancy < 24 months - Acute condition: SOFA ≥ 12, or sever organ dysfunctions ≥ 12; Burns, Baux score (=age + TBSA) ≥ 100; Any type of cardiac arrest   Under both scenarios (i.e., tension or saturation):   - SOFA score ≤ 7, or only 1 severe organ dysfunction (P1 critical care) - SOFA score ≥ 8, or at least 2 severe organ dysfunctions (P2 critical care)   Reassess iteratively at least: 48 hours; When level of resource scarcity changes; Upon typical disease turning point:   - Extubated OR PS/tracheostomy (P3 discharge from ICU) - No respiratory stabilization/improvement AND no hemodynamic stabilization/improvement (P4 withhold/withdraw critical care) - See above level A and level B criteria - SOFA score ≥ 12 OR 9 ≤ SOFA score ≤, but no decrease (P4 withdraw critical care, palliative care) - SOFA score < 11 and decreasing (P1 continue critical care) - SOFA score ≤ 8, but no decrease (P2 continue critical care) | Leclerc (2020) |
| SIAARRTI (Italian Society of Anesthesia. Analgesia, Resuscitation and Intensive Care) | 1. Criteria for ICU admission and discharge under exceptional, resource-limited circumstances are flexible and can be locally adapted according to the availability of resources 2. Allocation of ICU resources is a complex and delicate task 3. An age limit for the admission to the ICU may ultimately need to be set. The underlying principle would be to save limited resources which may become extremely scarce for those who have a much greater probability of survival and life expectancy, in order to maximize the benefits for the largest number of people. In the worst-case scenario of complete saturation of ICU resources, keeping a “first come, first served” criterion would ultimately result in withholding ICU care by limiting ICU admission for any subsequently presenting patient. 4. Together with age, the comorbidities and functional status of any critically ill patient presenting in these exceptional circumstances should carefully be evaluated. A longer and, hence, more “resource consuming” clinical course may be anticipated in frail elderly patients with severe comorbidities, as compared to a relatively shorter, and potentially more benign course in healthy young subjects. 5. The presence of advance healthcare directives or advance care planning should be carefully evaluated, especially for patients affected by severe chronic illnesses. These plans should be shared as much as possible between the patient, their proxies and all the healthcare staff involved in patient care. 6. A decision to deny admission to the ICU by applying a “ceiling of care” should always be motivated, communicated and documented. The decision to withhold invasive mechanical ventilation does not necessarily imply that other, non-invasive, modalities of ventilatory support should also be withheld. 7. 7. Under exceptional circumstances, when the availability of resources is overwhelmed by their need, a decision to deny access to one or more life-sustaining therapies, solely based on the principle of distributive justice, may ultimately be justified. 8. A second opinion (e.g. from Regional Healthcare Coordination Centres, or from other recognized or designated experts) may be useful when dealing with particularly difficult or distressing cases. 9. ICU admission criteria should be discussed and defined for each patient as early as possible. Ideally, this would include the creation of a list of patients that should be considered for ICU admission in case of clinical deterioration, given the availability of ICU resources when admission is needed. 10. 0Appropriate palliative care must always be provided to hypoxic patients when a decision to withhold or withdraw life-sustaining treatments is made. Palliative care should be provided according to national or international recommendations, as a matter of good clinical practice. If a prolonged time to death is anticipated, the patient should be transferred to a non-ICU bed; optimal palliative care should be provided also outside the ICU setting. 11. Every admission to the ICU should be considered and communicated as an “ICU trial”. The appropriateness of life-sustaining treatments should be re-evaluated daily, considering the patient’s history, current clinical course, wishes, expected goals and proportionality of ICU care. When a patient is not responding to prolonged life-sustaining treatments, or severe clinical complications arise, a decision to withhold or withdraw further or ongoing therapies should not be postponed in a resource-limited setting during an epidemic. 12. The decision to withhold or withdraw life-sustaining treatments must always be discussed and shared among the healthcare staff, the patients and their proxies, but should also be timely. The daily practice and continuous reassessment of the patients’ clinical course, their wishes, and the availability of resources will strengthen the decision-making process over time. 13. Extracorporeal Membrane Oxygenation (ECMO) is one of the most resource-consuming treatments that can be provided in an ICU setting. As such, it should be reserved for extremely selected patients, for which prompt weaning from extracorporeal support can be anticipated. It should ideally be reserved for high-volume referral centres, where the same procedure could be less resource-consuming than in other, less-experienced, settings. 14. Networking among healthcare professionals is essential to share clinical expertise. Dedicated time and resources should be anticipated for team debriefing and monitoring of burnout symptoms or moral distress among the healthcare staff once time permits. 15. During an epidemic, the impact of restricted visiting policies on families and proxies should be considered, especially when the death of a loved one occurs during times of complete restriction of family visits.   http://www.siaarti.it/SiteAssets/News/COVID19%20-%20documenti%20SIAARTI/SIAARTI%20-%20Covid-19%20-%20Clinical%20Ethics%20Reccomendations.pdf | Vergano (2020) |
| Spanish Triage Criteria | **Baseline clinical situation:**   1. *Ten-year life expectancy, based on age and comorbidities:*   There are several scales and indexes that evaluate the presence and specific weight of comorbidities, such as the modified Charlson Comorbidity Index. Age is directly related to potential life expectancy and should be incorporated as yet another variable in the decision-making process. It is a weighty criterion, but it should not be considered sufficient to make decisions.   1. *Baseline functional and cognitive status:*   The patient’s baseline situation can be assessed through a functional and cognitive assessment of the patients (the most used in our context are the Barthel Index and Pfeiffer test, respectively).  **Current clinical situation:**   1. Illness severity: measured through validated scores such as APACHE II or SOFA, this criterion measures the chances of survival to the current situation depending on the patient’s clinical status at the time of the evaluation. 2. Expected duration of treatment with ALS: 3. Reversibility and recovery potential: predicted recovery and potential negative repercussions that may affect quality of life according to the patient’s perspective. | Herreros (2020) |
| Swiss Academy of Medical Sciences and the Board of the Swiss Intensive Care Medicine Society | **Initial Triage (inclusion criteria):** Does this patient require mechanical ventilation or hemodynamic support with vasoactive agents? **Step 2**: Does the patient have any of the following exclusion criteria: *Stage A:*   - Patient’s wishes (advance directive, etc.) - Unwitnessed cardiac arrest, recurrent cardiac arrest, cardiac arrest with no return of spontaneous circulation - Malignant disease with a life expectancy of less than 12 months - End-stage neurodegenerative disease - Severe and irreversible neurological event or condition - Chronic condition:   - NYHA class IV heart failure; COPD GOLD 4 (D); Liver cirrhosis, Child-Pugh score > 8; Severe dementia; Severe circulatory failure, treatment-resistant despite increased vasoactive dose (hypotension and/or persistent inadequate organ perfusion); Estimated survival <12 months   *Stage B:*   - Severe trauma - Severe burns (>40% of total body surface area affected) with inhalation injury - Severe cerebral deficits after stroke - Chronic condition:   - NYHA class III or IV heart failure; COPD GOLD 4 (D) or COPD A–D with either FEV1 <25% or cor pulmonale or home oxygen therapy (long-term oxygen therapy); Liver cirrhosis with refractory ascites or encephalopathy > stage I; Stage V chronic kidney disease (KDIGO); Moderate dementia (confirmed); Age >85 years; Age >75 years and at least one criterion of liver cirrhosis, stage III chronic kidney disease (KDIGO), NYHA class >I heart failure, or Estimated survival <24 months - *Patients are assessed regularly. If there is no improvement or deterioration, it should be decided whether treatment is to be continued (applies to all COVID-19 and non-COVID-19 patients)* | Swiss Society of Intensive Care Medicine (2020) |
| UK Department of Health (UK DH 10) | Four components from Christian et al. (inclusion criteria, exclusion criteria, minimum qualifications for survival and a prioritization tool)  **UK Department of Health 10 exclusion criteria:** 1. Severe trauma (modified version has "Palliative surgery in context of terminal illness") 2. Severe burns with two from: age>60, >40% surface area or inhalational injury 3. Cardiac arrest: unwitnessed or not responsive to electrical treatment 4. Known severe progressive baseline cognitive impairment 5. Known advanced, untreatable neuromuscular disease 6. Known advanced metastatic malignant disease 7. Known, advanced and irreversible immunocompromise 8. Severe and irreversible neurological event or condition 9. SOFA score > 11 10. End-stage organ failure | Ashton-Cleary (2011) |
| **Point-based** | | |
| Acute Physiology and Chronic Health Evaluation (APACHE-II/APACHE-III) | 1. AaPO_2_ (FiO_2_>50%) or PaO_2_ (FiO_2_<50%): ≥500 or PaO_2_ <55 (4 points), 350-499 or PaO_2_ 55-60 (3 points), 200-349 (2 points), PaO_2_ 61-70 (1 point), <200 or PaO_2_ >70 (0 points) 2. Temperature (rectal): ≤29.9°C or ≥41°C (4 points); 30-31.9°C or 39-40.9°C (3 points), 32-33.9°C (2 points), 34-35.9°C or 38.5-38.9°C (1 point), 36-38.4 (0 points) 3. Mean arterial pressure (mmHg): ≤49 or ≥160 (4 points), 130-159 (3 points), 50-69 or 110-129 (2 points), 70-109 (0 points) 4. Arterial pH or HCO_3_: ≥77; ≤52; <7.15; <15 (4 points), 7.6-7.69;41-51.9; 7.15-7.24; 15-17.9 (3 points), 7.25-7.32; 18-22.9 (2 points), 7.5-7.59; 32-40.9 (1 point), 7.33-7.49; 32-40.9 (0 points) 5. Heart rate: ≤39 or ≥180 (4 points), 40-54 or 140-179 (3 points), 55-69 or 110-139 (2 points), 70-109 (0 points) 6. Respiratory rate: ≤5 or ≥40 (4 points), 35-49 (3 points), 6-9 (2 points), 10-11 or 25-34 (1 point), 12-24 (0 points) 7. Sodium (serum): <111 mEq/L or ≥180 mEq/L (4 points), 111-119 mEq/L or 160-179 mEq/L (3 points), 120-129 mEq/L or 155-159 mEq/L (2 points), 150-154 mEq/L (1 point), 130-149 mEq/L (0 points) 8. Potassium (serum): <2.5 mEq/L or ≥7 mEq/L (4 points), 6.0-6.9 mEq/L (3 points), 2.5-2.9 mEq/L (2 points), 3.0-3.4 mEq/L or 5.5-5.9 mEq/L (1 point), 3.5-5.4 mEq/L (0 points) 9. Serum creatinine (ARF or acute renal failure): >3.5 in ARF (8 points), 2-3.4 in ARF (6 points), 1.5-1.9 in ARF or ≥ 3.5 (4 points), 2-3.4 (3 points), <0.6 or 1.5-1.9 (2 points), 0.6-1.4 (0 points) 10. Hematocrit: <20.0% or ≥60% (4 points), 20.0-29.9% or 50.0-59.9% (2 points), 46.0-49.9% (1 point), 30.0-45.9% (0 points) 11. White blood cell count: <1.0 x10⁹/L or ≥40 x10⁹/L (4 points), 1.0-2.9 x10⁹/L or 20.0-39.9 x10⁹/L (2 points), 15.0-15.9 x10⁹/L ( 1 point), 3.0-14.9 x10⁹/L (0 points) 12. Glasgow Coma Scale: subtract 15 13. Age: ≥75 Years (6 points), 65-74 Years (5 points), 55-64 Years (3 points),45-54 Years (2 points), <45 Years (0 points) | Enfield (2011) Saleh (2016) |
| AGILITIES Score and Ventilatory Triage Guidelines | **AGILITIES Score and Ventilator Triage Guidelines** 1. Medical/Surgical History: Stroke (1 point); Coronary Disease (1 point); Immune Disease (1 point); Heart Surgery (2 points); DM (1 point); COPD (1 point); Asthma (1 point); Drug/EtOH use (1point); Smoking History (1 point); > 10 Home Meds (2 point); Home O_2_ (2 point); CPAP/BiPAP (2 point); Dialysis (4 point) 2. **A**ge in years: 0.5 x (age) 3. **G**lasgow Score: 15 minus GCS Score (best if patient not sedated for 1 hour) 4. **I**nfusions: (Number of infused drugs) x 2 5. **L**ungs: 2 points=use of suction at all in last shift; 3 points=SPO_2_ <88% on 50 % O2; 3 points=PAP >30 cm of H_2_O; 3 points = respiratory rate >30 6. **I**nterventions: 1 point for every line/IC/Drugs started/given in last shift 7. **T**ests: 1 point for every test in past 6 hours 8. **I**nformal/Incidental: 10 points for threat or violent activity to any provider; 3 points for every liter of fluid in last 6 hours 9. **E**xcessive Weight: 3 points=BMI > 30; 6 points = BMI > 40 9. **S**ubtract: 2 points=gestation > 24 weeks Add #1 through #9 and subtract #10 SCORE>100 points=removal from ventilator (automatic) | Wilkens and Klen (2010) |
| Clinical Intelligence Dashboard to optimize critical resources | **Data extracted from EHR:** Facility Identification Number (FIN), Medical Record Number (MRN), Nationality, Sex, Age, Unit Type, Nurse Unit, Room, Bed Number, Registration Date, Arrival Date, Admission Date, ICD-10 Diagnoses Codes, Troponin Result, Troponin Lab Date, Lymphocyte Count, Lymphocyte Lab Date, LDH Result, LDH Lab Date, CRP Result, CRP Lab Date, D-dimer Result, D-dimer Lab Date, Ferritin Result, Ferritin Lab Date, Procalcitonin Result, Procalcitonin Lab Date, ALT Result, ALT Lab Date, Creatinine Result, Creatinine Lab Date, Neutrophil Count, Neutrophil Lab Date, COVID-19 Result, COVID-19 Lab Date, Respiratory Rate, Respiratory Rate Date, Heart Rate, Heart Rate Date, SpO2 Result, SpO2 Result Date, Oxygen Flow Rate, Oxygen Flow Rate Date, Oxygen Therapy, Oxygen Therapy Date, Ventilation Type, Ventilation Type Date, Systolic Blood Pressure, Systolic Blood Pressure Date, Diastolic Blood Pressure, Diastolic Blood Pressure Date, Body Mass Index, Body Mass Index Date, Actual Weight, Actual Weight Date  **Matrix display format:** a row for each patient including pertinent information such as vital signs, oxygen requirements and mode of delivery and a COVID-19 severity score  **Pre-determined algorithm:** each patient is assigned a risk score that correlates with potential risk of deterioration based on the following: age, comorbidities, laboratory results and clinical parameters, patients are assigned a number that correlates with potential risk of deterioration. Each patient is color coded to indicate if their risk score was increasing | Ibrahim (2020) |
| Community Assessment Tools (CAT) | 1. Severe respiratory distress (1 point) 2. Increased respiratory rate (1 point) 3. Oxygen saturation ≤92% on pulse oximetry breathing air, or “on oxygen” (1 point) 4. Respiratory exhaustion (1 point) 5. Evidence of severe clinical dehydration or clinical shock, 6. Altered consciousness level and (1 point) 7. Causing other clinical concern to the patient’s GP or clinical team (1 point) | Myles (2012) |
| Critical care disaster resource allocation framework | Framework (based on a scoring system): *Exclusion criteria:* Cardiac arrest: unwitnessed, recurrent, or unresponsive to defibrillation or pacing; Advanced and irreversible neurologic event or condition; Severe burns in patient with both of the following: Age > 60 years and 50% of total body surface area affected 1. Likelihood of short-term survival based on SOFA (score range 1 to 4)  1 point: SOFA ≤ 8 2 points: SOFA 9-11 3 points: SOFA 12-14 4 points: SOFA > 4  2. Likelihood of long-term survival (score range 0 or 3) Assessment of comorbid conditions (Examples of severe comorbid conditions with associated life expectancy < 1 year: New York Heart Association class IV heart failure; Advanced lung disease FEV1<25% predicted, total lung capacity <60% predicted, or baseline PaO_2_ <55 mm Hg; Primary pulmonary hypertension with New York Heart Association class III or IV heart failure; Chronic liver disease with Child-Pugh Score >7; severe trauma; Advanced untreatable neuromuscular disease; Metastatic malignant disease or high-grade primary brain tumors)  3 points: death likely within 1 year (i.e., any of the above of similar conditions)  Total score of SOFA scores and likelihood of ling term survival: (range 1 to 7)  Priority given to those with the lowest total triage scores. If there is a "tie," life stage considered:   - 1 point: Age 0-49 y - 2 points: Age 50-69 y - 3 points: Age 70-84 y - 4 points: Age ≥ 85 y   Other considerations:   - Pregnant patients with a health fetus will be given a 1-point reduction on their priority score - If patients have the same priority score: first-come, first-served or lottery - Also taking into account response to their response to therapy. If scores increase at 24, 48 or 120 hours, the patient will have an additional point added to their score. Scores that remain unchanged at 120 hours will also have an additional point - Triage officer should be responsible for making decisions. They should be teamed with an experienced critical care nurse, a representative from hospital administration and a nonclinical person (data gathering and documentation) | Daugherty Biddison (2019) |
| Confusion, blood Urea nitrogen, Respiratory rate, Blood pressure, age 65 or older (CURB-65) Score for Pneumonia Severity | *Score composition*   - Confusion (1 point) - BUN > 19 ng/dL (> 7mmol/L) (1 point) - Respiratory Rate ≥ 30 (1 point) - Systolic BP < 90 mmHg or Diastolic BP ≤ 60 mmHg (1 point) - Age ≥ 65 (1 point)   *Score Interpretation:*   - 0 or 1: 1.5% mortality. Outpatient care. - 2: 9.2% mortality. Inpatient vs. observation admission. - 3: 22% mortality. Inpatient admission with consideration for ICU admission with score of 4 or 5. | Challen (2007)  Commons and Denholm (2012) Estella (2012) Muller (2010)  Myles (2012) Pereira (2012) |
| Mortality in Emergency Department Sepsis (MEDS) | - Terminal illness with possible death in 1 month (6 points) - Hypoxia or tachypnea (3 points) - Shock from sepsis (3 points) - Platelet count below 150,000 (3 points) - Granulocytic bands >5% of WBC (3 points) - Patient older than 65 years old (3 points) - Lower respiratory infection (2 points) - Patient is from a nursing home (2 points) - Mental status is altered (2 points) | Morton (2015) Muller (2010) |
| Minnesota ECMO Allocation Framework | **Ethics - Rationing decisions should not consider or be based upon:**  When the demand for ECMO exceeds supply, a regional ECMO triage consultant will assist the Regional Hospital Resource Center in allocating ECMO based on clinical criteria. That is:   - 1. Relative risk for mortality, (regardless of the cause,) and;   2. 2. Relative likelihood and magnitude of clinical benefit.      - When a patient on ECMO is improving or the time frame to improvement is reasonable given the epidemiology of the illness, this patient has priority over other eligible patients. Expected duration of the need for ECMO support may be taken into consideration.      - Age may be taken into consideration as part of constructing prognosis and degree of clinical benefit, but not as an independent consideration distinct from prognosis and degree of clinical benefit.      - Take into consideration best available clinical evidence regarding Covid-19 and the efficacy of ECMO to support survival, per MDH Guidance   3. Alongside significant differences in anticipated clinical benefit derived from ECMO support, the Triage Officer or Team should consider the likely length of need for the scarce resource. Patients who are reliably predicted to need ECMO support for a substantially greater amount of time may be deprioritized to allow more patients to have access.   **Consensus clinical criteria for the prioritization of ECMO by predicted survival and duration are below**.   1. If the demand for ECMO exceeds the supply, then re-allocate ECMO circuits to other patients more likely to benefit clinically when: 2. A patient has been on ECMO for a clinically agreed time-limited trial, and the patient fails to improve or declines.   • It is permissible to re-allocate when a patient’s expected duration of the need is so extreme that it precludes access by many other patients who may benefit with a shorter course.   1. A patient has an underlying condition that makes it relatively certain that ECMO support will not significantly prolong the length of life, even if ECMO achieves its intended short-term medical goal. 2. If the demand for ECMO exceeds the supply, and multiple patients have similar medical needs, prognosis, and likelihood/magnitude of benefit, then apply a fair random procedure to allocate ECMO circuits. 3. If the amount of resources required to maintain patients on ECMO related to personnel and supplies (e.g. circuit disposables, blood products) is not sustainable, then the regional ECMO triage consultant, in consultation with the ECMO medical directors’ group and the Regional Hospital Resource Center, may choose to restrict or discontinue the provision of ECMO. | Prekker (2020) |
| Modified Early Warning Score (MEWS) | **Systolic BP:**   - ≤70 mm Hg (3 points) - 71-80 mm Hg or ≥200 mm Hg (2 points) - 81-100 mm Hg (1 point) - 101-199 mm Hg (0 points)   **Heart rate:**   - ≥130 bpm (3 points) - <40 bpm or 111-129 bpm (2 points) - 41-50 bpm or 101-110 bpm (1 point) - 51-100 bpm (0 points)   **Respiratory rate:**   - >30 bpm (3 points) - <9 bpm or 21-29 bpm (2 points) - 15-20 bpm (1 point) - 9-14 bpm (0 points)   **Temperature:**   - <35°C/95°F or ≥38.5°C/101.3°F (2 points) - 35-38.4°C/95-101.1°F (0 points)   **AVPU Score**   - Alert (0 points) - Reacts to voice (1 point) - Reacts to pain (2 points) - Unresponsive (3 points) | Ryan (2020) |
| Murray Score | **PaO_2_/FiO_2_ ratio (FiO_2_ of 1) in mmHg:**   - >300 (0 points) - 225-299 (1 point) - 175-224 (2 points) - 100-174 (3 points) - <100 (4 points)   **CXR quadrants infiltrated:**   - Normal (0 points) - 1 (1 point) - 2 (2 points) - 3 (3 points) - 4 (4 points)   **PEEP in cm H20:**   - ≤ 5 (0 points) - 6-8 (1 point) - 9-11 (2 points) - 12-14 (3 points) - ≥15 (4 points)   **Compliance in ml/cm H20:**   - ≥80 (0 points) - 60-79 (1 point) - 40-59 (2 points) - 20-39 (3 points) - ≤19 (4 points) | Adalja (2013) |
| Nursing Home Acquired Pneumonia in the Elderly (NHAP) | 1. Respiratory rate >30 breaths/minute (2 points) 2. Pulse >125 beats/minute (1 point) 3. Altered mental status (1 point) 4. History of dementia (1 point) | Muller (2010) |
| Pandemic Modified Warning Score (P-MEWS) | **Systolic BP:**   - ≤70 mm Hg (3 points) - 71-80 mm Hg or ≥200 mm Hg (2 points) - 81-100 mm Hg (1 point) - 101-199 mm Hg (0 points)   **Heart rate:**   - ≥130 bpm (3 points) - <40 bpm or 111-129 bpm (2 points) - 41-50 bpm or 101-110 bpm (1 point) - 51-100 bpm (0 points)   **Respiratory rate:**   - >30 bpm (3 points) - <9 bpm or 21-29 bpm (2 points) - 15-20 bpm (1 point) - 9-14 bpm (0 points)   **Temperature:**   - <35°C/95°F or ≥38.5°C/101.3°F (2 points) - 35-38.4°C/95-101.1°F (0 points) | Challen (2007) Muller (2010)  Myles (2012) |
| PaO_2_(mmHg)/FiO_2_) ratio | P/F ratio <300 as a threshold | Morton (2015) |
| Pneumonia Severity Index (PSI) | **Age:**   - Men (Points in Years) - Women (Points in years - 10)   **Nursing home resident** (+10 points)  **Coexisting illness:**   - Neoplastic disease (+30 points) - Liver disease (+20 points) - Congestive heart failure (+10 points) - Cerebrovascular disease (+10 points) - Chronic renal disease (+10 points)   **Physical-examination findings**   - Acutely altered mental state (+20 points) - Respiratory rate ≥30/min (+20 points) - Systolic blood pressure <90 mm Hg (+20 points) - Temperature <35 or ≥40°C (+15 points) - Pulse ≥125/min (+10 points)   **Laboratory and radiographic findings:**   - Arterial pH < 7.35 (+30 points) - Blood urea nitrogen ≥11 mmol/l (+20 points) - Sodium <130 mmol/l (+20 points) - Glucose ≥14 mmol/l (+10 points) - Haematocrit <30% (+10 points) - PaO_2_ <60 mm Hg or O_2_ saturation ≤90% (+10 points) - Pleural effusion on chest X-ray (+10 points) | Commons and Denholm (2012) Estella (2012) Muller (2010) Pereira (2012) |
| Predisposition, Insult, Response and Organ dysfunction (PIRO-CAP) | **Predisposition:**   - Comorbidities (chronic obstructive pulmonary disease or immunocompromise) (1 point) - >70 yrs (1 point) - Bacteremia (1 point)   **Insult:**   - Multilobar opacities in chest radiograph (1 point)   **Response:**   - Shock (1.1 point) - Severe hypoxemia (1.1 point)   **Organ dysfunction:**   - Acute renal failure or acute respiratory distress syndrome (each 1 point)   *Score range 0–8 points* | Pereira (2012) |
| Quick Sequential Organ Failure Assessment (qSOFA) score | **Altered mental status (GCS <15):**   - Yes (1 point) - No (0 points)   **Respiratory rate ≥22:**   - Yes (1 point) - No (0 point)   **Systolic BP ≤100:**   - Yes (1 point) - No (0 points) | Ryan (2020) |
| Sequential Organ Failure Assessment (SOFA) score | **1. PaO_2_/FiO_2_ mm Hg:**   - > 400 (0 points) - < 400 (1 point) - < 300 (2 points) - < 200 (3 points) - < 100 (4 points)   **2. Platelets, x 10^3^/μL (x 10^6^/L):**   - 150 (> 150) (0 points) - < 150 (< 150) (1 point) - < 100 (< 100) (2 points) - < 50 (< 50) (3 points) - < 20 (< 20) (4 points)   **3. Bilirubin, mg/dL (μmol/L):**   - < 1.2 (< 20) (0 points) - 1.2 - 1.9 (20 - 32) (1 points) - 2.0 - 5.9 (33 - 100) (2 points) - 6.0 - 11.9 (101 - 203) (3 points) - > 12 (> 203) (4 points)   **4. Hypotension:**   - None (0 points) - MABP<70 mm Hg (1 point) - Dop<5 (2 points) - Dop 6 -15 or Epi<0.1 or Norepi<0.1 (3 points) - Dop>15 or Epi>0.1 or Norepi>0.1 (4 points)   **5. Glasgow Coma Scale Score:**   - 15 (0 points) - 13 - 14 (1 point) - 10 - 12 (2 points) - 6 - 9 (3 points) - < 6 (4 points)   **6. Creatinine, mg/dL (μmol/L)**   - < 1.2 (< 106) (0 points) - 1.2 - 1.9 (106 - 168) (1 point) - 2.0 - 3.4 (169 - 300) (2 points) - 3.5 - 4.9 (301 - 433) (3 points) - > 5 (> 434) (4 points)   **Cutoff:** SOFA Score > 11  ***Modified SOFA:*** uses less laboratory values (Respiratory SpO_2_/FiO_2_, Liver appearance/ scans, cardiovascular/hypotension, CNS GCS, renal creatinine) | Adeniji and Cusack (2011) Enfield (2011) Grissom  (2010) Khan (2009)  Miller (2010) Saleh (2016) Shahpori (2011)  Williams and Gannon (2009) |
| Severity Score for the Elderly with Community Acquired Pneumonia (CAP) | 1. Age≥85 years (risk score of 1) 2. Impaired motor response (risk score of 1) 3. Serum creatinine level ≥1.5 mg/dL (risk score of 1) 4. Presence of a comorbid condition (risk score of 2) 5. Abnormal vital sign (risk score of 2) | Muller (2010) |
| Simple Triage Scoring System (STSS) | **Simple Triage Scoring System (STSS):**   - Respiratory rate >30 breaths per minute (4 points) - Shock index >1 (HR>BP) (3 points) - Age of at least 75 years (3 points) - Low oxygen saturation (2 points) - Altered mental status (3 points) - Age of 65 to 74 years (4 points) | Adeniji and Cusack (2011) Morton (2015) Muller (2010)  Talmor (2007) |
| Simplified Acute Physiology Score (SAPS-II) | **Age**   - <40 (0 points) - 40-59 (+7 points) - 60-69 (+12 points) - 70-74 (+15 points) - 75-79 (+16 points) - ≥80 (+18 points)   **Heart Rate**  *If patient had both cardiac arrest (11 points) and extreme tachycardia (7 points), assign 11 points*   - <40 (+11 points) - 40-69 (+2 points) - 70-119 (0 points) - 120-159 (+4 points) - ≥160 (+7 points)   **Systolic Blood Pressure, mmHg**  *Worst value in 24 hours*   - <70 (+13 points) - 70-99 (+5 points) - 100-199 (0 points) - ≥200 (+2 points)   **Temperature ≥39°C (102.2°F)**  *Highest temperature in 24 hours*   - No (0 points) - Yes (+3 points)   **Glasgow Coma Scale**  *Lowest value in 24 hours; if patient is sedated record estimated GCS before sedation*   - 14-15 (0 points) - 11-13 (+5 points) - 9-10 (+7 points) - 6-8 (+13 points) - <6 (+26 points)   **PaO_2_/FiO_2_, if on mechanical ventilation of CPAP**  *Use lowest value in 24 hours; if patient was extubated <24 hours ago, use lowest value while on mechanical ventilation*   - <100 mm Hg/% (13.3 kPa/%) (+11 points) - 100-199 mm Hg/% (13.3-26.5 kPa/%) (+9 points) - ≥200 mm Hg/% (26.6 kPa/%) (+6 points) - Not on mechanical ventilation or CPAP within the last 24 hours (0 points)   **Blood Urea Nitrogen (BUN), mg/dL or serum urea, mmol/L**   - BUN <28 or urea <10 (0 points) - BUN 28-83 or urea 10-29.6 (+6 points) - BUN ≥84 or urea ≥30 (+10 points)   **Urine Output, mL/day**  *If in ICU <24 hours, calculate for 24 hours (e.g. if 1 L in 8 hours then mark 3 L in 24 hours)*   - <500 (+11 points) - 500-999 (+4 points) - ≥1000 (0 points)   **Sodium, mEq/L or mmol/L**  *Worst value in 24 hours*   - <125 (+5 points) - 125-144 (0 points) - ≥145 (+1 point)   **Potassium,** **mEq/L**  *Worst value in 24 hours*   - <3.0 (+3 points) - 3.0-4.9 (0 points) - ≥5.0 (3 points)   **Bicarbonate, mEq/L**  *Lowest value in 24 hours*   - <15 (+6 points) - 15-19 (+3 points) - ≥20 (0 points)   **Bilirubin**  *Highest vale in 24 hours*   - <4.0 mg/dL (<68.4 µmol/L) (0 points) - 4.0-5.9 mg/dL (68.4-102.5 µmol/L) (+4 points) - ≥6.0 mg/dL (≥102.6 µmol/L) (+9 points)   **White Blood Cell, x 10^3^/mm^3^**  *Worst value in 24 hours*   - <1.0 (+12 points) - 1.0-19.9 (0 points) - ≥20.0 (+3 points)   **Chronic diseases**   - None (0 points) - Metastatic cancer (+9 points) - Hematologic malignancy (+10 points) - AIDS (+17 points)   **Type of admission**   - Scheduled surgical = surgery scheduled ≥24 hours prior; medical = no surgery within 1 week of admission; unscheduled surgical = surgery scheduled ≤24 hours prior - Scheduled surgical (0 points) - Medical (+6 points) - Unscheduled surgical (+8 points) | Saleh (2016) |
| SMRT-CO/SMART-COP | **Blood pressure and CXR factors:**   - Systolic BP <90 mmHg (2 points) - Multiple lobes involved on chest x-ray (1 point)   **Respiration/Age factor:**   - Any age: respiratory rate less than 25 breaths/minute (0 points) - 50 years old or younger: respiratory rate 25 breaths/minute or more (1 point) - Over 50 years old: respiratory rate 30 breaths/minute or more (1 point)   **Cardiovascular factor:**   - Tachycardia of 125 beats/minute or more (1 point)   **Mental status factor:**   - Confusion (acute) (1 point)   **Oxygenation/Age factors:**   - Any age: O_2_ Saturation ≥94% (0 points) - 50 years old or younger: O2 Saturation ≤93% (2 points) - Over 50 years old: O_2_ Saturation ≤90% (2 points)   **SMART-COP includes Albumin <3.5 d/dL (1 point) and arterial pH < 7.35 (2 points)* | Commons and Denholm (2012) Muller (2010) |
| Swine Flu Triage (SWIFT) | Routine physiological variables recorded during the first 24 hours following ICU admission (lowest systolic blood pressure; highest temperature; highest heart rate; highest respiratory rate; neurological status) and fraction of inspired oxygen (FiO_2_) to produce a score from 0 to 12 points | Rowan (2010) |
| XGBoost Machine Learning Algorithm | **Clinical variables used**: Age, Heart Rate, Respiratory Rate, Peripheral Oxygen Saturation (SpO2), Temperature, Systolic Blood Pressure, Diastolic Blood Pressure, White Blood Cell Counts, Platelets, Lactate, Creatinine, and Bilirubin, over an interval of 3 h and their corresponding differentials in that interval | Ryan (2020) |

Abbreviations: ACP, Advance Care Planning; ADL/AODL, Activities of Daily Living; AGILITIES, Age, Glasgow score, Infusions, Lungs, Interventions, Tests, Informal/incidental, Excessive weight, Subtract; AIDS, Acquired Immunodeficiency Syndrome; ANC, Absolute Neutrophil Count; ANZICS, Australian and New Zealand Intensive Care Society; APACHE-II & APACHE III, Acute Physiology and Chronic Health Evaluation II & III; ARDS, Acute Respiratory Distress Syndrome; ASA, American Society of Anaesthesiologists; AUC, Area Under the Curve; BiPAP, Bi-Level Positive Airway Pressure; BMI, Body Mass Index; BUN, Blood Urea Nitrogen; CAP, Severity score for the elderly with community acquired pneumonia; CAT, Community Assessment Tools; CCU, Coronary Care Unit; CFS, Clinical Frailty Score; CI, Confidence Interval; CKD, Chronic Kidney Disease; COPD, Chronic Obstructive Pulmownary Disease; CPAP, Continuous Positive Airway Pressure therapy; CRRT, Continuous Renal Replacement Therapy; CURB-65, confusion, urea, respiratory rate, blood pressure, age >= 65; DH, Department of Health; DLCO, Diffusing Capacity of the Lungs for Carbon Monoxide; ECMO, Extracorporeal Membrane Oxygenation; ECOG, Eastern Cooperative Oncology Group; ED, Emergency Department; ERS, European Respiratory Society; ESC, European Society of Cardiology; FEV1, Forced Expiratory Volume, first breath; FVC, Forced Vital Capacity; GOC, Goals of Care; GOLD, Global Initiative for Chronic Obstructive Lung Disease; ICU, Intensive Care Unit; ILD, Interstitial Lung Disease; iPIT-1, Influenza pandemic ICU triage; KDIGO, Kidney Disease: Improving Global Outcomes; MEDS, Mortality in Emergency Department Sepsis; MERS-CoV, Middle Eastern Respiratory Syndrome, coronavirus; MEWS, Modified Early Warning System; mSOFA, Modified Sequential Organ Failure Assessment; NEWS/NEWS2, National Early Warning Score; NHAP, Nursing Home Acquired Pneumonia in the Elderly; NR, Not Reported; NSW, New South Wales; NYHA, New York Heart Association; NYS DOH, New York State Department of Health; OHPIP, Ontario Health Plan for an Influenza Pandemic; P-MEWS, Pandemic Modified Warning Score; P/F ratio, arterial pO_2_ divided by the FIO_2;_ PAH, Pulmonary Arterial Hypertension; PEEP, Positive-end Expiratory Pressure; PIRO-CAP, Predisposition, Insult, Response and Organ dysfunction; PSI, Pneumonia Severity Index; qSOFA, Quick Sequential Organ Failure Assessment; REMS, Rapid Eye Movement; REVEAL, Registry to Evaluate Early and Long-term PAH Disease Management; S/P, Status post (condition after); SAPS-II, Simplified Acute Physiology Score; SBP, Spontaneous Bacterial Peritonitis; SIAARTI, Italian Society of Anesthesia. Analgesia, Resuscitation and Intensive Care; SMART-COP or SMRT-CO, systolic blood pressure, multilobar involvement, respiratory rate, tachycardia, confusion, oxygenation; SOFA, Sequential Organ Failure Assessment; STSS, Simple Triage Scoring System; SWiFT, Swine Flu Triage; TBSA, Total Body Surface Area; TRISS, Trauma Injury Severity Score; UK, United Kingdom; USA, United States of America; WBC, White Blood Cells

**Supplementary Table 3.** Diagnostic accuracies of triage criteria

| **Author (Year)** | **Triage measurement applied** | **Predictive accuracy measurement (95% CI)** | | | | |
| --- | --- | --- | --- | --- | --- | --- |
|  |  | **AUC** | **Sensitivity** | **Specificity** | **PPV** | **NPV** |
| **Algorithmic** | | | | | | |
| Guest (2009) | ***ICU admission*** |  |  |  |  |  |
|  | OHPIP blue (palliative care) | NR | 0.29 (NR) | 0.84 (NR) | NR | NR |
|  | OHPIP green (no significant organ failure) | NR | 0.66 (NR) | 0.83 (NR) | NR | NR |
|  | OHPIP blue (palliative care) - from ED admission data | NR | 0.27 (NR) | 0.87 (NR) | NR | NR |
|  | OHPIP green (no significant organ failure) - from ED admission data | NR | 0.61 (NR) | 0.87 (NR) | NR | NR |
|  | ***Ongoing ICU care at 48 hours after admission*** |  |  |  |  |  |
|  | OHPIP blue (palliative treatment | NR | 0.61 (NR) | 0.87 (NR) | NR | NR |
|  | OHPIP green (no significant organ failure) | NR | 0.76 (NR) | 0.86 (NR) | NR | NR |
|  | OHPIP blue (palliative care) - from ED admission data | NR | 0.63 (NR) | 0.86 (NR) | NR | NR |
|  | OHPIP green (no significant organ failure) - from ED admission data | NR | 0.75 (NR) | 0.86 (NR) | NR | NR |
|  | ***Mortality*** |  |  |  |  |  |
|  | SOFA score at admission | 0.74 (NR) | NR | NR | NR | NR |
|  | SOFA score at ED admission | 0.72 (NR) | NR | NR | NR | NR |
| Miller (2010) | ***Mechanical ventilation*** |  |  |  |  |  |
|  | OHPIP score – “red” and “yellow” | NR | 0.83 (NR) | 0.94 (NR) | NR | NR |
|  | ***ICU admission*** |  |  |  |  |  |
|  | OHPIP score (overall) | NR | 0.76 (NR) | 0.91 (NR) | NR | NR |
|  | OHPIP score (“red” and “orange”) | NR | 0.79 (NR) | 0.99 (NR) | NR | NR |
| Morton (2015) | ***Mechanical ventilation*** |  |  |  |  |  |
|  | OHPIP score (overall) | NR | 0.83 (NR) | 0.94 (NR) |  |  |
|  | ***ICU admission*** |  |  |  |  |  |
|  | OHPIP score (overall) | NR | 0.79 (NR) | 0.99 (NR) |  |  |
| Ryan (2020) | ***Mortality*** |  |  |  |  |  |
|  | XGBoost Machine Learning Algorithm (12-hr window) | 0.86 (NR) | 0.80 (NR) | 0.75 (NR) | NR | NR |
|  | XGBoost Machine Learning Algorithm (24-hr window) | 0.82 (NR) | 0.80 (NR) | 0.67 (NR) | NR | NR |
|  | XGBoost Machine Learning Algorithm (48-hr window) | 0.79 (NR) | 0.80 (NR) | 0.62 (NR) | NR | NR |
|  | XGBoost Machine Learning Algorithm (72-hr window) | 0.75 (NR) | 0.80 (NR) | 0.55 (NR) | NR | NR |
| **Point-based (severity of illness)** | | | | | | |
| Enfield (2011) | ***Hospital mortality at 28 days*** |  |  |  |  |  |
|  | APACHE II score – Day 1 (admission) | 0.73 (NR) | NR | NR | NR | NR |
| Saleh (2016) | ***Mortality*** |  |  |  |  |  |
|  | APACHE-III | After adjustment, significant predictor (Beta = -0.753, p=0.000) of mortality | | | | |
| Adeniji and Cusack (2011) | ***ICU admission*** |  |  |  |  |  |
|  | SOFA score | 0.77 (0.65,0.89) | NR | NR | NR | NR |
|  | ***Mechanical ventilation*** |  |  |  |  |  |
|  | SOFA score | 0.87 (0.72,1.00) | NR | NR | NR | NR |
| Enfield (2011) | ***Hospital mortality at 28 days*** |  |  |  |  |  |
|  | SOFA score – Day 1 (admission) | 0.64 (NR) | NR | NR | NR | NR |
|  | SOFA score – Day 3 | 0.65 (NR) | NR | NR | NR | NR |
| Grissom (2010) | ***Mortality*** |  |  |  |  |  |
|  | SOFA score – Day 1 | 0.83 (0.81,0.85) | NR | NR | NR | NR |
|  | MSOFA score – Day 1 | 0.84 (0.82,0.84) | NR | NR | NR | NR |
|  | SOFA score – Day 3 | 0.78 (0.75,0.81) | NR | NR | NR | NR |
|  | MSOFA score – Day 3 | 0.79 (0.76,0.82) | NR | NR | NR | NR |
|  | SOFA score – Day 5 | 0.72 (0.67,0.76) | NR | NR | NR | NR |
|  | MSOFA score – Day 5 | 0.74 (0.70,0.79) | NR | NR | NR | NR |
|  | SOFA score – medical patients | 0.82 (0.79,0.84) | NR | NR | NR | NR |
|  | MSOFA score – medical patients | 0.82 (0.80,0.85) | NR | NR | NR | NR |
|  | SOFA score – post-operative patients | 0.70 (0.64,0.76) | NR | NR | NR | NR |
|  | MSOFA score – post-operative patients | 0.84 (0.79,0.88) | NR | NR | NR | NR |
|  | SOFA score – trauma patients | 0.87 (0.84,0.90) | NR | NR | NR | NR |
|  | MSOFA score – trauma patients | 0.84 (0.80,0.87) | NR |  | NR | NR |
|  | ***Mechanical ventilation*** |  |  |  |  |  |
|  | SOFA score – Day 1 – MV on Day 3 | 0.83 (0.81,0.84) | NR | NR | NR | NR |
|  | MSOFA score – Day 1 – MV on Day 3 | 0.82 (0.80,0.84) | NR | NR | NR | NR |
|  | SOFA score – Day 1 – MV on Day 5 | 0.76 (0.74,0.78) | NR | NR | NR | NR |
|  | MSOFA score – Day 1 – MV on Day 5 | 0.76 (0.74,0.78) | NR | NR | NR | NR |
| Miller (2010) | ***30 day mortality*** |  |  |  |  |  |
|  | Day 1 SOFA score < 11 | 0.85 (NR) | NR | NR | NR | NR |
|  | Day 3 SOFA score < 11 | 0.88 (NR) | NR | NR | NR | NR |
|  | ***Mechanical ventilation*** |  |  |  |  |  |
|  | SOFA score (overall) | 0.83 (0.75,0.91) | NR | NR | NR | NR |
| Ryan (2020) | ***Mortality*** |  |  |  |  |  |
|  | MEWS (12-hr window) | 0.83 (NR) | 0.90 (NR) | 0.56 (NR) | NR | NR |
|  | MEWS (24-hr window) | 0.80 (NR) | 0.90 (NR) | 0.52 (NR) | NR | NR |
|  | MEWS (48-hr window) | 0.76 (NR) | 0.88 (NR) | 0.44 (NR) | NR | NR |
|  | MEWS (72-hr window) | 0.68 (NR) | 0.87 (NR) | 0.39 (NR) | NR | NR |
|  | qSOFA (12-hr window) | 0.76 (NR) | 0.95 (NR) | 0.37 (NR) | NR | NR |
|  | qSOFA (24-hr window) | 0.74 (NR) | 0.94 (NR) | 0.36 (NR) | NR | NR |
|  | qSOFA (48-hr window) | 0.71 (NR) | 0.95 (NR) | 0.28 (NR) | NR | NR |
|  | qSOFA (72-hr window) | 0.65 (NR) | 0.93 (NR) | 0.25 (NR) | NR | NR |
| Shahpori (2011) | ***Hospital mortality*** |  |  |  |  |  |
|  | SOFA score >11 | Mortality: Case-mix-59 (56, 62); H1N1-31 (5, 56) | | | | |
| **Point-based (epidemic or pandemic)** | | | | | | |
| Myles (2012) | ***Admission to high dependency care or ICU care*** |  |  |  |  |  |
|  | CATs overall | 0.77 (0.73, 0.80) | NR | NR | NR | NR |
|  | CATs overall in H1N1 patients | 0.78 (0.72, 0.83) | NR | NR | NR | NR |
|  | CATs ≥ 3 | 0.68 (0.64,0.72) | 0.49 (0.41,0.56) | 0.87 (0.85,0.89) | 0.44 (0.37,0.51) | 0.89 (0.87,0.91) |
|  | CATs ≥ 4 | 0.60 (0.57,0.63) | 0.22 (0.16,0.29) | 0.98 (0.97,0.99) | 0.74 (0.60,0.85) | 0.86 (0.84,0.88) |
|  | CATs ≥ 5 | 0.52 (0.51,0.54) | 0.04 (0.02,0.09) | 1.00 (0.99,1.00) | 1.00 (0.63,1.00) | 0.84 (0.81,0.86) |
|  | ***Mortality*** |  |  |  |  |  |
|  | CATs overall | 0.70 (0.63, 0.77) | NR | NR | NR | NR |
|  | CATs overall in H1N1 patients | 0.66 (0.56,0.76) | NR | NR | NR | NR |
|  | CATs ≥ 3 | 0.65 (0.58,0.71) | 0.46 (0.34,0.59) | 0.82 (0.80,0.85) | 0.14 (0.10,0.20) | 0.96 (0.94,0.97) |
|  | CATs ≥ 4 | 0.58 (0.53,0.63) | 0.19 (0.10,0.31) | 0.95 (0.94,0.97) | 0.22 (0.12,0.36) | 0.94 (0.93,0.96) |
|  | CATs ≥ 5 | 0.51 (0.49,0.54) | 0.03 (0.00,0.11) | 0.99 (0.98,0.99) | 0.25 (0.03,0.65) | 0.94 (0.92,0.95) |
|  | ***Combined severe outcomes (admission + mortality)*** |  |  |  |  |  |
|  | CATs overall | 0.76 (0.73,0.80) | NR | NR | NR | NR |
|  | CATs overall in H1N1 patients | 0.77 (0.71,0.83) | NR | NR | NR | NR |
|  | CATs ≥ 3 | 0.68 (0.64,0.72) | 0.48 (0.40,0.55) | 0.87 (0.85,0.89) | 0.46 (0.39,0.54) | 0.88 (0.85,0.90) |
|  | CATs ≥ 4 | 0.60 (0.57,0.63) | 0.21 (0.15,0.28) | 0.98 (0.97,0.99) | 0.77 (0.63,0.87) | 0.84 (0.82,0.87) |
|  | CATs ≥ 5 | 0.52 (0.51,0.54) | 0.04 (0.01,0.08) | 1.00 (0.99,1.00) | 1.00 (0.63,1.00) | 0.82 (0.79,0.84) |
| Challen (2007) | ***In hospital mortality*** |  |  |  |  |  |
|  | PMEWS | 0.66 (0.57,0.75) | NR | NR | NR | NR |
|  | ***ICU admission*** |  |  |  |  |  |
|  | PMEWS > 1 | NR | 98 (NR) | 55 (NR) | 82 (NR) | 95 (NR) |
|  | PMEWS > 2 | NR | 93 (NR) | 71 (NR) | 87 (NR) | 84 (NR) |
|  | PMEWS > 3 | NR | 86 (NR) | 87 (NR) | 93 (NR) | 75 (NR) |
|  | PMEWS > 4 | NR | 76 (NR) | 94 (NR) | 96 (NR) | 65 (NR) |
|  | PMEWS > 5 | NR | 64 (NR) | 98 (NR) | 99 (NR) | 56 (NR) |
|  | PMEWS > 7 | NR | 40 (NR) | 100 (NR) | 100 (NR) | 44 (NR) |
|  | PMEWS > 9 | NR | 21 (NR) | 100 (NR) | 100 (NR) | 37 (NR) |
|  | PMEWS > 11 | NR | 8 (NR) | 100 (NR) | 100 (NR) | 34 (NR) |
| Muller (2010) | ***In hospital mortality*** |  |  |  |  |  |
|  | PMEWS | 0.68 (0.61,0.74) | NR | NR | NR | NR |
|  | ***Low risk of death*** |  |  |  |  |  |
|  | PMEWS | NR | 0.97 (NR) | 0.13 (NR) | NR | NR |
|  | ***ICU admission*** |  |  |  |  |  |
|  | PMEWS | 0.63 (0.57,0.69) | NR | NR | NR | NR |
|  | ***ICU admission with fatal cases not admitted to ICU excluded*** |  |  |  |  |  |
|  | PMEWS | 0.64 (0.58,0.70) | NR | NR | NR | NR |
| Myles (2012) | ***Admission to high dependency care or ICU care*** |  |  |  |  |  |
|  | PMEWS overall | 0.68 (0.64, 0.73) | NR | NR | NR | NR |
|  | PMEWS overall in H1N1 patients | 0.70 (0.60,0.76) | NR | NR | NR | NR |
|  | PMEWS > 1 | 0.52 (0.51,0.53) | 0.98 (0.94,0.99) | 0.63 (0.47,0.81) | 0.18 (0.15,0.20) | 0.93 (0.83,0.98) |
|  | PMEWS > 2 | 0.54 (0.51,0.56) | 0.90 (0.85,0.94) | 0.17 (0.15,0.20) | 0.18 (0.16,0.21) | 0.90 (0.84,0.94) |
|  | PMEWS > 3 | 0.54 (0.51,0.56) | 0.90 (0.85,0.94) | 0.17 (0.15,0.20) | 0.18 (0.16,0.21) | 0.90 (0.84,0.94) |
|  | PMEWS > 4 | 0.59 (0.56,0.62) | 0.85 (0.79,0.90) | 0.33 (0.29,0.36) | 0.20 (0.18,0.24) | 0.91 (0.87,0.94) |
|  | PMEWS > 5 | 0.62 (0.58,0.65) | 0.76 (0.69,0.82) | 0.47 (0.44,0.50) | 0.23 (0.19,0.26) | 0.91 (0.87,0.93) |
|  | PMEWS > 7 | 0.65 (0.61,0.69) | 0.55 (0.48,0.63) | 0.75 (0.72,0.78) | 0.31 (0.26,0.36) | 0.89 (0.87,0.91) |
|  | PMEWS > 9 | 0.57 (0.54,0.61) | 0.25 (0.19,0.32) | 0.90 (0.87 0.92) | 0.33 (0.25,0.42) | 0.85 (0.83,0.88) |
|  | PMEW > 11 | 0.52 (0.50,0.54) | 0.05 (0.02,0.09) | 0.99 (0.98,0.99) | 0.45 (0.23,0.68) | 0.83 (0.81,0.86) |
|  | ***Mortality*** |  |  |  |  |  |
|  | PMEWS overall | 0.60 (0.52, 0.67) | NR | NR | NR | NR |
|  | PMEWS overall in H1N1 patients | 0.58 (0.46,0.69) | NR | NR | NR | NR |
|  | PMEWS > 1 | 0.52 (0.50,0.54) | 0.98 (0.91,1.00) | 0.05 (0.04,0.07) | 0.06 (0.04,0.07) | 0.98 (0.90,1.00) |
|  | PMEWS > 2 | 0.52 (0.47,0.56) | 0.87 (0.76,0.94) | 0.16 (0.14,0.18) | 0.06 (0.04,0.08) | 0.95 (0.90,0.97) |
|  | PMEWS > 3 | 0.52 (0.47,0.56) | 0.87 (0.76,0.94) | 0.16 (0.14,0.18) | 0.06 (0.04,0.08) | 0.95 (0.90,0.97) |
|  | PMEWS > 4 | 0.54 (0.48,0.59) | 0.77 (0.65,0.87) | 0.30 (0.27,0.33) | 0.06 (0.04,0.08) | 0.95 (0.92,0.97) |
|  | PMEWS > 5 | 0.55 (0.49,0.61) | 0.66 (0.53,0.77) | 0.43 (0.40,0.46) | 0.06 (0.05,0.09) | 0.95 (0.92,0.97) |
|  | PMEWS > 7 | 0.59 (0.52,0.65) | 0.46 (0.34,0.59) | 0.70 (0.67,0.73) | 0.09 (0.06,0.13) | 0.95 (0.93,0.96) |
|  | PMEWS > 9 | 0.54 (0.49,0.60) | 0.21 (0.11,0.33) | 0.87 (0.85,0.89) | 0.09 (0.05,0.16) | 0.94 (0.92,0.96) |
|  | PMEWS > 11 | 0.51 (0.48,0.53) | 0.03 (0.00,0.11) | 0.98 (0.97,0.98) | 0.10 (0.01,0.31) | 0.94 (0.92,0.95) |
|  | ***Combined severe outcomes (admission + mortality)*** |  |  |  |  |  |
|  | PMEWS overall | 0.67 (0.63,0.71) | NR | NR | NR | NR |
|  | PMEWS overall in H1N1 patients | 0.68 (0.61,0.74) | NR | NR | NR | NR |
|  | PMEWS > 1 | 0.52 (0.51,0.53) | 0.97 (0.94,0.99) | 0.06 (0.04,0.08) | 0.19 (0.16,0.21) | 0.93 (0.83,0.98) |
|  | PMEWS > 2 | 0.53 (0.51,0.56) | 0.89 (0.84,0.93) | 0.17 (0.14,0.20) | 0.19 (0.17,0.22) | 0.88 (0.82,0.95) |
|  | PMEWS > 3 | 0.53 (0.51,0.56) | 0.89 (0.84,0.93) | 0.17 (0.14,0.20) | 0.19 (0.17,0.22) | 0.88 (0.82,0.92) |
|  | PMEWS > 4 | 0.58 (0.55,0.61) | 0.83 (0.77,0.88) | 0.32 (0.29,0.35) | 0.21 (0.18,0.24) | 0.89 (0.85,0.92) |
|  | PMEWS > 5 | 0.61 (0.57,0.65) | 0.74 (0.68,0.80) | 0.47 (0.43,0.50) | 0.24 (0.20,0.27) | 0.89 (0.86,0.92) |
|  | PMEWS > 7 | 0.64 (0.60,0.68) | 0.53 (0.46,0.60) | 0.74 (0.71,0.77) | 0.32 (0.27,0.37) | 0.87 (0.85,0.90) |
|  | PMEWS > 9 | 0.57 (0.53,0.60) | 0.23 (0.17,0.30) | 0.89 (0.87,0.91) | 0.33 (0.25,0.42) | 0.83 (0.81,0.86) |
|  | PMEWS > 11 | 0.52 (0.50,0.54) | 0.05 (0.02,0.09) | 0.98 (0.97,0.99) | 0.50 (0.27,0.72) | 0.82 (0.79,0.84) |
| Adeniji and Cusack (2011) | ***ICU admission*** |  |  |  |  |  |
|  | STSS score | 0.88 (0.78,0.98) | NR | NR | NR | NR |
|  | ***Mechanical ventilation*** |  |  |  |  |  |
|  | STSS score | 0.91 (0.83,0.99) | NR | NR | NR | NR |
| Miller (2010) | ***Mechanical ventilation*** |  |  |  |  |  |
|  | STSS – score 1 | 0.80 (0.70,0.89) | 0.96 (NR) | 0.44 (NR) | NR | NR |
|  | STSS – score 2 |  | 0.70 (NR) | 0.77 (NR) | NR | NR |
|  | STSS – score 3 |  | 0.26 (NR) | 0.95 (NR) | NR | NR |
|  | STSS – score 4 |  | 0.13 (NR) | 1.00 (NR) | NR | NR |
|  | ***ICU admission*** |  |  |  |  |  |
|  | STSS – score 1 | 0.82 (0.73,0.90) | 0.91 (NR) | 0.48 (NR) | NR | NR |
|  | STSS – score 2 |  | 0.72 (NR) | 0.82 (NR) | NR | NR |
|  | STSS – score 3 |  | 0.24 (NR) | 0.96 (NR) | NR | NR |
|  | STSS – score 4 |  | 0.10 (NR) | 1.00 (NR) | NR | NR |
|  | ***In hospital mortality*** |  |  |  |  |  |
|  | STSS | 0.71 (0.66,0.77) | NR | NR | NR | NR |
|  | ***Low risk of death*** |  |  |  |  |  |
|  | STSS | NR | 0.99 (NR | 0.15 (NR) | NR | NR |
| *Miller (2010)* | ***ICU admission with fatal cases not admitted to ICU excluded*** |  |  |  |  |  |
|  | STSS | 0.64 (0.58,0.71) | NR | NR | NR | NR |
|  | ***ICU admission*** |  |  |  |  |  |
|  | STSS – score 1 | 0.82 (0.73,0.90) | 0.91 (NR) | 0.48 (NR) | NR | NR |
|  | STSS – score 2 |  | 0.72 (NR) | 0.82 (NR) | NR | NR |
|  | STSS – score 3 |  | 0.24 (NR) | 0.96 (NR) | NR | NR |
|  | STSS – score 4 |  | 0.10 (NR) | 1.00 (NR) | NR | NR |
| Morton  (2015) | ***ICU admission*** |  |  |  |  |  |
|  | STSS | 0.82 (0.73,0.90) | NR | NR | NR | NR |
| Muller (2010) | ***In hospital mortality*** |  |  |  |  |  |
|  | STSS | 0.71 (0.66,0.77) | NR | NR | NR | NR |
|  | ***Low risk of death*** |  |  |  |  |  |
|  | STSS | NR | 0.99 (NR) | 0.15 (NR) | NR | NR |
|  | ***ICU admission*** |  |  |  |  |  |
|  | STSS | 0.63 (0.57,0.69) | NR | NR | NR | NR |
|  | ***ICU admission with fatal cases not admitted to ICU excluded*** |  |  |  |  |  |
|  | STSS | 0.64 (0.58,0.71) | NR | NR | NR | NR |
| Talmor (2007) | ***Mortality*** |  |  |  |  |  |
|  | STSS | 0.73, 0.80 (NR) | NR | NR | NR | NR |
|  | ***ICU admission*** |  |  |  |  |  |
|  | STSS | 0.70,0.72 (NR) | NR | NR | NR | NR |
|  | ***Mechanical Ventilation*** |  |  |  |  |  |
|  | STSS | 0.68,0.73 (NR) | NR | NR | NR | NR |
| Rowan (2010) | **Concordance statistics** (scores ≥ 0.70 are considered ‘satisfactory’) |  |  |  |  |  |
|  | ***All admissions*** |  |  |  |  |  |
|  | SWiFT core variables (highest temperature, lowest SBP, highest HR, highest RR, and neurological status) | 0.75 (0.74,0.75) | NR | NR | NR | NR |
|  | SWiFT core variables plus PaO_2_ | 0.76 (0.75,0.76) | NR | NR | NR | NR |
|  | SWiFT | 0.77 (0.76,0.77) | NR | NR | NR | NR |
|  | SWiFT core variables plus FiO_2_ and severe comorbidity in five separate organ systems | 0.77 (0.76,0.77) | NR | NR | NR | NR |
|  | SWiFT core variables plus FiO_2_ and severe comorbidity (any/none) | 0.77 (0.76,0.77) | NR | NR | NR | NR |
|  | SWiFT core variables plus FiO_2_ and severe comorbidity in five separate organ systems and age (linear) | 0.78 (0.78,0.79) | NR | NR | NR | NR |
|  | SWiFT core variables plus FiO_2_ and severe comorbidity (any/none) and age (linear) | 0.78 (0.78,0.79) | NR | NR | NR | NR |
|  | SWiFT core variables plus PaO_2_: FiO_2_ | 0.76 (0.76,0.77) | NR | NR | NR | NR |
|  | SWiFT core variables plus base excess | 0.76 (0.75,0.77) | NR | NR | NR | NR |
|  | SWiFT core variables plus urea | 0.77 (0.76,0.77) | NR | NR | NR | NR |
|  | SWiFT core variables plus FiO_2_ and urea | 0.78 (0.77,0.79) | NR | NR | NR | NR |
|  | SWiFT core variables plus FiO_2_,base excess and urea | 0.78 (0.78,0.79) | NR | NR | NR | NR |
|  | SWiFT core variables only of admissions to unit with blood lactate data | 0.75 (0.74,0.75) | NR | NR | NR | NR |
|  | SWiFT core variables only of admissions to unit with blood lactate data,plus blood lactate | 0.76 (0.76,0.77) | NR | NR | NR | NR |
|  | SWiFT core variables only of admissions to unit with blood lactate data plus FiO_2_,base excess, urea, blood lactate | 0.79 (0.78,0.79) | NR | NR | NR | NR |
|  | ***Acute exacerbations of respiratory illness*** |  |  |  |  |  |
|  | SWiFT core variables (highest temperature, lowest SBP, highest HR,highest RR,and neurological status) | 0.71 (0.70,0.72) | NR | NR | NR | NR |
|  | SWiFT core variables plus PaO_2_ | 0.72 (0.71,0.73) | NR | NR | NR | NR |
|  | SWiFT core variables plus FiO_2_ | 0.72 (0.71,0.73) | NR | NR | NR | NR |
|  | SWiFT core variables plus FiO_2_ and severe comorbidity in five separate organ systems | 0.73 (0.72,0.74) | NR | NR | NR | NR |
|  | SWiFT core variables plus FiO_2_ and severe comorbidity (any/none) | 0.73 (0.72,0.74) | NR | NR | NR | NR |
|  | SWiFT core variables plus FiO_2_ and severe comorbidity in five separate organ systems and age (linear) | 0.76 (0.75,0.76) | NR | NR | NR | NR |
|  | SWiFT core variables plus FiO_2_ and severe comorbidity (any/none) and age (linear) | 0.76 (0.75,0.76) | NR | NR | NR | NR |
|  | SWiFT core variables plus PaO_2_: FiO_2_ | 0.72 (0.71,0.73) | NR | NR | NR | NR |
|  | SWiFT core variables plus base excess | 0.72 (0.71,0.73) | NR | NR | NR | NR |
|  | SWiFT core variables plus urea | 0.73 (0.73,0.74) | NR | NR | NR | NR |
|  | SWiFT core variables plus FiO_2_ and urea | 0.74 (0.73,0.75) | NR | NR | NR | NR |
|  | SWiFT core variables plus FiO_2_,base excess and urea | 0.74 (0.74,0.75) | NR | NR | NR | NR |
|  | SWiFT core variables only of admissions to unit with blood lactate data | 0.71 (0.70,0.72) | NR | NR | NR | NR |
|  | SWiFT core variables only of admissions to unit with blood lactate data, plus blood lactate | 0.71 (0.70,0.72) | NR | NR | NR | NR |
|  | SWiFT core variables only of admissions to unit with blood lactate data plus FiO_2_,base excess, urea, blood lactate | 0.75 (0.74,0.76) | NR | NR | NR | NR |
| **Point-based (sepsis)** | | | | | | |
| Morton (2015) | ***ICU admission*** |  |  |  |  |  |
|  | MEDS | 0.77 (0.68,0.85) | NR | NR | NR | NR |
| Muller (2010) | ***Mortality*** |  |  |  |  |  |
|  | MEDS | 0.77 (0.71,0.83) | NR | NR | NR | NR |
| **Point-based (ARDS)** | | | | | | |
| Morton (2015) | ***Mechanical ventilation*** |  |  |  |  |  |
|  | P/F ratio | 0.89 (0.82,0.95) | 87.0 | 76.6 | NR | NR |
|  | ***ICU admission*** |  |  |  |  |  |
|  | P/F ratio | 0.88 (0.81,0.96) | NR | NR | NR | NR |
| **Point-based (community acquired pneumonia)** | | | | | | |
| Muller (2010) | ***Mortality*** |  |  |  |  |  |
|  | CAP | 0.65 (0.58,0.71) | NR | NR | NR | NR |
| Challen (2007) | ***In hospital mortality*** |  |  |  |  |  |
|  | CURB-65 | 0.79 (0.71,0.86) | NR | NR | NR | NR |
|  | ***ICU admission*** |  |  |  |  |  |
|  | CURB-65 ≥ 2 | NR | 65 (NR) | 93 (NR) | 95 (NR) | 56 (NR) |
|  | CURB-65 ≥ 3 | NR | 40 (NR) | 100 (NR) | 100 (NR) | 44 (NR) |
| Commons & Denholm (2012) | ***ICU admission (high risk)*** |  |  |  |  |  |
|  | CURB-65 ≥ 3 | NR | 0.11 (0.03,0.30) | 1 (0.94,1.0) | 1 (0.31,1.0) | 0.76 (0.67,0.84) |
|  | ***ICU admission (low risk)*** |  |  |  |  |  |
|  | CURB-65 0 and 1 | NR | 0.96 (0.88,0.99) | 0.26 (0.12,0.47) | 0.79 (0.69,0.86) | 0.7 (0.35,0.92) |
|  | ***ICU admission (overall)*** |  |  |  |  |  |
|  | CURB-65 | 0.73 | NR | NR | NR | NR |
|  | ***Mortality*** |  |  |  |  |  |
|  | CURB-65 | 0.66 (0.60,0.72) | NR | NR | NR | NR |
| Muller (2010) | ***Admission to high dependency care or ICU care*** |  |  |  |  |  |
|  | CURB-65 overall | 0.68 (0.64,0.72) | NR | NR | NR | NR |
|  | CURB-65 overall in H1N1 patients | 0.70 (0.63,0.77) | NR | NR | NR | NR |
|  | CURB-65 ≥ 2 | 0.62 (0.58,0.66) | 0.42 (0.35,0.50) | 0.81 (0.78,0.84) | 0.32 (0.26,0.38) | 0.87 (0.85,0.90) |
|  | CURB-65 ≥ 3 | 0.56 (0.53,0.58) | 0.14 (0.94,0.20) | 0.97 (0.96,0.98) | 0.51 (0.36,0.66) | 0.85 (0.82,0.87) |
|  | ***Mortality*** |  |  |  |  |  |
|  | CURB-65 overall | 0.71 (0.65,0.77) | NR | NR | NR | NR |
|  | CURB-65 overall in H1N1 patients | 0.73 (0.63,0.82) | NR | NR | NR | NR |
|  | CURB-65 ≥ 2 | 0.64 (0.57,0.70) | 0.48 (0.35,0.61) | 0.78 (0.76,0.81) | 0.12 (0.08,0.17) | 0.96 (0.94,0.97) |
|  | CURB-65 ≥ 3 | 0.54 (0.50,0.59) | 0.13 (0.05,0.23) | 0.95 (0.94,0.97) | 0.16 (0.07,0.29) | 0.94 (0.92,0.95) |
|  | ***Combined severe outcomes (admission + mortality)*** |  |  |  |  |  |
|  | CURB-65 overall | 0.69 (0.65,0.72) | NR | NR | NR | NR |
|  | CURB-65 overall in H1N1 patients | 0.71 (0.65,0.78) | NR | NR | NR | NR |
|  | CURB-65 ≥ 2 | 0.63 (0.59,0.66) | 0.43 (0.36,0.50) | 0.81 (0.79,0.84) | 0.35 (0.29,0.41) | 0.86 (0.84,0.88) |
|  | CURB-65 ≥ 3 | 0.56 (0.53,0.58) | 0.14 (0.09,0.19) | 0.97 (0.96,0.98) | 0.55 (0.40,0.69) | 0.83 (0.81,0.85) |
| Pereira (2012) | ***CURB-65 – ICU mortality*** |  |  |  |  |  |
|  | ≥ 1 | 0.67 (0.59,0.74) | 0.94 (0.88,0.99) | 0.17 (0.11,0.22) | 0.34 (0.28,0.40) | 0.86 (0.74,0.97) |
|  | ≥ 2 | NR | 0.73 (0.64,0.83) | 0.50 (0.42,0.57) | 0.40 (0.32,0.48) | 0.80 (0.73,0.87) |
|  | ≥ 3 | NR | 0.36 (0.26,0.47) | 0.84 (0.79,0.89) | 0.52 (0.39,0.65) | 0.74 (0.68,0.80) |
|  | ≥ 4 | NR | 0.16 (0.08,0.24) | 0.95 (0.92,0.98) | 0.63 (0.43,0.83) | 0.71 (0.65,0.76) |
|  | ≥ 5 | NR | 0 | 0.99 (0.98,1.00) | 0 | 0.68 (0.62,0.73) |
| Ryan (2020) | ***Mortality*** |  |  |  |  |  |
|  | CURB-65 (12-hr window) | 0.65 (NR) | 0.98 (NR) | 0.18 (NR) | NR | NR |
|  | CURB-65 (24-hr window) | 0.64 (NR) | 0.98 (NR) | 0.18 (NR) | NR | NR |
|  | CURB-65 (48-hr window) | 0.62 (NR) | 0.98 (NR) | 0.14 (NR) | NR | NR |
|  | CURB-65 (72-hr window) | 0.60 (NR) | 0.96 (NR) | 0.11 (NR) | NR | NR |
| Muller (2010) | ***Mortality*** |  |  |  |  |  |
|  | NHAP | 0.68 (0.62,0.74) | NR | NR | NR | NR |
| Pereira (2012) | ***PIRO-CAP – ICU mortality*** |  |  |  |  |  |
|  | ≥ 1 | 0.64 (0.58,0.71) | 0.96 (0.92,1.00) | 0.07 (0.03,0.10) | 0.32 (0.26,0.38) | 0.81 (0.62,1.00) |
|  | ≥ 2 |  | 0.94 (0.88,0.99) | 0.17 (0.11,0.22) | 0.34 (0.28,0.40) | 0.86 (0.74,0.97) |
|  | ≥ 3 |  | 0.88 (0.81,0.95) | 0.34 (0.27,0.41) | 0.38 (0.31,0.45) | 0.86 (0.78,0.94) |
|  | ≥ 4 |  | 0.58 (0.47,0.68) | 0.63 (0.56,0.70) | 0.42 (0.33,0.51) | 0.76 (0.69,0.83) |
|  | ≥ 5 |  | 0.26 (0.16,0.35) | 0.85 (0.80,0.90) | 0.45 (0.31,0.59) | 0.71 (0.65,0.77) |
|  | ≥ 6 |  | 0.04 (0.00,0.09) | 0.97 (0.95,0.99) | 0.50 (0.15,0.84) | 0.68 (0.63,0.74) |
|  | ≥ 7 |  | 0.01 (-0.01,0.03) | 0.99 (0.98,1.00) | 0.50 (-0.19,1.19) | 0.68 (0.62,0.74) |
| Muller (2010) | ***Mortality*** |  |  |  |  |  |
|  | PSI | 0.78 (0.72,0.83) | NR | NR | NR | NR |
| Commons & Denholm (2012) | ***ICU admission (high risk)*** |  |  |  |  |  |
|  | PSI ≥ 5 | NR | 0.15 (0.05,0.35) | 1 (0.94,1.0) | 1 (0.40,1.0) | 0.77 (0.68,0.85) |
|  | PSI 4 and 5 | NR | 0.41 (0.23,0.61) | 0.86 (0.76,0.92) | 0.5 (0.29,0.71) | 0.81 (0.70,0.88) |
|  | ***ICU admission (low risk)*** |  |  |  |  |  |
|  | PSI 1,2 and 3 | NR | 0.86 (0.76,0.92) | 0.41 (0.23,0.61) | 0.81 (0.70,0.88) | 0.5 (0.29,0.71) |
|  | ***ICU admission (overall)*** |  |  |  |  |  |
|  | PSI | 0.72 | NR | NR | NR | NR |
| Pereira (2012) | ***PSI – ICU mortality*** |  |  |  |  |  |
|  | ≥ Class III | 0.73 (0.67,0.78) | 0.92 (0.87,0.98) | 0.35 (0.28,0.42) | 0.40 (0.33,0.46) | 0.91 (0.84,0.97) |
|  | ≥ Class IV |  | 0.82 (0.73,0.90) | 0.53 (0.45,0.60) | 0.44 (0.36,0.52) | 0.86 (0.80,0.92) |
|  | ≥ Class V |  | 0.46 (0.35,0.57) | 0.80 (0.74,0.85) | 0.52 (0.40,0.63) | 0.76 (0.70,0.82) |
| Commons & Denholm (2012) | ***ICU admission (high risk)*** |  |  |  |  |  |
|  | SMRT-CO ≥ 4 | NR | 0.37 (0.20,0.58) | 0.97 (0.90,1.00) | 0.83 (0.51,0.97) | 0.81 (0.72,0.89) |
|  | ***ICU admission (low risk)*** |  |  |  |  |  |
|  | SMRT-CO 0,1 and 2 | NR | 0.85 (0.74,0.91) | 0.67 (0.46,0.83) | 0.88 (0.78,0.94) | 0.6 (0.41,0.77) |
|  | ***ICU admission (overall)*** |  |  |  |  |  |
|  | SMRT-CO | 0.83 | NR | NR | NR | NR |
| Muller (2010) | ***Mortality*** |  |  |  |  |  |
|  | SMARTCOP | 0.69 (0.62,0.75) | NR | NR | NR | NR |

*Published abstracts are italicized*

Abbreviations: APACHE II, Acute Physiology and Chronic Health Evaluation II; AUC, area under the curve; CAP, Severity score for the elderly with community acquired pneumonia; CATS, community assessment tools; CI, confidence interval; CURB-65, confusion, urea, respiratory rate, blood pressure, age >= 65; ED, emergency department; HR, heart rate; ICU, intensive care unit; MEDS, mortality in emergency department sepsis; MEWS, Modified Early Warning System; MSOFA, modified Sepsis-related Organ Failure Assessment; MV, mechanical ventilation; NHAP, Nursing home acquired pneumonia in the elderly; NPV, negative predictive value; NR, Not Reported; P/F ratio, arterial pO_2_ divided by the FIO_2;_ PMEWS, Pandemic medical early warning score; PPV, positive predictive value; OHPIP, Ontario health plan for an influenza epidemic; PIRO-CAP, predisposition, insult, response, and organ dysfunction in community acquired pneumonia; PSI, pneumonia severity index; qSOFA, Quick Sequential Organ Failure Assessment; RR, respiratory rate; SBP, systolic blood pressure; SMART-COP or SMRT-CO, systolic blood pressure, multilobar involvement, respiratory rate, tachycardia, confusion, oxygenation; SOFA, Sepsis-related Organ Failure Assessment; STSS, Simple Triage Scoring System; SWiFT, Swine Flu Triage.

**Supplementary Table 4.** Ethical frameworks or guiding principles for triage decisions during a pandemic.

| **Ethical frameworks** | |
| --- | --- |
| **Description** | **Author (Year published)** |
| ***Ethical principles:***  Fair:   - Let a senior doctor decide - Use pre-determined criteria determined by the Health Department - Predicted mortality - Age - Chronic comorbidity   Unfair:   - Use first come, first served approach - Use a patient’s ability to pay - Use the importance of the patient - Use random selection | Cheung (2017) |
| ***Ethical principles from most to least acceptable:***  1. Those most likely to survive  2. Those who have value to others in a pandemic  3. Those with the most years left  4. First-come, first served  5. Life cycle or "fair innings" principle  6. Lottery | Daugherty Biddison (2014) |
| ***Prioritized by healthcare providers: Ethical principles (from which should be used most often):***   1. Those most likely to survive the current illness 2. Those most likely to live the longest after recovery (considering co-morbid conditions) 3. Those that have particular instrumental value to others in a pandemic 4. Those that have lived fewer life stages 5. First come first served 6. Lottery   ***Ethical principles (from which should be used FIRST):***   1. Those most likely to live the longest after recovery (considering co-morbid conditions) 2. Those most likely to survive the current illness 3. Those that have particular instrumental value to others in a pandemic 4. First come first served   Those that have lived fewer life stages OR Lottery (same proportion)  ***Prioritized by public:***   1. Those most likely to live the longest after recovery (considering co-morbid conditions) 2. Those that have particular instrumental value to others in a pandemic 3. Those that have lived fewer life stages 4. First come first served 5. Lottery   ***Ethical principles (from which should be used FIRST):***   1. Those most likely to survive the current illness 2. Those most likely to live the longest after recovery (considering co-morbid conditions) 3. Those that have particular instrumental value to others in a pandemic 4. First come first served 5. Those that have lived fewer life stages 6. Lottery | Daugherty Biddison (2018) |
| ***Ethical considerations for triage/allocation:***   1. Resources not be held in reserve once a mass disaster protocol is in effect. 2. Disaster and pandemic policies reflect the broad consensus that there is no ethical difference between withholding and withdrawing care and that education regarding such policies be incorporated into training. 3. Triage systems based even on limited evidence are ethically preferable to those based on clinical judgment alone. 4. Critical care resources be allocated based on specific triage criteria, irrespective of whether the need for resources is related to the current disaster/pandemic or an unrelated critical illness or injury. 5. It may be ethically permissible to use exclusion criteria for critical care resources, since the advantages of objectivity, equity, and transparency generally outweigh potential disadvantages. 6. Protocols permitting the exclusion of patients from critical care during a mass disaster based on a high level of ongoing resource consumption may be ethically permissible. 7. It is ethically permissible to identify certain resource intensive therapies, procedures or diagnostic tests that should be limited or excluded during crisis standards of care. 8. Policies permitting the withdrawal of critical care treatment to reallocate to someone else based on higher likelihood of benefit may be ethically permissible. 9. Patients who do not qualify under a mass critical care (MCC) protocol for critical care receive DNR orders. 10. We suggest specific groups, e.g., healthcare workers or first responders, not receive enhanced access to scarce critical care resources when crisis standards of care are in effect. 11. Age of entry for adult critical care units be adjusted down during MCC emergencies that affect substantial numbers of children. 12. Active life-ending procedures are not ethically permissible, even during disasters or pandemics. | Daugherty Biddison (2014) |
| ***Ethical Underpinnings***   - A utilitarian approach can justifiably be adopted in determining the allocation of places (without ‘valuing’ individuals differently), albeit with reference also to the principle of ‘fairness.’ - The triage approach should be applied not only to the individual patient, but also in terms of a public health model aimed at limiting national flu mortality and morbidity. - Any ‘preference’ applied towards any class of individuals (for example, health care staff) should therefore be based predominantly upon ‘impact on aggregate flu damage,’ as well as upon the principle of ‘reciprocity’ (reflecting ‘fairness’). It should not be based upon any notion of ‘societal value’ unconnected with the epidemic and service responses to it. - In ordinary circumstances, adult critical care admits anyone who has an acute reversible problem, with decisions about admission made on an ‘individual clinical basis,’ albeit informed by aggregate statistical knowledge concerning ‘likely benefit/survival.’ The ‘alternative approach’ of the triage model will be triggered by occurrence of an ‘overwhelming need gap.’ - It can be argued that there is an ethical difference between withholding and withdrawing treatment. There is a robust counterargument that the two are ethically equivalent, most obviously in relation to the individual patient. However, in the aggregate, and specifically within triaging, this ignores the fact that the ‘likelihood of benefit’ to each individual patient intensively cared for may not be clear at the point of deciding whether or not to ‘triage in;’ more fundamentally, there is a risk in using ‘mirror image’ criteria for triaging of premature withdrawal, with rapid ‘ins and outs’ of patients, resulting in loss of overall group patient benefit. - Clinical judgment will take into account a range of clinical factors that reasonably can be considered relevant to the allocation of beds on a ‘utilitarian’ basis, but that are not amenable to measurement and therefore to explicit inclusion within a triage model. - Application of clinical judgment within triaging is open to the risk of ‘unreliability’ and ‘invalidity.’ However, this is clinically and ethically acceptable so long as factors are considered ‘honestly’ as best the clinicians can achieve. Inevitably clinicians will exercise, at least in part, not merely clinical but value judgments. Again, the presence of a clearly expressed justification for the decision combined with honest endeavor will render such decisions acceptable ethically. - The apparent ‘inequity’ of a patient remaining in critical care with less potential benefit compared to another patient competing for admission is countered by acceptance that there is bound to be a ‘social lottery’ in terms of when an individual becomes acutely unwell and what resource circumstances happen to apply at that time. - Withdrawal of care is psychologically more difficult for staff to effect than withholding care. This has implications for staff welfare. It also has ethical significance per se.   ***Practical operation of a triage model***   - Any triage system adopted should apply to all patients potentially requiring critical care; the model therefore applies to intensive care resource utilization generally during the period necessitating triage. - Patients are admitted to critical care on the basis of ‘trial of therapy,’ subject to withdrawal based upon the ‘clinical trajectory.’ Triaging decisions should be taken by more than one clinician wherever possible, ideally by at least three clinicians, at least one of whom should be an intensivist and one a nurse. - There may be advantage in separation of ‘who decides’ on the triage allocation and those delivering care; albeit this may not often be practicable. - Objective criteria should take account solely of factors likely to affect the outcome for the patient in relation to ‘this acute illness.’ Hence, a patient’s current co-morbidity would be a factor in determining (or potentially not offering) critical care where that co-morbidity impacted upon the likelihood of survival from this event. Taking account of ‘unrelated co-morbidity’ will vary according to the ‘need gap’ present at any particular point of triaging. - Clinical judgment is required to ‘allow in’ factors that cannot be part of any strict criteria but which are reasonably deemed relevant. Acceptance of the role of clinical judgment will also allow for ‘flexible operation’ of decision-making as the ‘need gap’ varies. - A chronic, non-life-threatening condition would not be considered relevant even within individual clinical judgment, given the risk of ‘devaluing particular social groups’ (for example the mentally ill), thus contravening the principle of ‘equal value’. | Eastman (2010) |
| ***Ethical values:*** Value 1: *In the context of a pandemic, the value of maximizing benefits is most important*   - Saving the most lives (Receives the highest priority) - Saving the most life-years/maximize prognosis (Receive the highest priority)   Value 2: *Treat people equally*   - First come first served (should not be used) - Random selection (used for selecting among patients with similar prognosis)   Value 3: *Promote and reward instrumental value/ benefit to others*   - Retrospective-priority to those who have made relevant contributions (gives priority to research participants and HCWs when other factors such as maximizing benefits are equal) - Prospective-priority to those who are likely to make relevant contributions (gives priority to HCWs)   Value 4: *Give priority to the worst off*   - Sickest first (used when it aligns with maximizing benefits) - Youngest first (used when it aligns with maximizing benefits such as preventing spread of the virus)   *Recommendations:*   1. The value of maximizing benefits is the most important 2. Critical interventions (testing, PPE, ICU beds, etc.) should go first to front line HCWs and others who keep critical infrastructure operating 3. For patients with a similar prognosis, equality should be invoked and operationalized through random allocation (e.g., lottery) rather than first come first served. 4. Prioritization guidelines should differ by intervention and should respond to changing scientific evidence. 5. People who participate in research to prove safety and effectiveness of vaccines and therapeutics should receive some priority for COVID-19 interventions. 6. There should be no difference in allocating scare resources between patients with COVID-19 and those with other medical conditions | Emmanuel (2020) |
| 1. Prioritizing the worst off    1. Is the patient’s condition life-threatening? Will treatment be life-saving?    2. If patient’s condition is not life-threatening, will treatment prevent permanent damage?    3. If treatment will not prevent permanent damage, will it prevent/treat temporary damage>    4. Can treatment be reasonably postponed? 2. Maximizing benefits (i.e., saving the most lives or the most life-years 3. Treating patients equally (e.g., lottery, but only for patients with similar prognoses) 4. Promoting instrumental value (i.e., benefit to society) | Hulsbergen (2020) |
| ***Ethical Framework:***   1. Duty to care 2. Duty to steward resources 3. Duty to plan 4. Distributive justice 5. Transparency   ***Clinical Protocol:***   1. Pretriage requirements (e.g., surge capacity and planning) 2. Patient categories (based on clinical factors applied to all patients, not just the ones with pandemic influenza) 3. Acute versus chronic care facilities (the latter potentially being exempt from acute triage guidelines) 4. Clinical evaluation (based on OHPIP) 5. Triage decision makers (triage officer) 6. Palliative care (prepare for more; follow facility guidelines) 7. Review of triage decisions (daily retrospective review) 8. Communication (patients, families) | Powell (2008) |
| **Ethics - Rationing decisions should not consider or be based upon:**   - Race, ethnicity, gender, gender identity and expression, sexual orientation or preference, religion, citizenship, or socioeconomic status - Age as a criterion in and of itself (this does not limit consideration of a patient’s age in clinical prognostication of likelihood to survive to hospital discharge) - Ability to pay - Disability status as a criterion in and of itself (this does not limit consideration of a patient’s physical condition in clinical prognostication of likelihood to survive to hospital discharge) - First-come, first-served - Judgments that some people have greater “quality of life" than others - Judgments that some people have greater “social value” than others | Prekker (2020) |
| **Ethical Principles**  1. Exceptionality  2. Transparency and disclosure  3. Trust  4. Equity  5. Greater benefit for the greater number  6. Flexibility and temporality  **Procedural Aspects:**   - Decisions must be consensual and shared among professionals. - Medical team would be responsible for evaluating the patient and examining their baseline. The team would perform a preliminary assessment on who would or would not be a candidate for advanced life support. | Real de Asua (2020) |
| ***Ethical framework:***   1. Medical versus social utility (of the patient), narrow versus broad criteria 2. Utilitarian ("triage decisions regarding the provision of critical care should be guided by the principle of seeking to help the greatest number of people survive the crisis") versus egalitarian (assisting those who are salvageable, but in the greatest need-can be first-come, first-served or random selection). Suggest a hybrid and adjust according to the epidemiological data.   Triage Review Board-to oversee switch from traditional ethics of individual autonomy to an ethics of public health | Tabery and Mackett (2008) |
| ***Ethical framework:***   1. Equity of access 2. Organization triage (inclusion/exclusion criteria and range of scores) versus treatment triage (identify patients most or least likely to benefit from ICU care) -> flexible triage depending on supply and demand on resources that is based in evidence   Treatment triage and Utilitarianism (survival for the greatest number of people) Prioritization and respect | Tillyard (2010) |
| **Recommendations or guiding principles** | |
| ***Recommendations:***   1. Establishing an Incident Management System with Emergency Executive Control Groups at facility, local, regional/state or national levels to exercise authority and direction over resources; 2. Developing fair and equitable policies may require restricting ICU services to patients most likely to benefit; 3. Usual treatments and standards of practice may be impossible to deliver 4. ICU care and treatments may have to be withheld from patients likely to die even with ICU care and withdrawn after a trial in patients who do not improve or deteriorate 5. Triage criteria should be objective, ethical, transparent, applied equitably and be publicly disclosed 6. Trigger triage protocols for pandemic influenza only when critical care resources across a broad geographic area are or will be overwhelmed despite all reasonable efforts to extend resources or obtain additional resources 7. Triage of patients for ICU should be based on those who are likely to benefit most or a ‘first come, first served’ basis 8. A triage officer should apply inclusion and exclusion criteria to determine patient qualification for ICU admission | Christian (2010) |
| ***Task force suggestions:***   1. In the event of an incident with mass critical care casualties, we suggest all hospitals within a defined geographic/administrative region (e.g., state), health authority, or health-care coalition should implement a uniform triage process and cooperate when critical care resources become scarce. 2. We suggest critical care only be rationed when resources have, or will shortly be, overwhelmed despite all efforts at augmentation and a regional-level authority that holds the legal authority and adequate situational awareness has declared an emergency and activated its mass critical care plan. 3. We suggest health-care systems provide oversight for any triage decisions made under their authority via activation of a mass critical care plan to ensure they comply with the prescribed process and include appropriate documentation. 4. We suggest health-care systems that have instituted a triage policy have a central process to update the triage protocol/system so that information that becomes available during an event informs the process in order to promote the most effective allocation of resources. 5. We suggest health-care systems establish in advance, a formal legal and systematic structure for triage in order to facilitate effective implementation of triage in the event of an overwhelming disaster. 6. We suggest health-care systems that have instituted a triage policy to triage patients based on improved incremental survival rather than on a first-come, first-served basis when a substantial incremental survival difference favors the allocation of resources to another patient. 7. Triage officers: a) We suggest health-care systems that have instituted a triage policy have clinicians with critical care triage training function as triage officers (tertiary triage) to provide optimum allocation of resources. b) We suggest triage officers should have situational awareness at both a regional level and institutional level. c) We suggest in trauma or burn disasters, triage be carried out by triage officers who are senior surgeons/ physicians with experience in trauma, burns, or critical care and experience in care of the age-group of the patient being triaged. d) We suggest in environments where triage is not usual, individual triage officers or teams consisting of a senior intensive care physician and an acute care physician be designated to make mass critical care triage decisions in accordance with previously prepared, publicly vetted, and widely disseminated guidelines. e) We suggest in limited resource settings in which there is a limited need for expansion of critical care resources, a continuation of well-established systems is appropriate. 8. We suggest triage protocols (clinical decision support systems), rather than clinical judgment alone, be used in triage whenever possible. 9. We suggest in health-care systems that have instituted a triage policy, technology such as baseline ultrasound, oxygen saturation as measured by pulse oximetry, mobile phone/Internet, and telemedicine be leveraged in triage where appropriate and available to augment clinical assessment in an effort to improve incremental survival and efficiency of resource allocation. 10. We suggest triage decision processes, whenever possible, provide for an appeals mechanism in case of deviation from an approved process (which may be a prospective or retrospective review) or a clinician request for reevaluation considering novel or updated clinical information (prospective). 11. Triage process:   a. We suggest tertiary-care triage protocols for use during a disaster that overwhelms or threatens to overwhelm resources be developed with inclusion and exclusion criteria.  b. We suggest the inclusion criteria for admission to intensive care. c. We suggest patients who will have such a low probability of survival that significant benefit is unlikely be excluded from ICUs when resources are overwhelmed. d. We suggest consideration be given to excluding patient groups that have a life expectancy of 1 year. e. We suggest if a physiologic (nondisease-specific) outcome prediction score can be demonstrated to reliably predict mortality in a specified population upon screening for ICU admission, it is reasonable to use this to exclude admission for patients with a predicted mortality rate of 90%. Similarly, if a disease- specific score can be demonstrated to reliably predict mortality when used in the same manner for patients with the disease, we suggest it is reasonable to use this to exclude admissions for patients with a predicted mortality rate of 90%. f. We suggest each patient’s condition be reassessed after a suitable time period (e.g., 72 h) by the triage officer or triage team. If at that point the patient meets the criteria for exclusion from ICU, consideration should be given to withdrawal of therapy. If in the future a score is demonstrated to reliably predict high mortality when the patient is assessed during ICU stay, this should be used in preference to or as a supplement to clinical judgment. | Christian (2014) |
| ***Guiding Principles for Allocating Antibiotics During a Pandemic:***   - Professional obligations to individual patients; - Professional and institutional obligations of competence; - Professional and institutional obligations of honesty and transparency; - Distributive justice, including equal treatment, utility; - Fair procedures, including in planning and implementation; and - Accountability and legitimacy *Overall:* Committee felt "First come first serve" until antibiotics gone was unacceptable Generally, antibiotics should be allocated to the sickest patients and to those who have the greatest likelihood for survival | Cinti (2009) |
| ***Task Force suggestions:*** 1. An equitable triage process utilizing the SOFA scoring system 2. The concept of triage by a senior clinican(s) without direct clinical obligation and a support system to implement and manage the triage process 3. Legal and ethical constructs underpinning the allocation of scarce resources 4. A mechanism for rapid revision of the triage process as further disaster experiences, research, planning and modeling come to light | Devereaux (2008) |
| ***Triage for special populations:*** We suggest triage and resource allocation of special populations (defined for mass critical care to be 'those patients that may be at increased risk for morbidity and mortality outside a fully functional critical care environment or those patients that present unique challenges to providers when a full complement of supportive services is no available. We include the chronically ill and technologically dependent as the fragility of their baseline health puts them at significant risk for progressions to a higher level of medical need) adhere to the same resource allocation strategy and process as the general population. | Dries (2014) |
| ***Principle of reverse triage:*** Identify patients for whom expedited discharge is safe and ethical. This includes daily evaluation of potential for discharge to intermediate or floor-level care. Availability of intermediate and floor care beds are critical to the success of reverse triage. Reverse triage is at the discretion of the critical care physicians. | Einav (2014) |
| ***AGS Recommendations for Resource Allocation Strategies if Emergency Rationing is Required***   1. Avoiding age per se as a means for excluding anyone from care 2. Assessing comorbidities and considering the disparate impact of social determinants of health 3. Encouraging decision makers to focus primarily on potential short term (not long-term) outcomes 4. Avoiding ancillary criteria such as life-years saved and long-term predicted life expectancy that might disadvantage older people 5. Forming and staffing triage committees tasked with allocating scarce resources 6. Developing institutional resource allocation strategies that are transparent and applied uniformly 7. Facilitating appropriate advance care planning | Farrell (2020) |
| ***Decision-making model for clinicians based on beneficence and justice:***   1. Formation of a multidisciplinary pandemic triage committee (act as a neutral and impartial entity serving as the supervising body when making resource allocation decisions and would regularly review current advances and decisions within state, federal and international organizations) 2. Phased allocation of resources 3. Clinical evaluation: Pandemic triage, also must include non-influenza patients    - Level 1-pandemic is present, but resources are not exhausted    - Level 2-pandemic is present, and resources are at capacity    - Level 3-pandemic is present, and resources are over capacity    - Within each level: inclusion criteria and exclusion criteria and objective score criteria (Adapted from OHPIP guidelines) 4. Checklist of clinical progress (one-page document at front of patient chart) 5. Palliative care protocol (when patient does not qualify for critical care allocation) 6. Appeals process (for families or clinical providers) 7. Early family involvement (family made aware of clinical decision model, including criteria, from the beginning) | Lin and Anderson-Shaw (2009) |
| **The New York City Pandemic Resuscitation Equitable Allocation Principles.**  1. Triage decisions related to resource allocation and advanced resuscitation need to be based on real-time assessments and balancing of the following dynamic factors that are always present in emergency care settings and at critical levels during disasters:  a. Patient factors (chance of survival and patient/family preference)  b. People (current patient volume/acuity and available skilled staff)  c. Hospital capacity factors (environment, space, needed equipment, medications, resources, and the ability to protect frontline staff)  2. Triage decisions will not be based on race, ethnicity, gender, disability, insurance status, immigration status, social class, or other non-clinical factors.  3. There should be no categorical exclusions from advanced resuscitation/ventilator access — with two exceptions:  a. Patients/families expressed wishes for Do Not Resuscitate (DNR) or Do Not Intubate (DNI)  b. Patients with medical or traumatic conditions expected to result in immediate or near-immediate mortality even with aggressive therapy  4. All patients should have their wishes for care and/or DNR/DNI status respected and be provided with desired resources, including comfort measures, palliative care, hospice, and, if at all possible, the opportunity to be with or communicate with their loved ones.  5. Age (within pediatric or adult category) will not be used in triage decisions, except as is clinically relevant in determining relative likelihood of immediate survival.  6. Emergency systems of care and hospitals/health systems must do everything possible to anticipate, coordinate, and plan ahead to avoid resource constraints. They must also provide clear communication and anticipatory guidance for frontline providers and timely access to crisis triage teams. Waiting until available resources and staff are overwhelmed will jeopardize the health of both patients and frontline staff.  7. Emergency systems of care and hospitals/health systems have an imperative to protect the safety and health of frontline providers caring for the patient population.  8. Frontline providers and Crisis Triage Teams should convene on a weekly basis, or more frequently as needed during disasters, to debrief with team members, review prior triage decisions, and consider new data, changes in trends in clinical outcomes, and emerging information about treatment effectiveness that might alter the prioritization process.  9. We must plan for de-escalation and provide for mental health aftercare of frontline staff who may experience PTSD, burnout, and moral distress during disasters. | Rhodes (2020) |
| ***Working group on emergency mass critical care planning assumptions and recommendations:***   - Triage decisions regarding critical care should aim to help the greatest number of people, including those who were already in the ICU before the disaster. - Give interventions first to the people deemed most likely to survive OR give all patients a trial of essential elements of critical care and "withdraw critical care from those who do not clinically improve after a set period of time” - "Senior intensivists and critical care nurses designated to make decisions regarding when to initiate or withhold critical care services” - Hospitals should establish procedure in advance of a crisis. These protocols should be developed regionally and with input from stakeholders (including the public). | Rubinson (2005) |
| ***Guiding principles:***   1. Utility ("do the best good for the most people") 2. Equitable treatment for all persons (i.e., human rights and dignity) 3. Maximize use of resources 4. Treat those in the greatest need of medical attention 5. Treat those who are most likely to survive medical treatment 6. First-come-first serve 7. Resources allocated based on societal contributions (e.g., caregivers, healthcare providers - feelings of reciprocity) 8. Pre-crisis planning 9. Include perspectives from non-physicians in triage decision making 10. Involve public in pandemic planning (creates buy-in, understanding, and provides education on why which decisions are made). | Silva (2012) |
| ***Ethical Triage Criteria:***   - We take the following three general principles as evaluative references:   a. The good of a single patient should be considered in the framework of the common good.   - - Common good means the good of all people and of the whole person. It is rooted in the idea of human dignity, which gives birth to the humanitarian imperative conveyed in the first core principle of “disaster medicine”; the common good also means that, in a Global Health framework, patients are not just isolated individuals but persons with strong ties to their communities, and therefore both patient and community need to be taken into account;   b. No one must be abandoned or discriminated against for any reason;  c. Before denying a necessary referral of a patient to an ICU, due to lack of resources, it is required to consider alternatives both for the immediate case and, based on the experience gained, for similar future cases.   - Appropriate assistance to any person in need of medical care should be provided whenever possible. In critical situations, the criteria for determining priority are the urgency and severity of the clinical situation. Consideration should also be given to the effectiveness and proportionality of the medical intervention, with the goal of obtaining the greatest possible benefit for the greatest number of patients. - Triage must be carried out on a case-by-case basis, with reference not only to the patient’s clinical condition but also to the availability of resources in the hospital. Possible transfer initiatives to other larger and better resourced national or foreign intensive care units must also be considered. Triage must not proceed using a standardized approach where the sole decision-making criteria is age. - Inappropriate treatments are not acceptable. - Adequate forms of palliative and spiritual care must be assured, where necessary. | Tambone (2020) |
| ***Supplementary triage criteria: key considerations***   - Is the criterion likely to contribute to the overall objective: to assist healthcare providers in continuing to provide patient-centered care given the circumstances following from the provincially mandated adoption of a population focus in a pandemic, and the parameters of the OHPIP that recognizes resources are limited under such circumstances - Are infringements of rights or freedoms “sufficiently important” (must relate to concerns that are “pressing and substantial”), reasonable and justified? - Can the criterion be practically implemented given the circumstances of a pandemic? - Does the criterion reflect widely held values and moral intuitions, such as those in the Ontario Health Plan for an Influenza Pandemic ethics framework? - Does the criterion violate widely held values as little as possible? - Does the criterion strike a balance between individual liberty and community interests? - Does the criterion adequately distinguish between broad social utility (social utility independent of the pandemic scenario) and narrow social utility (social utility related specifically to the pandemic)? - Is the criterion applicable to the pediatric population? | Winsor (2014) |
